# Mesenchymal Stem and Progenitor Cells in Normal and Dysplastic Hematopoiesis—Masters of Survival and Clonality?

**DOI:** 10.3390/ijms17071009

**Published:** 2016-06-27

**Authors:** Lisa Pleyer, Peter Valent, Richard Greil

**Affiliations:** 1 3rd Medical Department with Hematology and Medical Oncology, Hemostaseology, Rheumatology and Infectious Diseases, Laboratory for Immunological and Molecular Cancer Research, Oncologic Center, Paracelsus Medical University Salzburg, 5020 Salzburg, Austria; r.greil@salk.at; 2 Center for Clinical Cancer and Immunology Trials at Salzburg Cancer Research Institute, 5020 Salzburg, Austria; 3 3rd Medical Department, Cancer Cluster Salzburg, 5020 Salzburg, Austria; 4 Department of Internal Medicine I, Division of Hematology and Hemostaseology & Ludwig Boltzmann Cluster Oncology, Medical University of Vienna, 1090 Vienna, Austria; peter.valent@meduniwien.ac.at

**Keywords:** mesenchymal stem cells (MSC), myelodysplastic syndromes (MDS), acute myeloid leukemia (AML), microenvironment, neoplastic stem cells, bone marrow stem cell niche, leukemic niche, immunomodulation, epithelial-to-mesenchymal transition, endothelial-to-mesenchymal transition

## Abstract

Myelodysplastic syndromes (MDS) are malignant hematopoietic stem cell disorders that have the capacity to progress to acute myeloid leukemia (AML). Accumulating evidence suggests that the altered bone marrow (BM) microenvironment in general, and in particular the components of the stem cell niche, including mesenchymal stem cells (MSCs) and their progeny, play a pivotal role in the evolution and propagation of MDS. We here present an overview of the role of MSCs in the pathogenesis of MDS, with emphasis on cellular interactions in the BM microenvironment and related stem cell niche concepts. MSCs have potent immunomodulatory capacities and communicate with diverse immune cells, but also interact with various other cellular components of the microenvironment as well as with normal and leukemic stem and progenitor cells. Moreover, compared to normal MSCs, MSCs in MDS and AML often exhibit altered gene expression profiles, an aberrant phenotype, and abnormal functional properties. These alterations supposedly contribute to the “reprogramming” of the stem cell niche into a disease-permissive microenvironment where an altered immune system, abnormal stem cell niche interactions, and an impaired growth control lead to disease progression. The current article also reviews molecular targets that play a role in such cellular interactions and possibilities to interfere with abnormal stem cell niche interactions by using specific targeted drugs.

## 1. Introduction

A very small population of pluripotent hematopoietic stem cells (HSCs) is at the apex of the hematopoietic developmental hierarchy and has the capacity to replenish all other blood-cell lineages for unlimited time periods [1]. HSCs also have the ability to self-renew and to reconstitute hematopoiesis after transplantation. During differentiation, the progeny of HSCs develop through various intermediate maturational stages, including multi-potential and lineage-committed progenitor cells [2]. These hematopoietic stem and progenitor cells (HSPCs) reside in protected stem cell (SC) niches in the bone marrow (BM), and are only rarely present in the peripheral blood under normal conditions. HSPCs can give rise to myeloid lineages, lymphoid lineages, or both. Myeloid lineages include monocytes, macrophages, neutrophils, basophils, eosinophils, erythrocytes, dendritic cells (DCs), mast cells, megakaryocytes, and platelets, whereas lymphoid lineages include T-cells, B-cells, natural killer T-cells (NKTs), and natural killer cells (NKCs).

The term “myelodysplastic syndromes” (MDS) encompasses a heterogeneous group of clonal disorders characterized by increased but ineffective dysplastic hematopoiesis, resulting in peripheral cytopenias [3]. MDS are the most common hematologic malignancies and mostly affect the elderly [4]. About 30%–45% of patients eventually progress to acute myeloid leukemia (AML). In real-life cohorts, approximately two-thirds of elderly patients with AML present with MDS-related changes [5,6]. Despite intense research, the cell of origin in MDS and AML remains speculative. Both diseases are thought to originate from a hierarchy of neoplastic SC classes that differ in self-renewal capacity [7] and arise from transformed normal HSCs or from hematopoietic progenitor cells that re-acquire self-renewal capacity during transformation and then act as neoplastic SCs (NSCs), also referred to as leukemic SCs (LSCs) in the context of leukemia, such as AML [8]. In this article we will use the term “NSCs” to collectively refer to disease-initiating and -propagating primitive cells in MDS and AML. NSCs can be studied through their ability to engraft and establish MDS or AML in immunodeficient mice. Depending on the type of MDS or AML, NSCs are enriched in the CD34^+^CD38^−^ or CD34^+^CD38^+^ fraction of the clone, and give rise to additional NSCs as well as (more mature) blast cells that lack engraftment potential [9,10].

Early-stage MDS is characterized by enhanced apoptosis, increased phagocytosis, and reduced differentiation of affected cells [11], resulting in peripheral cytopenia(s) despite BM hypercellularity. Cytopenias can predispose these patients to potentially life-threatening complications such as bleeding or infections, which are the most common causes of death in MDS and AML [12,13]. It currently remains unclear whether increased apoptosis results from a desperate immune reaction directed against MDS/AML-associated antigens or MDS/AML-specific neo-antigens [14] expressed by the malignant clone, or whether apoptosis represents an integral part of the pathophysiology of MDS (summarized in [3]). During disease progression to late-stage MDS and AML, a reversal of the above-mentioned phenomenon occurs, resulting in reduced programmed cell death (apoptosis) and impaired cell removal by phagocytosis. The latter occurs via up-regulation of the antophagocytic marker CD47 (“do not eat me signal”) on myeloid progenitors, which has been identified as an important pro-oncogenic transition step leading from low-risk MDS to high-risk MDS and possibly to AML [11]. In addition, the acquisition of genetic mutations during disease progression results in a block in differentiation and increased proliferative potential of clonal cells in late-stage MDS and AML. This results in expansion of the malignant clone and coincides with a transition from (initial partially active) immune surveillance to immune subversion, and ultimately immune escape [15] of the dysplastic/leukemic clone. NSCs exert a plethora of immunomodulatory and immunosuppressive effects on various immunocompetent cells (summarized in [3]). Vice versa, the immune system is considered to be involved in the regulation of growth and survival of NSCs (recently reviewed in [16]).

MDS are not only characterized by peripheral cytopenias and the presence of clonal blast cells in the BM, but also by alterations in the BM microenvironment. In particular, the BM microenvironment is severely disturbed in MDS and AML and supposedly contributes to disease evolution and progression [17,18,19,20,21,22,23,24,25,26,27,28,29,30]. Every stage of disease initiation, propagation, and progression from early-stage (low-risk) MDS to late-stage (high-risk) MDS and AML may be defined by specific events relevant to the communication between the dysplastic/leukemic clone and the pathologically altered microenvironment (reviewed, e.g., in [16]). Indeed, relevant structural, epigenetic, quantitative, and functional alterations of BM stromal components are present in patients with hematologic malignancies, including MDS and AML [31,32,33,34,35].

Various stromal cells are considered to form the supportive microenvironment of the BM, the so-called niche, in which HSPCs can grow and undergo differentiation and maturation [36]. Stromal cells forming the SC niche in the BM include mesenchymal SCs (MSCs), adipocytes, osteoblasts, fibroblasts, endothelial cells (ECs), tissue macrophages, and osteoclasts. Many of these cells are derived from MSCs or from HSPCs. MSCs are adult, fibroblast-like multipotent cells essentially involved in maintaining the microenvironment and thus tissue homeostasis. Human mesenchymal stem and progenitor cells (MSPCs) can be derived from the BM, umbilical cord blood, placenta, or adipose tissue [37]. In the BM, MSPCs are involved in structure formation and organization of the hematopoietic microenvironment [38,39,40].

It is not our aim to reconcile all the open debates on the topics discussed below, as this is beyond the scope of this review. Rather, our aim is to walk the reader through the maze of literature on a central thread, with the goal of gaining a better understanding of MSCs, where they may come from, where they are localized, and what they do, with a specific focus on the known and assumed function and role of MSPCs and their progeny in the normal, dysplastic, and leukemic BM.

## 2. MSCs: Nomenclature

The term “MSCs” encompasses a heterogeneous population of cells that covers several subsets of MSCs with different phenotypes and functions [41,42]. MSPCs were traditionally isolated based on plastic adherence properties [43]. While *bona fide* MSCs do exist, not all fibroblast-like plastic adherent cells meet generally accepted criteria of MCS, including SC activity. However, the acronym “MSCs” is widely used for both cell populations, which may be misleading [44]. In order to be more accurate regarding nomenclature, the International Society for Cellular Therapy (ISCT) position statement encouraged the scientific community to use the term “mesenchymal SCs” only for cells that meet specified SC criteria, while those that do not should be termed “multipotent mesenchymal stromal cells” [45]. In analogy to the terms “HSPCs” and “LSPCs”, we will use the term “MSPCs” to refer to mesenchymal stem and progenitor cells in this article.

## 3. MSPCs: Phenotypic Characterization and “Plasticity”

Minimal criteria for the characterization of MSPCs have been defined by the “Mesenchymal and Tissue Stem Cell Committee” of the ISCT: MSPCs must be plastic-adherent in culture; must express *CD73*, *CD90*, and *CD105*; must lack expression of *CD11b*, *CD14*, *CD34*, *CD45*, *CD79α*, and human leukocyte antigen-D-related (*HLA*-*DR*) cell surface antigens; and must have the capacity to differentiate into osteoblasts, adipocytes, and chondroblasts in vitro [43]. In addition, MSCs supposedly have the ability to differentiate into fibroblasts, adipocytes, chondroblasts, osteoblasts, and/or tissue macrophage-like cells [38,46], and possibly also into ECs [47]. Under certain culture conditions, MSCs may also transdifferentiate to tissues of neuro-ectodermal origin such as neurons or glial cells. However, differentiation pathways and lineages from MSPCs are not always strictly separable, since even apparently fully differentiated progeny cells from a given lineage retain the capacity to switch into another lineage (phenotypic “plasticity”) and many intermediate cells with overlapping phenotypes and functions are observed [48,49]. For example, it has been reported that some human stromal cells display the morphology of an adipocyte at one pole of the cell, and a vascular smooth muscle morphology at the other pole [50]. MSPC plasticity is thought to be governed by epigenetic mechanisms and, very recently, methylation patterns have been used to assist the classification of MSPCs [51].

Today we know that heterogeneous populations with different degrees of “stemness” and only some MSPC populations exhibit full SC function, including the capacity of long-term self-renewal. One key problem in the definition of MSCs is that due to a lack of specific markers, it is difficult to distinguish MSCs from other, more mature stromal cells such as fibroblasts, which, conversely, are abundant in mesenchymal tissue [52]. Therefore, multiple markers have to be applied to detect and isolate MSCs [53,54,55]. Self-renewing, multipotent populations of human BM-MSPCs may express *Stro1*, *3G5*, *CD51*, *CD146*, *CD271*, stage-specific embryonic antigen-4 (*SSEA*-*4*), platelet-derived growth factor-α (*PDGFRα*), SC factor (*SCF*), *Nestin*, and *TWIST* [43,56,57,58,59]. Several of these markers may define distinct MSPC populations, and a certain phenotypic and functional overlap may also exist [60].

Cell isolation procedures and cell culture conditions have been shown to influence the expression of MSPC surface markers, which likely explains the differences observed between laboratories. In this regard, down-regulation, up-regulation, and (neo)acquisition of cell surface markers on MSPCs have been discussed. Changes in the marker profile may also occur when MSPCs differentiate during in vitro culture [41,42,60]. Moreover, phenotypic heterogeneity of MSPCs has been related to the different origins (tissues) and various procedures of isolation of these cells [61]. In addition, some of the above-mentioned “stemness” markers may be differentially expressed on human fetal and adult BM-MSPCs [62]. Despite the proposal provided by the ISCT [43,63], these standards have not been widely adopted, and criteria for MSPC isolation and identification continue to vary, making cross-study comparison difficult [56,59,60,63,64,65]. However, there is consensus regarding the necessity to precisely define the phenotypes of human MSPCs in order to guarantee harmonization of experimental protocols and comparable isolation procedures for MSPCs in various organ systems [64].

### Abnormal Phenotype of MSPCs in MDS and AML

In patients with MDS, MSPCs show decreased expression of certain cell surface molecules [66], especially those involved in the interaction with HSPCs [33], including the adhesion molecules CD44 and CD49e (α5-integrin), both of which are involved in directing primary human NSCs to MSPCs (in vitro) [67]. Lack of CD44 and CD49e combined with the absence of HSPCs has been correlated with growth deficiencies of MDS-MSPCs, suggesting that an interaction between MSPCs and hematopoietic cells is necessary for healthy MSPC proliferation [68]. CD44 binds the extracellular matrix proteins hyaluronan, osteopontin, and E-selectin, and mouse models have shown that CD44 is critical for directing AML cells to the leukemic niche [69]. In addition, CD44 has been implicated in the repopulation capacity of human leukemic (stem) cells in murine xenograft models [69], chemoresistance [70], and disease relapse [71]. Initial in vitro [67] and in vivo [72,73] data indicate that CD44 is of particular relevance in human AML. Therapeutic blocking of CD44 in AML cells has been evaluated in murine xenograft settings, with some promising initial results [69]. However, in vitro co-culture experiments have shown that BM stromal cells find a way to protect NSCs from this type of targeted therapy [74]. In addition, the LSC niche has been shown to be physically distinct and independent of the constraints that apply to normal HSCs [75]. Thus, NSCs may no longer be absolutely dependent on the BM niche in advanced-stage AML, which likely explains why targeting of CD44 has not yet been successfully implemented in early-phase clinical trials in this disease. Several phase 1 clinical trials testing an antibody against CD44v6 (bivatuzumab mertansine) in solid tumor entities are underway, but no clinical trials are listed so far at clinicaltrials.gov in the indication of MDS or AML [76], despite reported in vitro activity of targeting CD44v6 [77].

Direct cell-to-cell contact between HSPCs and MSPCs is required for maintenance of “stemness”, and focal adhesion proteins are known to be involved in cancer survival signal transduction. In this regard, signaling via focal adhesion proteins such as HSP90α/β has been implicated in HSPC-MSPC interactions in patients with advanced-stage MDS, providing MDS-MSPCs with a proliferative advantage while negatively impacting on clonogenicity of HSPCs [78]. As such, HSP90α/β inhibition might be investigated as a potential therapeutic target in MDS.

Alterations in MSPC phenotype and function are more prominent in AML and late-stage (high-risk) MDS as compared to early-stage (low-risk) MDS [30,79,80,81], suggesting a direct correlation between the degree of MSPC impairment and the disease severity.

## 4. MSPCs: Cell of Origin

**Figure 1:**
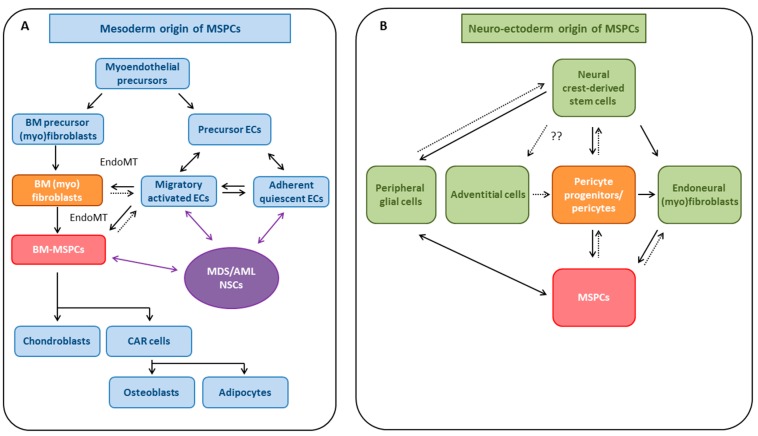
Presumed ontogenetic origin of mesenchymal stem and progenitor cells (MSPCs). MSPCs are a heterogeneous mixture of subpopulations, which may derive from differing developmental origins. (**A**) Model for mesoderm origin of MSPCs. Blood vessel-derived myoendothelial precursor cells may give rise to bone marrow (BM) precursor myofibroblasts as well as precursor (proto) endothelial cells (ECs), which can shift between a quiescent endothelial and a proliferative migratory phenotype. The latter may (trans)differentiate into BM-MSPCs via endothelial-to-mesenchymal transition (EndoMT) either directly, or indirectly via BM (myo)fibroblasts. MSPCs also stabilize and deliver pro-survival and maturation signals to ECs. MSPCs give rise to three mesodermal lineages in vitro, namely chondroblasts, osteoblasts, and adipocytes. The latter two derive from CXCL12^+^ abundant reticular (CAR) cells with bi-lineage (adipo-osteogenic) potential. Myelodysplastic syndromes/acute myeloid leukemia neoplastic stem cells (NSCs) interact with and modulate MPSCs, as well as both migratory-activated and adherent quiescent ECs; (**B**) Model for neuro-ectoderm origin of MSPCs. Neural crest-derived stem cells (NCSCs) are thought to give rise to (i) peripheral glial cells (a process which may be reversible as indicated by dotted arrows); (ii) endoneural (myo)fibroblasts either directly or indirectly via development from (iii) pericyte progenitors/pericytes (in the brain and the central nervous system), all of which can give rise to MSPCs (a process which may be reversible as indicated by dotted arrows). Possibly pericytes may also develop from adventitial cells. Solid arrows represent differentiation pathways. The dotted arrows represent differentiation pathways as presumed from indicative evidence, and for which further proof is needed.

The cell of origin for MSPCs currently remains unclear, and both a mesodermal origin as well as a neuro-ectodermal origin and even a dual origin have been suggested [49]. It has been shown by several groups that human blood vessel-derived SC/precursor populations (i.e., myogenic ECs [82], pericytes [39,83], and adventitial cells [84,85]) may give rise to *bona fide* MSPCs, suggesting that the vasculature serves as a systemic reservoir of MSPC-like cells [86,87,88]. The three MSPC precursor subsets are located in the three structural regions of typical blood vessels: intima, media, and adventitia. They all display typical MSC markers and possess robust mesenchymal multipotency and differentiation capacity in culture, thus meeting the criteria of *bona fide* MSPCs (recently reviewed in [86]). We have summarized current knowledge on the presumed ontogenetic origin of MSPCs in Figure 1.

### 4.1. Evidence for Mesodermal Origin of MSPCs

As discussed above, MSPCs per definition must give rise to three mesodermal lineages in vitro, namely osteoblasts, chondrocytes, and adipocytes. Ontological evidence suggests that the lateral plate mesoderm (LPM) serves as a potential common source of these three cell types, suggesting that MSPCs may also be of mesodermal origin [89]. In line with this hypothesis, the LPM and MSPCs co-express several marker genes such as α-smooth muscle actin (*αSMA*), *TWIST* or others. The LPM differentiates by undergoing endothelial-to-mesenchymal transition (EndoMT), thus generating a population of more differentiated but still immature mesenchymal cells, and MSPCs may develop depending on the tissue environment (reviewed by Sheng et al. [89]).

The mesenchymangioblast was recently identified as the common embryonic precursor for both ECs and mesoderm-derived MSPCs [90], and other data indicate that embryonic ECs can also give rise to MSPCs via transdifferentiation [47]. The mesodermal origin of MSPCs is further supported by the observation that myoendothelial cells differentiate into myogenic, osteogenic, and chondrogenic cells in appropriate culture conditions, and thus behave similarly when compared to MSPCs [82]. Additional insights into the origin of MSPCs from the mesoderm show EndoMT to be a critical step in MSPC formation [90,91] (Figure 1A).

It has been established that ECs arise from mesodermal progenitors [92]. Munoz-Chapuli et al. presented a new ontogenetic view on the origin of ECs that was compatible with several ontogenetic models, including the hemangioblast [93,94,95,96], circulating endothelial precursors, and hemogenic endothelium [95,97,98], as well as the bipotential vascular progenitor [99]. According to this hypothesis, vertebrate endothelium originates from a layer of circulating specialized blood cells (“hemocytes” or “amoebocytes”) that first became adherent, and later endothelial, with the transition between hemocytes and endothelium occurring via the acquisition of an endothelial phenotype [92,100]. These proto-endothelial cells are thought to retain the ability to transiently shift between a quiescent, endothelial phenotype and a proliferative, migratory, and invasive phenotype, when stimulated by specific signals [92] (Figure 1A).

Of interest, Gunsilius et al. found that chronic myeloid leukemia may arise from a bipotent hemangioblast present in adult humans [96,101].

#### 4.1.1. EndoMT as a Potential Source for MSPCs

Recently, EndoMT has been recognized as a new type of cellular (trans)differentiation process capable of generating MSPCs [91] (for review see, e.g., [102]). It has also been described that EndoMT is a complex biological process in which resident ECs lose cell-cell junctions as well as their specific endothelial markers and acquire a mesenchymal or myofibroblast-like phenotype (e.g., αSMA, vimentin, and type I collagen). During this process, cells become motile and are capable of migrating into surrounding tissues. Similar to epithelial-to-mesenchymal transition (EMT), EndoMT can be induced by snail-mediated tumor growth factor β (TGFβ) signaling [103]. EndoMT has been implicated in cancer progression [104]. Therefore, both EMT and EndoMT give rise to cells that have a similar mesenchymal phenotype, and current evidence suggests that both utilize common signaling pathways.

Vascular ECs have been shown to convert to multipotent *Stro1*^+^ MSPCs in a TGFβ or bone morphogenic protein-4 (Bmp4)-dependent manner [105]. Vice versa, MSPCs may also be able to differentiate into ECs [106], but this remains controversial [107] (Figure 1A).

##### (Circulating) Endothelial Cells in MDS and AML

EndoMT seems to be an important source of cancer-associated fibroblasts (CAFs), which are known to facilitate tumor progression in several ways (reviewed in [104]). It was recently shown (for the first time in a hematologic neoplasm) in vitro and in mice that human chronic lymphocytic leukemia-derived exosomes are actively incorporated by MSPCs and ECs, and that this process induces proliferation, migration, and transition to CAFs, as well as angiogenesis [108]. The authors concluded that CAFs exist in (certain types of) leukemia and contribute towards a tumor growth-promoting microenvironment in mice [108]. EndoMT and CAFs have not yet been studied in MDS and AML.

However, it has been well documented that ECs help create a BM microenvironment that augments, protects, and maintains HSC-repopulating capacities in vivo [109,110,111]. Tight spatial co-localization of ECs and perivascular cells [112], as well as E-selectin secretion by ECs [113], has been shown to be critical for maintenance of HSCs, and regulation of HSC proliferation and differentiation. Circulating progenitors of ECs (cPECs) have recently been shown to take part in postnatal angiogenesis [114]. Under the stimulation of growth factors and cytokines, ECs can be mobilized from the BM and reach the sites of neovascularization as circulating ECs (cECs) in both physiological and pathological conditions [115] such as hematologic malignancies [114], including MDS [116,117,118,119,120] and AML [121,122,123,124], where these cells have also been reported to increase in number. In fact, in comparison to healthy controls, cPEC numbers are elevated in patients with low-risk MDS, which display a hypermethylated phenotype, and that exhibit a different gene expression profile. These alterations may result in down-regulation of members of the wingless-Int (*Wnt*) signaling pathway and failure to adequately sustain normal HSCs and normal hematopoiesis, which was particularly prominent with regards to myeloid and megakaryocytic differentiation [120]. MDS-cPECs differed from their normal counterparts in healthy volunteers in both their genetic and epigenetic profiles (i.e., methylation patterns of genes), indicating that the observed endothelial progenitor dysfunctions may be primary in nature [120].

In AML, cEC numbers were significantly higher in patients with rapidly proliferating disease (i.e., elevated white blood cell counts, serum lactate dehydrogenase levels, and circulating blasts) compared to AML patients with lower proliferation rates [123]. Higher levels of cECs in primary human AML samples injected into *NOD/LtSz-scid/IL-2Rγ* null mice coincided with a significantly faster development of leukemia-related symptoms and blast counts in murine blood, BM, and spleen [121]. Of interest, a significant proportion of cECs (20%–78%) in AML patients was shown to bear the same cytogenetic aberrations as the AML clone [122]. Human AML cells themselves can also directly modulate (a) the expansion of ECs (in xenografted mice) [125]; (b) the behavior of resting ECs (in vitro) through the induction of EC proliferation [126]; and (c) the activation and up-regulation of the cell adhesion molecule E-selectin. The latter results in adhesion of a subset of AML cells, which are then sequestered in a quiescent state and become resistant to chemotherapy [127,128]. These AML cells that have become adherent to ECs may later detach and again become proliferative and thus potentially play a role in minimal residual disease and disease relapse [127].

As compared to their counterparts derived from healthy human controls, AML-derived human BM-ECs exhibit similar phenotype and function, but express distinct expression signatures of genes that are thought to be involved in leukemogenesis and NSC maintenance [129]. ECs have also been shown to be capable of inducing chemotherapy resistance in AML cells in vitro, probably via EC-mediated secretion of vascular endothelial growth factor (VEGF) and PDGF [130]. BM-ECs and cECs have thus been proposed as potential adverse prognostic markers and/or therapeutic targets in AML.

As a side note, immunohistochemical and FISH analyses of human B-cell lymphoma samples revealed that a median of 37% of ECs in the microvasculature harbored lymphoma-specific cytogenetic alterations, suggesting that ECs are in part tumor-related or tumor-derived [131].

In addition, up to 56% of ECs generated in vitro from BM-derived progenitor cells of patients with chronic myeloid leukemia carried the disease-defining *BCR/ABL* fusion gene, indicating that certain types of human leukemia may originate from BM-derived hemangioblastic precursor cells [96,101].

### 4.2. Neuro-Ectodermal Origin of MSPC-Like Cells

Until recently, adult MSPCs were considered to be of mesodermal origin [49]. The neural crest is a temporary group of cells that arise from the embryonic ectoderm and give rise to diverse cell lineages, including craniofacial cartilage and bone, smooth muscle, peripheral end enteric neurons, glia, and melanocytes [132].

#### 4.2.1. Pericytes and Endoneural (Myo)fibroblasts as Potential Sources of MSPCs

Pericytes [133,134] were initially thought to be neural-crest derivatives. Pericytes express the pericyte marker NG2 and are contractile cells that wrap around ECs of capillaries and venules [135] where they regulate capillary blood flow. Pericytes are also key components of the neurovascular unit, and as such help sustain the blood-brain barrier [136,137,138]. They communicate with ECs through direct cell-to-cell contact as well as paracrine signaling, and thus stabilize ECs and mediate EC survival and maturation [139]. In addition to their contractile function, pericytes also have phagocytic and immune functions, as well as multipotent ex vivo differentiation capabilities, and they have been termed a “ubiquitous source of adult tissue SCs” [140,141]. As is the case with MSPCs, the markers used to define pericytes are neither specific nor stable in their expression, and there is no single molecular marker that can unequivocally distinguish pericytes from vascular smooth muscle cells or MSPCs [142]. To identify pericytes, additional criteria such as location, morphology, and gene/protein expression pattern are required [142].

Several laboratories proposed a neuro-ectodermal origin of MSPCs from pericytes [39,56,83,143,144,145]. For example, CD146^+^ MSPCs/pericytes were able to establish the human hematopoietic microenvironment in a mouse model [40], suggesting a key role of these cells in the SC niche. In humans, expression of *CD146* has been demonstrated to differentiate between perivascular versus endosteal localization of non-hematopoietic BM-MSPC populations, with CD146^+^CD271^+^ cells localizing in perivascular regions, whereas bone-lining MSPCs expressed *CD271* alone [146]. Several years ago it was speculated that all MSPCs may be pericytes [147]; however, this hypothesis seems unlikely, and the extent of functional and biological overlap between pericytes and MSPCs remains unclear at present (for review see [60]) (Figure 1B).

(Myo)fibroblasts were typically considered to be of mesoderm origin [142]. However, they may also display features of neuro-ectodermal cells, and several groups reported and discussed that MSPCs can also derive from neural crest-derived (endoneural) (myo)fibroblasts [52,56,83,144,148,149] (Figure 1B).

#### 4.2.2. Neural Phenotype Plasticity of MSPCs—Common Neural-Crest Origin with Sympathetic Neurons?

Some populations of multipotent BM-derived human (and murine) MSPCs have distinct receptors that are thought to mark multipotent neural-crest SCs, and thus implicate a neuro-ectodermal origin, the most prominent being CD271 and Nestin.

CD271 (low-affinity nerve growth factor receptor) has been used as a single marker to isolate [150] multipotent [151] MSPCs from humans [58,152,153]. Human CD271^+^ BM-MSPCs were shown to express chemokine (C-X-C motif) ligand 12 (*CXCL12*; in varying degrees) [154,155], but it currently remains unclear whether CD271^+^CXCL12^+^ MSPCs are identical with the CXCL12-abundant reticular (CAR) cell population, or whether they represent a separate reservoir of CXCL12 within the BM, in addition to CAR cells.

Nestin is an intermediate filament protein expressed in neuro-epithelial neuronal precursor SCs, the expression of which decreases with neuronal maturation [156]. However, it must be borne in mind that although Nestin is an important marker for a subset of BM-MSPCs that contribute to HSC maintenance, it is by no means specific for these cells; rather, Nestin^+^ cells are a heterogeneous cell population comprising MSPCs, neural crest-derived SCs (NCSCs) [157,158], ECs [159], endothelial precursor cells [160], myofibroblasts [161], and cancer cells [162]. Nestin^+^ MSPCs were identified as essential components of the endosteal niche [39], and later on, these Nestin^+^ MSPCs were shown to be NCSCs with specialized hematopoietic niche functions, such as secretion of CXCL12 [157]. CXCL12 is the only known chemokine capable of directed migration of HSCs [163], which migrate preferentially to *CXCL12*-expressing niches as was elegantly observed by dynamic in vivo imaging of human AML cells injected into mice [164,165].

BM-derived Nestin^+^ cell populations turned out to be a mixed population of NCSCs, non-myelinating Schwann cell precursors and MSPCs, and both populations had the same ability to truly differentiate into Tuj1-positive cells, with Tuj1 being a neuron-specific marker, indicating that BM-MSPCs share a common origin with sympathetic peripheral neurons and glial cells [157,158,166]. Furthermore, evidence is accumulating that adult human and murine BM-MSPCs are able to differentiate into neuron-like cells (e.g., expressing neuron-specific nuclear protein, neuron-specific enolase, and/or neurofilament-M) and astrocytic-like glial cells (e.g., expressing glial fibrillary acidic protein) under cell culture conditions [167,168,169,170,171,172]. The expression of *Nestin* is lost during this differentiation process [168].

Accumulating data from several groups support the concept that adult Nestin^+^ glial stem/progenitor cells may be the source for NCSCs. In this regard, Nestin^+^-myelinating Schwann cells could be reprogrammed to multipotent NCSCs, thereby gaining the capacity to differentiate into ectodermal, mesodermal, and endodermal cell types in vitro [173,174]. In the presence of Bmp4, these NCSCs follow the mesenchymal differentiation pathway to generate pericyte progenitors and, consequently, MSPCs [174]. The induction of pericytes/MSPCs from NCSCs in culture is thought to reflect the normal fate of NCSCs [142]. During this process, NCSCs lose the neural-crest marker Sox10 and acquire pericyte/MSPC-like characteristics [174], lending further support to the notion that MSPCs originate from perivascular pericytes, which themselves are thought to derive from the neural crest (e.g., [132,134]).

Together these findings suggest that MSPCs may be able to break barriers of germ layer commitment and MSPC plasticity may need to be extended to lineages of neuro-ectodermal origin (e.g., neurons, astrocytes, oligodendrocytes) (Figure 1B). It is worth noting that this conversion can also be induced by epigenetic changes, and by intervention with hypomethylating agents [169,175]. The intriguing role of NCSCs and nervous-system components in the development and regulation of hematopoietic niche homeostasis seems to have been underestimated, as has recently been reviewed by Coste et al. [176], and will be discussed in the context of MDS and AML below.

However, whether the significant amount of (forced) plasticity observed in vitro also exists and is of physiological relevance in humans in vivo (prior to culture) requires further investigation.

##### MSPCs and Neuro-Epithelial Markers in MDS and AML

An intimate spatial relationship between HSPCs and CD271^+^ MSPCs has been described in human BM, with over 80% of the HSPCs being in direct contact with CD271^+^ MSPCs in normal BM, MDS BM, and AML BM [155]. It is worth noting that an increase in CD271^+^ MSPCs in the BM in MDS and AML *versus* normal/reactive BM has been described, with the increase being more pronounced and statistically significant in low-risk MDS compared to high-risk MDS or AML. Perivascular areas, CD146^+^ endothelial areas, and CD163^+^ macrophage areas did not vary across diagnostic categories as compared to normal BM [155]. In addition, this expanded CD271^+^ cell population in MDS showed more widespread expression of *CXCL12* [155]. Others have independently shown that the density of CXCL12^+^ stromal cells that were in direct contact with HSPCs in the BM of patients with MDS or AML with myelodysplasia-related changes was significantly higher than in control BM [177]. Furthermore, CXCL12^+^ cell density correlated negatively with apoptosis in HSPCs, and positively with both BM blast counts and disease progression [177]. It is well established that CXCL12 is indispensable for HSPC homing [178], and it has been shown that isolated HSPCs from MDS patients have impaired CXCL12-directed migratory capacity [179,180]. In addition, endogenous *CXCL12* expression in AML supports autonomous growth of primary human AML cells [165,181]. It has therefore been hypothesized that increased *CXCL12* expression by CD271^+^ MSPCs would attract dysplastic/leukemic blast cells and induce cluster formation and pro-survival signaling. This might explain the prognostically adverse phenomenon of an abnormal localization of immature precursor cells (ALIPs), which could be part of a defective feedback loop between neoplastic HSPCs and altered MSPCs in MDS [155]. Co-culture experiments of normal MSPCs with leukemic blasts demonstrated that the leukemic clone reprogrammed normal MSPCs become AML-MSPCs, i.e., via up-regulation of crosstalk molecules CXCL12 and Jagged1 [182]. These AML-educated MSPCs in turn provided a selective advantage to the leukemic clone, and at the same time suppressed normal primitive hematopoietic cells (lower repopulating capacity of normal HSCs and primitive progenitors) [182]. AML-MSPCs further confer quiescence and chemotherapy resistance to leukemic SCs [182,183]. The unfavorable prognostic significance provides a rationale for the targeting of CXCL12^+^ stromal cells and/or the chemokine (C-X-C motif) receptor 4 (CXCR4)/CXCL12 axis, which has shown promising results in mice [184], and is currently being explored in clinical trials of MDS and AML [185,186].

##### A Potential Role for Sympathetic Nerves in the BM

HSC adhesion and attraction to the SC niche was recently shown to underlie circadian oscillations of the sympathetic nervous system in animal models [187,188] and in humans [189] (reviewed in [190]). Adrenergic signals are locally delivered to stromal cells bearing adrenergic receptors (ARs) by nerves surrounding arterioles in the BM, leading to down-regulation of *CXCL12* and cyclic release of HSCs. The β2 and β3 adrenergic receptors on BM stromal cells cooperate to regulate this process whereby *β2-AR* is expressed on osteoblasts and induces *CLOCK* gene expression, and *β3-AR* is expressed on BM-MSPCs in mice [191] and rats and induces down-regulation of *CXCL12* [192], as well as migration of HSCs away from perivascular units, and induces osteogenic differentiation [188,193]. The *β3-AR* seems to be expressed on primitive MSPCs, such as Nestin^+^ pericytes/MSPCs [39] and CAR cells [194], both of which have been shown to be in immediate contact with nerve fibers and/or neuroglial BM cells, but it is lost during differentiation to osteoblasts [187].

**Figure 2:**
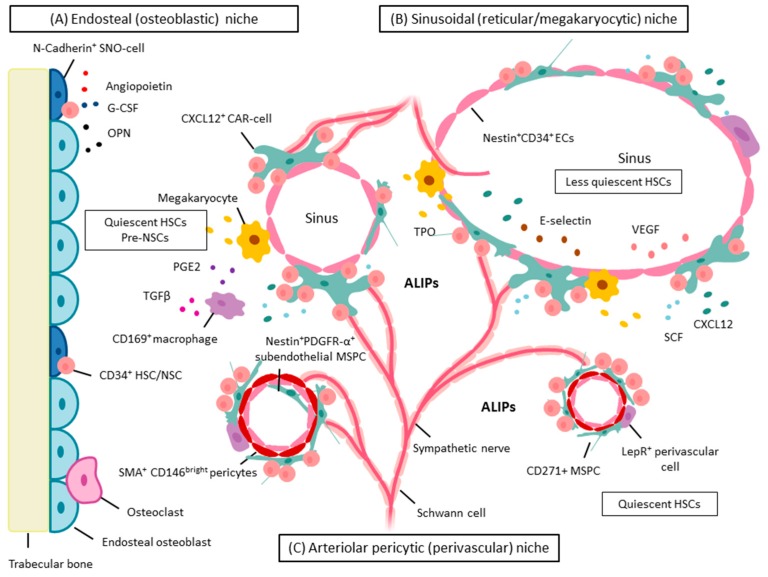
Current stem cell (SC) niche concepts. (**A**) Endosteal (osteoblastic) niche. Hematopoietic stem cell (HSCs) adhere to spindle-shaped N-cadherin^+^ osteoblastic cells. The endosteal niche is thought to promote HSC quiescence. Osteolineage cells provide a specialized niche for early lymphoid progenitors (not shown); (**B**) Sinusoidal (reticular/megakaryocytic) niche. The majority of HSCs are associated with sinusoidal endothelium and/or megakaryocytes, which in turn are intimately associated with sinusoidal endothelium. Leptin-receptor^+^ cells and CXCL12 abundant reticular (CAR) cells are predominantly located in sinusoidal niches and are in close contact with endothelial cells (ECs); (**C**) Arteriolar (perivascular) niche. CD146^+^ pericytes and Leptin-receptor^+^ perivascular stromal cells are the predominant mesenchymal stem and progenitor cell (MSPC) components of the arteriolar niche. Sympathetic nerves are part of the bone marrow (BM) SC niches and are critically involved in the circadian regulation of (i) the secretion of CXCL12 by MSPCs; (ii) HSC adhesion to the SC niche; as well as (iii) osteogenic differentiation. Adrenergic signals and BM neuropathy may also promote leukemic infiltration and disease progression.

The autonomic nervous system is emerging as a “master regulator of hematopoiesis”, and malfunction of this system has been implicated in impaired hematopoiesis (reviewed in [195]). The clinical relevance of the involvement of sympathetic nerve fibers in hematologic diseases has recently been proposed [125,196] and is schematically depicted in Figure 2. BM neuroglial damage coincided with reduced sympathetic nerve fibers in the BM and was shown to compromise function, survival, and number of MSPCs and to critically contribute to the pathogenesis of myeloproliferative neoplasms [196]. By contrast, no studies have examined the possible impact of the autonomic nervous system in MDS. However, recently, adrenergic signals and sympathetic neuropathy have also been shown to promote leukemic BM infiltration in a murine AML xenograft model, in which primary human AML cells were transplanted into denervated and control *NOD*-scid *IL2Rγc^−/−^* mice [125]. In this model, development of AML resulted in a significant reduction of arterioles innerved by catecholaminergic fibers compared to healthy controls, and those arterioles that remained innervated exhibited significant reductions in the density of surrounding nerve fibers [125]. This AML-induced neuropathy was characterized by sympathetic denervation of BM arterioles and locally reduced sympathetic tone, and was shown to reinforce leukemia progression through transformation of the HSC niche. The latter was associated with (a) depletion of arteriole-associated pericytic NG2^+^ niche cells that maintain healthy HSCs; and (b) expansion of leukemia-supportive, more differentiated Nestin^+^Leptin-receptor^+^ mesenchymal progenitors [125].

#### 4.2.3. EMT as Potential Source of MSPCs

Generation of MSPCs from the neural crest likely occurs via an EMT. EMT is an embryonic process that becomes latent in most normal adult tissues. However, it has been shown that induction of EMT can give rise to cells that have similar gene expression profiles, a similar multi-lineage differentiation capacity, and a similar ability to migrate towards tumor cells and wound sites when compared to MSPCs [197]. Loss of E-cadherin is fundamental to EMT. Several EMT-inducing transcription factors can repress E-cadherin directly or indirectly (e.g., Snail, Slug, and TWIST), and EMT can be induced via several signaling pathways (e.g., TGFβ, Wnt/β-catenenin, and Notch). The EMT-inducer TWIST is required for mesenchymal differentiation from the neural crest, and has consequently been proposed as an indicator of the neuro-ectodermal origin of MSPCs; TWIST is not only a master transcriptional regulator for embryogenesis, cell lineage determination, and development of mesenchymal cell lineages, but can induce expression of markers of MSPC stemness such as *Stro1*, and plays an important role in MSPC self-renewal, maintenance, and differentiation. Furthermore, TWIST has been shown to play a key functional and developmental role in normal and malignant hematopoiesis. *TWIST* is highly expressed in murine and human HSCs and is involved in the regulation of HSC self-renewal and myeloid lineage development [198,199]. TWIST is also thought to play a critical role in the generation of cancer SCs [200,201].

##### TWIST in MDS and AML

In adult humans, *TWIST* is mainly expressed in hematopoietic precursor cells maintaining their undifferentiated state, with expression decreasing with differentiation [202]. EMT, EMT-inducing transcription factors, and EMT-associated signaling pathways have been associated with cancer. For example, over-expression of *TWIST* has been associated with aggressive phenotypes, disease progression, poor prognosis, protection from chemotherapy and apoptosis, and EMT and metastasis formation in various solid tumors [203]. The role of *TWIST* in human hematologic malignancies is less well studied [199,202,204].

Recently, significant up-regulation of *TWIST* was shown in CD34^+^ hematopoietic (up-regulation) BM cells from patients with MDS [205]. *TWIST* expression was modified by stroma contact and significantly correlated with advanced disease stage and control of p53-mediated apoptosis in CD34^+^ cells derived from MDS BM, but not in CD34^+^ cells from healthy subjects [205,206]. TWIST has also been shown to function as a tumor suppressor in AML via direct regulation and activation of the tumor-suppressor gene *p21*. Similar to observations made in other leukemias [207,208], this pathway was epigenetically silenced by hypermethylation in 31% of adult AML patients, which provided leukemic cells with a proliferation and survival advantage [209]. Other studies have demonstrated an over-expression of *TWIST* in BM mononuclear cells of patients with AML [210,211], and this correlated with a more aggressive clinical phenotype [210], and shorter overall and event-free survival in multivariate analysis [211], thus identifying *TWIST* as a novel candidate gene contributing to leukemogenesis.

The above data provide evidence for a role of *TWIST* in the pathophysiology of clonal myeloid diseases, including MDS and AML, and provide a rationale for targeting *TWIST* or components of the signaling pathway in the future [202,205,209,211,212]. In this regard, in vitro re-expression of *TWIST* occurred after treatment with hypomethylating agents [207], which have emerged as the gold standard of treatment for elderly MDS and AML patients. In addition, pathways related to EMT-like processes have also been implicated in hematological malignancies, and can identify specific AML subtypes [213].

### 4.3. MSPCs: Evidence for Multiple Developmental Origins

Today MSPCs (and pericytes) are considered to develop from multiple distinct developmental origins and progenitor cells [83,89,142,196]. While pericytes and (myo)fibroblasts were initially thought to be mesoderm derivatives, it is now clear that they have several different developmental origins, including the mesoderm and the neural crest (reviewed in [142]). Embryonic sources of pericytes include neuro-ectoderm-derived neural crest cells that are thought to give rise to pericytes in the brain and central nervous system (Figure 1B), whereas pericytes in other organs have been postulated to originate from the mesoderm-derived MSPCs (Figure 1A). Postnatal sources of pericytes include mesoderm-derived circulating BM-MSPCs, transdifferentiated ECs, CD146^+^ EPCs, mesothelial cells that have undergone EMT, or as yet undetermined “other sources” [47,138,142,214,215] (reviewed by Hill et al. [136]). Similarly, the source of (myo)fibroblasts remains controversial, and they have been reported to develop from epithelial cells via EMT, from ECs via EndoMT, from BM-MSPCs, from peripheral blood pluripotent circulating progenitor cells, and from pericytes (recently reviewed in [216,217]).

Although most of the cells derived from MSCs are mesodermal cells, some of them seem to belong to the neural crest that is of ectoderm origin [89]. In fact, multi-potential stemness of MSCs with differentiation potentials for all MSC-related cell lineages (bone, muscle, tendon, fat, and marrow stroma) has not yet been satisfactorily demonstrated in humans in vivo (even though this is a definition criterion of MSCs).

This hypothesis was recently underscored by the observation in mice that slowly proliferating neural crest-derived MSPCs (Nestin^+^PDGFRα) share a common origin with sympathetic peripheral neurons and glial cells, differentiate into SC niche-forming MSPCs, and conserve MSPC activity throughout life, whereas mesoderm-derived Nestin-MSPCs participate in fetal skeletogenesis and lose MSPC activity soon after birth.

Summarizing the above, an evolving concept, similar to that of HSCs, is to view MSPCs as a heterogeneous mixture of subpopulations, which may derive from differing developmental origins, each harboring a unique set of multipotency [89].

## 5. BM Microenvironment and Stem Cell Niche Concepts

The BM microenvironment is formed by various cellular components and a plethora of soluble factors that these cells produce. Cellular components of the BM microenvironment include stromal cells and accessory cells (Table 1) [31,218,219,220,221,222,223,224,225,226,227,228,229,230,231,232,233,234,235,236,237]. Stromal cells include MSPCs, fibroblasts, sinusoidal ECs [218,227,228], adipocytes [233], osteoclasts, and BM macrophages [221,222,223,224,234]. Accessory cells comprise myeloid regulatory cells (circulating macrophages, DCs, and myeloid-derived suppressor cells) and lymphoid regulatory cells (T-regulatory cells (Tregs) [225], NKCs, and B-cells) (Table 1, left column).

HSCs reside in a highly complex and dynamic microenvironment [31], which is referred to as the SC niche, a concept that was already proposed as early as 1978 [238]. The SC niche is thought to be a hierarchically organized regulatory unit consisting of several stromal constituents that maintains and directs HSC self-renewal and differentiation [60].

According to the SC niche concept, cellular components of the BM microenvironment can also be grouped according to their involvement in the SC niche into “niche cells” and “niche accessory cells” (reviewed in [60]) (Table 1, right column). HSCs are tethered to cellular niche constituents by cellular adhesion molecules and their receptors (e.g., vascular cell adhesion molecule/very late antigen-4 (VLA-4), intracellular adhesion molecule/lymphocyte function-associated antigen-1 (LFA-1), E-selectin/CD44, CXCL12/CXCR4) [239].

The heterogeneity of stromal cells that comprise the SC niche and the complexity of the signals they generate have been extensively reviewed by others [31,244,245]; HSCs may occupy multiple niches in diverse tissues such as the BM and spleen [227]. Within the BM microenvironment a minority of HSCs are in close contact with, and are supported by, a population of bone surface-lining osteoblasts called spindle-shaped N-cadherin^+^ osteoblastic (SNO) cells [242], giving rise to the concept of “**endosteal niches**” (Figure 2A). The endosteal niche has also been termed “**osteoblastic niche**” [246,247] or, more recently, “**osteo-hematopoietic niche**” [18], and is thought to promote HSC quiescence. The majority of HSCs, however, seem to be associated with sinusoidal endothelium in the BM, giving rise to the concept of “**vascular sinusoidal niches**”, sometimes also termed “**sinusoidal reticular niches**” (Figure 2B) [227,240,242,245]. Further refinements were introduced by the identification of SMA^+^CD146^bright^ pericytes in humans [40,144,146,155], an observation that was further developed and gave rise to the model of the “**perivascular arteriolar niche**” or “**pericytic arteriolar niche**” (Figure 2C). In humans, HSPCs also show predominant perivascular distribution [155]. Later on, CAR cells, Nestin^+^ cells, Leptin-receptor^+^ cells, and pericytes were added to the picture in continuous efforts to more fully understand the spatial and functional relationships of the BM microenvironment [248]. CXCL12^+^ CAR cells are in close contact with ECs and HSCs, and are predominantly located in sinusoidal areas or niches, as are Leptin-receptor^+^ cells [249]. In mice, HSCs and early myeloid progenitors are predominantly found in the perivascular niche, whereas early lymphoid progenitors occupy the endosteal niche, where “osteo-lineage” cells are thought to provide a specialized niche for lymphoid progenitors [240], with CAR cells being required for the proliferation of both lymphoid and erythroid progenitors [250]. About one year ago, it was discovered that approximately 20% of HSPCs localize directly adjacent to megakaryocytes, which in turn are intimately associated with BM sinusoidal endothelium in mice, and have been shown to be critically involved in HSC maintenance and quiescence through CXCL4 secretion [251,252,253,254]. Thus the terminus “**sinusoidal megakaryocytic niche**” entered the scene (Figure 2B). It currently remains unclear whether all of the above are truly distinct niches [31] that provide either synchronous or redundant regulation of HSCs, or whether these niches provide unique regulatory functions [241]. What is clear, however, is that the SC niche is not physiologically static but responds to microenvironmental stimuli and is under constant remodeling, which also underlies a circadian rhythm [31,187,189,230,245].

### 5.1. Dysplastic and Leukemic Niches

As mentioned above, the BM microenvironment is severely disrupted in MDS, and MDS cells are heavily dependent on their “**dysplastic niche**”. Most cells of the BM microenvironment are also pathologically altered in MDS, including ECs [120], osteoblasts [255,256,257], macrophages [258], and various immune cells such as NKCs [259,260,261], NKTs [262,263], Tregs [264,265,266], T-helper (Th) 1, 2, 17 and Th22 cells [264,265,267,268], CD8^+^ CTLs [269], DCs [270,271,272], and myeloid-derived suppressor cells (MDSCs) [273]. The plethora of alterations observed in MSPCs in MDS will be discussed in detail below. Suffice to state here that MDS-MSPCs have reduced capacity to support normal hematopoiesis, paralleled by enhanced supportive capacities for clonal hematopoiesis in vitro (e.g., [33,274,275]) and in vivo in murine xenograft settings [40,276,277,278,279,280,281,282].

Similar to observations made in MDS, most cellular constituents of the BM microenvironment in AML are pathologically altered, including ECs [129,130], osteoblasts [23], fibroblasts [283], and various immune cells such as NKCs [259,284,285,286], NKTs [259,287], Tregs [288,289], Th-subsets [290,291,292,293], DCs [272,294] and MDSCs [295]. These aberrant cells contribute to the “**leukemic niche**”, either as niche constituents, or “niche accessory cells”. The contribution of immune cells to the leukemic niche and their involvement in regulating the leukemic clone has been discussed by others [16].

BM stromal populations have been implicated in MDS and AML pathogenesis, as has been reviewed by others [16,17,18,31,241,296,297,298].

#### NSCs: Competition for the Stem Cell Niche—Spatial Localization in Mice

Colmone et al. elegantly applied dynamic in vivo imaging techniques to show that AML cells create a pathologic microenvironment that disrupts and usurps normal HSC niches that sequester human transplanted HSCs and HPSCs, and this is dependent on CXCL12 secretion by the leukemic clone [164]. It has only recently been elucidated that the spatial localization of NSCs within the leukemic niche is restricted to niche elements shared with their non-neoplastic counterparts (in murine transplant experiments) [299,300]. The above data show that NSCs compete with normal HSCs and HPSCs for occupancy of the same protective niche. The SC niche may thus be termed “the home of friend and foe” [301]. Mathematical and in vitro models predict that MDS-initiating cells must have higher self-renewal rates and/or a longer survival (resulting from mutations and local signals from the niche) than normal MSPCs in order to outcompete normal hematopoiesis [302].

### 5.2. Stem Cell Niche: Soluble Components in Normal Hematopoiesis

**Figure 3:**
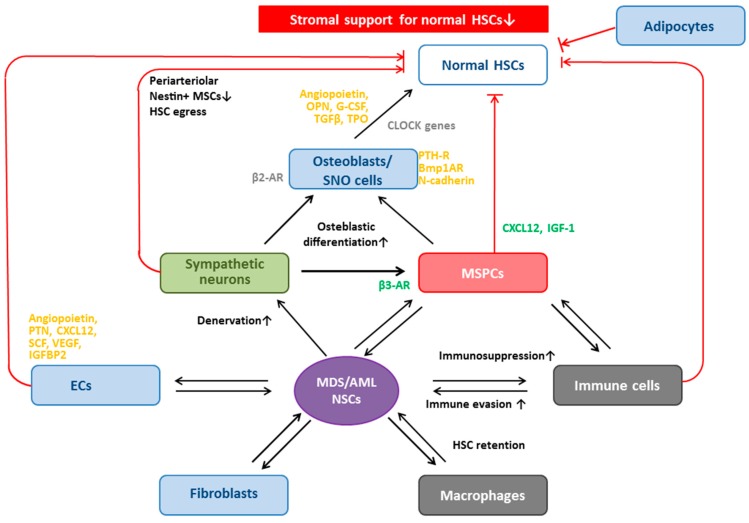
Multidirectional microenvironmental crosstalk in myelodysplastic syndromes (MDS) and acute myeloid leukemia (AML). MDS/AML neoplastic stem cells (NSCs) engage in multidirectional, reciprocal crosstalk with macrophages, fibroblasts, endothelial cells (ECs), sympathetic neurons, and mesenchymal stem and progenitor cells. This creates an environment with impaired stromal support of normal hematopoiesis and preferential stromal and stem cell niche support for NSCs, i.e., a “dysplastic/leukemic niche”, which also provides protection from cytotoxic effects of chemotherapeutic agents. In addition, interaction of NSCs with immune cells results in immunosuppression which fosters immune escape of the clone.

Soluble components relevant to the SC niche include cytokines, chemokines, growth factors, calcium, hormones (parathyroid hormone, estrogen), and hormone-like lipids (prostaglandin E2 (PGE2)). CXCL12, also known as stromal cell-derived factor-1 (SDF1), seems to be the critical chemokine involved in HSC homing and HSC maintenance within the HSC niche [240,303,304,305,306]. It may be produced by MSPCs themselves or by other niche constituents such as MSPC progeny including CAR cells and osteoblasts, as well as by ECs [31,240,303,307]. Other relevant cytokines involved in the fine-tuning of HSC maintenance and regulation include SCF and TGFβ, which can be produced by multiple niche cell types, including but not restricted to osteoblasts and macrophages [112,239,308,309]. In particular, macrophage polarization towards the Type-2 phenotype was recently shown to regulate MSPC osteoblast differentiation in vitro, i.e., via secretion of TGFβ [310]. We have depicted how the secreted products of MSPCs and their progeny work together with other cells of the SC niche to regulate HSC maintenance in Figure 3. This topic has recently been reviewed in detail by others [311].

#### Stem Cell Niche: Soluble Components in MDS and AML

*CXCL12* plays a critical role in SC niche regulation and signals via *CXCR4*, which is highly expressed on human leukemic blasts, mediates homing to protective niches, and regulates pro-survival signals, HSC quiescence, and chemotherapy resistance, all of which ultimately translate into adverse patient outcomes [312,313,314]. Therapeutic targeting of the *CXCL12/CXCR4* axis is under intense investigation [185,315,316,317,318,319].

Other cytokines involved and deregulated in MDS and AML pathogenesis include tumor necrosis factor α (*TNFα*), *SCF, TGFβ, VEGF*, and many others, but will not be discussed here.

### 5.3. Stem Cell Niche: Signaling Pathways in Normal Hematopoiesis

The Wnt [33,279] and Notch [320,321] signaling pathways are likely the main signaling pathways involved in non-neoplastic niche-HSC interactions [322]. Notch/Jagged1 signaling not only governs HSC fate and differentiation [323], but is also involved in MSPC-mediated immunosuppression [324] (discussed in detail in the respective section). It has been demonstrated that normal hematopoiesis is dependent on Notch activation through Notch ligand receptor interactions with non-hematopoietic microenvironmental cells in murine knockout models of lethal myeloproliferative diseases [325].

#### Stem Cell Niche: Signaling Pathways in MDS and AML

Although their exact contribution to disease pathogenesis is still incompletely understood, in vitro results suggest that both Wnt and Notch signaling pathways are pathologically activated in MDS/AML blasts [326], have prognostic relevance [33], and may serve as potential therapeutic targets [327,328,329] in MDS and AML. These signaling pathways have been shown to play a role in MSPC-aided engraftment of human MDS clonal cells in murine xenograft models [140,330].

Notch/Jagged1 signaling has been identified as a critical modulator of niche-based oncogenesis in MDS and AML [321]. Abnormal activation of *Notch* has recently been found in primary human MSPCs and was associated with impaired differentiation and plasticity, thus supporting the concept that primary MSPC defects may contribute to MDS pathogenesis [20,30]. Adding to this, constitutively active *β-catenin* expression, resulting in increased *Jagged1* expression and thus Notch/Jagged1 signaling in osteoblasts, was shown to be critical for the induction of AML in a murine model [23,331].

Similarly, abnormal Wnt/β-catenin signaling in BM-MSPCs from patients with MDS/AML was associated with homing to osteoblastic niche and leukemogenic potency [75], impaired replicative capacity [26,332], adverse karyotypes [333], and/or adverse prognosis [333,334]. Marrow fibrous dysplasia and altered immune responses have also been attributed to β-catenin activation [335]. Antigen-presenting cells (APC) are located on chromosome 5q, deletions of which are common in MDS and AML. They encode a negative regulator of the Wnt signaling pathway with tumor suppressor function. APC^(Min)^ mice with haploinsufficency of APC therefore demonstrated Wnt activation, which coincided with loss of the quiescent HSC fraction and function after serial transplantation, and developed an MDS/myeloproliferative phenotype [336]. The same group also later identified Wnt activation in human AML samples. [75]. It is worth noting that the Wnt pathway is epigenetically regulated in MDS [334,337] and AML [338,339,340,341], and hypomethylating agents have been shown to demethylate *Wnt* antagonist gene promoters in vitro [342]. Despite significant research efforts, the mechanism of action of these drugs is not completely understood [343], and re-expression of *Wnt* may be one of the many reasons why they are clinically effective.

## 6. MSPCs and Their Progeny: Key Cellular Niche Components

MSPCs have been termed the most important cellular “key stone” component of the HSC niche [60]. MSPCs interact with HSCs to regulate HSC self-renewal, differentiation, and, thus, maintenance via adhesion molecule-mediated cell-to-cell interactions, release of cytokines and chemokines [344], as well as provision of regulatory signals (e.g., by expression of crosstalk molecules such as Jagged1 and CXCL12 (Figure 3) [194,240,303,306,345,346]). MSPCs deliver proliferation, stemness, and survival signals to HSPCs, and can also provide protection against cytotoxic effects of chemotherapeutic agents [347].

In vitro experiments have shown that the surface of MSPCs seems to be the predominant site of proliferation of HSCs, whereas the niche-like microenvironment beneath the MSPC layer recruits and retains HSCs with more primitive properties [348]. MSPCs and their immediate progeny—including various perivascular cells such as CAR cells (major source of CXCL12 and SCF) [250], Nestin^+^ perivascular cells, and Leptin-receptor^+^ perivascular stromal cells [31,39,60,112,157,166,227,303,307]—are generally seen as the key regulators of the SC niche. In a broader sense, osteoblasts (source for CXCL12) are also MSPC descendants [219,220,236,349,350,351,352]. The exact sequence of MSPC differentiation is also not clear, but it seems as if Nestin marks more primitive MSPCs with tri-lineage differentiation potential, whereas CXCL12^+^ CAR cells have bi-lineage potential (adipo-osteogenic) (Figure 1A), and Leptin-receptor^+^ MSPCs may represent more differentiated cells.

Perivascular CAR cells express both adipogenic and osteogenic genes and have the potential to differentiate to adipocytes and osteoblasts, rendering them adipo-osteogenic progenitors (Figure 1) [250]. CAR cells have been identified as key components of the SC niche and B-cell niche [194,353,354]. Depletion of CAR cells in genetically engineered mice resulted in significant reduction of HSCs that were not in contact with CAR cells by 50%, paralleled by egress of HSCs to the peripheral blood, as well as induction of early myeloid differentiation at the cost of lymphoid and erythroid progenitors [250]. The authors concluded that CAR cells are essential for the maintenance of HSCs (i.e., keep HSCs in an undifferentiated state) and lymphoid and erythroid progenitors, and also play a role in HSC BM retention [250]. In contrast, HSCs not in contact with CAR cells, e.g., those in the endosteal niche, were not reduced in number [250].

### 6.1. MSPCs: Spatial Localization in Vivo in Normal and Dysplastic Hematopoiesis

Despite intensive research, knowledge on the in vivo HSC niche constituents and their precise role in humans remains limited and must thus be interpreted with caution in the context of human diseases. Van Pel et al. have recently reviewed the comparability of the human and murine SC niche [355]. In 2010, Kunisaki et al. examined the spatial localization of quiescent HSCs in mice, and identified Nestin^bright^ NG2^+^ peri-arteriolar cells of the arteriolar niche to be indispensable for maintaining HSC quiescence, whereas reticular-shaped Nestin^dim^NG2^+^-Leptin-receptor^+^ sinusoidal cells were associated with less quiescent HSCs [249]. Two years later, elegant immunohistochemistry stainings revealed that Nestin^+^ cells also showed predominantly arteriolar distribution in humans [155]. In their seminal work, Flores-Figueroa et al. clarified the relationship of the BM microvasculature in both benign and myelodysplastic human BM. They demonstrated that capillaries and arterioles consist of three layers: a Nestin^+^CD34^+^ EC layer, tightly cloaked by a SMA^+^CD146^bright^ pericyte layer, which is further surrounded by CD271^+^ MSPCs. In thin-walled BM sinusoids, however, the pericyte layer is lacking, and a Nestin^dim^ EC layer directly contacts CD271^+^ MSPCs and hematopoietic elements [155]. Arterioles are therefore structurally distinct from sinusoids in that they are surrounded by layers of smooth muscle cells and pericytes, in addition to being highly innervated by sympathetic nerves that regulate HSC migration [187], and as mentioned above, Nestin^+^ MSPCs are associated with adrenergic neural fibers in vivo (in mice). Occasional subendothelial Nestin^+^ (Sca1^+^PDGFRα^+^) cells have also been observed.

### 6.2. MSPCs: Dichotomous Effects on Erythropoiesis

Although MSPCs are known to support hematopoiesis in general, in vitro evidence is accumulating that MSPCs may specifically inhibit erythropoiesis in favor of myeloid differentiation. Soluble factors produced by BM stromal cells inhibited the ability of HSCs to differentiate into erythroid progenitors while skewing towards myeloid differentiation [36,356,357]. This is thought to be a result of BM stromal cell-secreted interleukin (IL)6, which specifically expanded myeloid progenitors resulting in increased mature myeloid cells in the peripheral blood, but blocked erythroid development [358]. Of interest, elevated levels of the proinflammatory cytokine IL6 (and TNFα) have been correlated with adverse survival in patients with AML [359].

However, conflicting reports dating back as far as 1989 exist, showing that MSPCs can also favor erythroid differentiation through constitutive expression and secretion of activin [360,361,362]. These seemingly contradictory observations were elegantly explained by Gibson et al., who demonstrated that MSPCs initially enhance and then suppress erythroid colony formation [363]. This process was dependent on MSPC density and concentrations of MSPC-secreted PGE [363,364]. In vitro, PGE enhances erythropoiesis, and only seems to be produced by replicating MSPCs when present in low numbers, whereas static (i.e., non-replicating) or confluent (i.e., high cell numbers) MSPC layers produce no PGE and suppress blast forming unit-erythroid (BFU-E) formation [363]. Thus, small numbers of MSPCs enhance erythropoiesis, whereas large numbers of MSPCs inhibit erythropoiesis [365,366].

### 6.3. Osteoblasts Support and Regulate HSCs

Human osteoblasts (well-documented progeny of MSPCs) have been shown to support HSC expansion and to regulate HSC activity in vitro [250], i.e., via secretion of angiopoietin [367], osteopontin (negative regulation of HSC pool size in vivo) [368], and granulocyte colony-stimulating factor (G-CSF) [236,351]. The observed G-CSF secretion may be part of a negative feedback loop, as the application of G-CSF to mice resulted in potent inhibition of osteoblast activity and *CXCL12* expression via indirect mechanisms (as osteoblasts do not express the G-CSF-receptor) [350]. Osteoblasts can also be induced to proliferate and/or foster osteoblast-mediated support of HSC migration and quiescence through interactions of the respective ligands with receptors on the osteoblast cell surface, such as Notch-ligand/N-cadherin and their crosstalk with Wnt/β-catenin signaling, (reviewed, e.g., in [369]) parathyroid hormone (PTH)/PTH-receptor (PTH-R) [219,370,371], and Bmp1A/Bmp1A-receptor [242] interactions. More than a decade ago, SNO cells were identified to be a specialized subpopulation of osteoblasts that line the bone surface. Murine models revealed that they function as key niche components that support and regulate HSC numbers, dependent on signaling via BMP receptor type 1A [242].

#### Osteoblasts in MDS and AML

NSCs were shown to selectively home to and engraft endosteal surfaces in xenograft mouse models [372]. This concept was expanded by Lane et al., who demonstrated that premalignant NSCs (pre-NSCs) and NSCs are biologically distinct in their relative pace of disease onset, leukemogenic potential, and preferential BM homing site: pre-NSCs homed very closely to BM osteoblasts (even closer than normal HSCs) due to constitutive cell-intrinsic Wnt/β-catenin activation, whereas NSCs homed further away from osteolineage cells (in a syngeneic murine model of AML) [75].

Osteocyte-specific deletion of a gene involved in G-CSF production resulted in severe osteopenia and dramatic expansion of myeloid cells [373]. Abnormal osteoblastic signaling via b-catenenin [23,331], Notch-Jagged1 [20], and osteopontin (OPN) [33] has been proposed to be involved in MDS/AML pathogenesis. OPN, also known as bone sialoprotein-1, is implicated in bone remodeling [374], anchors osteoclasts to the mineral matrix of bone [375], plays a role in immunomodulation (as many immune cells express OPN receptors) [376], suppresses HSC proliferation in vitro, and is thought to regulate the SC pool [377]. This molecule also seems relevant in AML, as knockdown of OPN expression induced cell death of AML blasts and leukemic progenitors in vitro [378]. In addition, an immunocompetent model of murine AML revealed reduced levels of osteoprogenitors and OPN^+^ endosteal-lining cells, as well as functionally inhibited osteoblasts with reduced osteocalcin production [379]. This AML-induced uncoupling of osteoblastic and osteoclastic cells is thought to be mediated by leukemic blast-secreted chemokine c-c motif ligand 3 (CCL3) [379]. Finally, high expression levels of OPN in human AML samples at baseline were independently associated with adverse prognosis and survival in multivariate analysis [378,380], underlining a relevant role for this molecule in human AML.

Further details on the presumed roles of osteoprogenitors and osteoblasts in MDS [20,30] and AML [23,257,281] will be discussed below in the context of “Malignancy-Inducing Microenvironment?”.

### 6.4. Other Niche Cells that Regulate HSCs

HSCs are also regulated by other niche constituents, such as ECs (source for CXCL12) (reviewed in [241]), sympathetic neurons [187,188] (major source of CXCL12), and non-myelinating Schwann cells [166] (major source of TGFβ). Evidence is also accumulating that various “niche accessory cells” such as adipocytes (negative regulators of HSCs) [233], osteoclasts [229,231,232], BM macrophages (source of PGE2) [222,237,381], and Tregs [225] act on niche cells to regulate HSCs (Table 1; reviewed by Frenette et al. [60]). Of interest, Frenette et al. showed in elegant murine models that CD169^+^ macrophages promote HSC retention within the BM in vivo, and this process involved crosstalk with Nestin^+^ niche cells, resulting in down-regulation of HSC retention genes, as well as induction of CXCL12 production [221]. Thus, macrophage retention of HSCs antagonizes adrenergic signals, which mediate HSC egress (Figure 3). In addition, the same group demonstrated that CD169^+^ macrophages are involved in the regulation of erythropoiesis [222]. ECs are also relevant regulators of human HSC fate in mice, i.e., via production of pleotrophin (PTN), VEGF, and insulin-like growth factor binding protein, as has been reviewed by Doan et al. [241].

## 7. MSPCs: Immunomodulation

**Figure 4:**
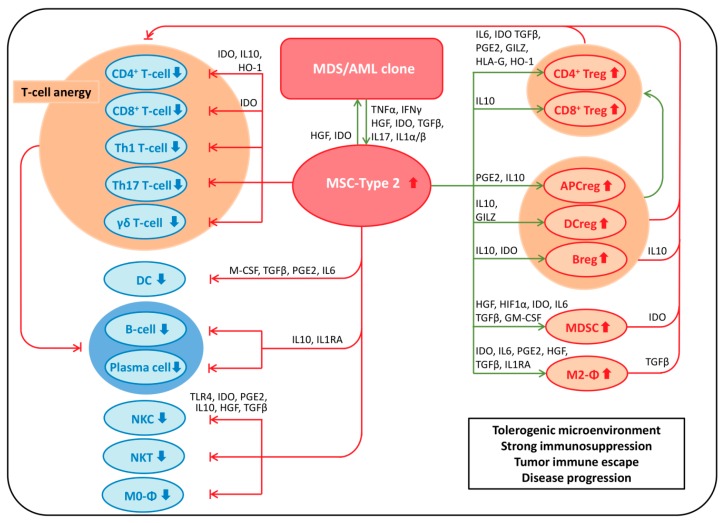
Mechanisms of mesenchymal stem and progenitor cells (MSPC)-mediated immune suppression and evasion. Early-stage myelodysplastic syndromes (MDS) are associated with a state of inflammation. The inflammatory bone marrow (BM) microenvironment is believed to recruit proinflammatory Type-1 MSPCs and license them to adopt a Type-2 immunosuppressing and tumor-promoting phenotype. Together with the leukemic clone, tumor-educated Type-2 MSPCs recruit additional immunosuppressive cells, and suppress those immune cells capable of targeting the leukemic clone, resulting in a strongly immunosuppressive environment, enabling tumor immune escape and disease progression.

In addition to their stem/progenitor cell properties, MSPCs not only provide growth support for HSCs and hematopoiesis [348,382], but also display systemic immunoregulatory and immunosuppressive properties [60,383,384,385,386,387,388] and are capable of influencing both adaptive and innate immune responses (Figure 4) [46]. MSPCs derived from non-BM sources have similar immunosuppressive functions as their BM-derived counterparts [385,389]. Not only MSPCs themselves but also their progeny (i.e., osteoblasts [335,390,391,392], fibroblasts [393,394], and adipocytes [395]) exhibit immunoregulatory properties. While MSPCs are not immune cells themselves, they have been termed “coordinators of the immune system”, emphasizing their key role in modulating immune responses [396]. The exact mechanisms governing the effects of MSPCs on immune cells in humans in vivo have not been fully elucidated, and most published results derive from in vitro and/or murine (xenotransplant) experiments. It must be stressed at this point that rodent and human MSPCs display a number of differences regarding licensing pathways and expression of immune mediators [397], and that in vitro conditions can never fully reflect in vivo conditions due to (a) the considerable level of crosstalk between MSPCs and numerous cells within the microenvironment; and (b) the resulting dynamic alterations of the MSPCs themselves, as well as of the immune cells that they modulate [44]. Therefore, caution needs to be exercised when translating results generated in vitro or in mouse models into the human setting [355,397,398,399,400,401]. Standardization of immune functional assays is hoped to guarantee reproducible and inter-study comparisons of results [63,402].

We give a brief overview of current concepts of how MSPCs interact with immune cells below, with a strong (but not exclusive) focus on human MSPCs (Figure 4).

### 7.1. Immunosuppressive Effects of MSPCs on Immune Cells

In brief, the immunosuppressive capacity of MSPCs observed in vitro or in murine models results from extensive suppression of various effector cells, which is paralleled by a “re-education” of immune cells to become regulatory immune cells with tolerogenic properties (Figure 4) [396]. In this regard, MSPCs have been shown to strongly suppress various proinflammatory immune cells while simultaneously favoring the generation of immunosuppressive immune cell subsets (Table 2).

The current assumption is that MSPC-induced regulatory cells, including Tregs, regulatory B-cells, DCregs, MDSCs and NKregs, gather to create a tolerogenic microenvironment capable of inducing strong immunosuppression (Figure 4) [387,388,461]. However, many key aspects, including potency, specificity, mechanistic basis, and ideally predictable therapeutic modulation of these effects, remain incompletely understood.

#### 7.1.1. MSPCs: Mechanisms of Immunosuppression

##### MSPC-Mediated Immunosuppression via Secretion of Soluble Factors

It is generally accepted that the strong immunosuppressive capacity of MSPCs is mediated via cell-to-cell contact-dependent and independent mechanisms. The latter include various MSPC-secreted soluble (mainly anti-inflammatory) factors, such as hormones, cytokines, and chemokines. These include, e.g., PGE2, TGFβ, IL2, IL6, hepatocyte growth factor (HGF), indoleamine 2,3-dioxygenase (IDO) [399], human leukocyte antigen (HLA), IL 1 receptor antagonist (IL1RA), CCL2, macrophage colony stimulating factor (M-CSF), and MSPC-secreted exosomes as paracrine mediators of MSPC immunosuppressive function (Figure 4) [389,407,409,413,416,425,430,442,450,452,462,463,464,465,466]. The immunosuppressive cytokine IL10 is not produced by MSPCs themselves, but they induce other cell types to do this, in part via heme oxygenase-1 [443,460,467].

Whereas murine MSPC use NO to exert their immunosuppressive function, it is of note that human MSPCs use IDO instead [399]. Under anti-inflammatory licensing conditions, MSPCs can be converted to Type-2 cells that secrete high levels of IDO, IL6, IL27, TGFβ, PGE2, monocyte chemotactic protein 1 (MCP1), and intracellular adhesion molecule 1 [468,469,470]. The highly immunosuppressive enzyme IDO is not only secreted by MSPCs, but also by several components of the BM microenvironment, including MDSCs [471,472] and DCs [473,474]. IDO serves as a molecular switch towards immune-suppression and facilitates escape from immune recognition by several means: (a) direct induction of the emergence of Tregs [412]; (b) inhibition of the activation of pro-inflammatory monocytes and macrophages [475]; and (c) induction of the switch from M0- to M2-macrophages (Φ) [46,403,461,476,477]. The latter exert an anti-inflammatory effect by secreting high levels of IL10 and TGFβ, resulting in further polarization towards Treg induction (Figure 4) [478].

MSPCs perpetuate and contribute to the maintenance of the anti-inflammatory, immunosuppressive microenvironment via modulation of various types of immune cells: (a) direct and indirect strong suppression of activation and proliferation of CD4^+^ T-helper (Th) cells (Th1, Th2, and Th17), as well as cytotoxic CD8*^+^* T-cells [384,404,479,480,481]; (b) inhibition of the differentiation of monocytes into mature DCs [425]; (c) direct suppression of DC differentiation, maturation, and function [406,449,482,483,484], which results in altered cytokine expression, inhibition of the endocytotic capacity with impaired antigen presentation [485], and consecutive additional inhibition of T-cell activation and proliferation; (d) induction of differentiation of DCs towards an MDSC phenotype, which act as immune suppressors [458]; (e) numerical expansion of MDSCs, i.e., via secretion of HGF [457]; (f) powerful inhibition of NKC proliferation and functions via expression of toll-like receptor (TLR)4, PGE2, and IDO [417,457,486]; and (g) inhibition of B-cell activation [432] and antibody production, as well as alteration of naïve and memory B-cell subsets [430,432,486] (Table 2).

Due to the limited diffusion range of the soluble factors secreted by MSPCs, close proximity of MSPCs to the immune cells is required, and MSPC-secreted chemokines have been suggested to play a role in attracting immune cells [463,465]. In addition, interferon (IFN)γ priming combined with TLR activation renders MSPCs capable of recruiting immune inflammatory cells [487]. Vice versa, the cellular and soluble microenvironment surrounding MSPCs, in particular various immune cells and their secreted cytokines, may also critically influence the immunomodulatory function and plasticity of MSPCs [488].

##### MSPC-Mediated Immunosuppression via Expression of Cell Surface Molecules

Another means by which MSPCs keep immune cells at a close distance is via direct binding of, e.g., CD4^+^ and CD8^+^ T-cells to the cell surface of MSPCs, as has been demonstrated in vitro [489,490]. MSPCs also exert metabolic control over the immune system, e.g., via strong constitutive expression of the cell surface molecule CD73, which is involved in elimination of the inflammation-promoting effect of ATP, via metabolization of ATP to adenosine [491]. MSPCs can also be induced to secrete the enzyme IDO, which potently inhibits lymphocyte proliferation by metabolizing L-tryptophan to L-kynurenine [492]. Tryptophan starvation results in T-cell cycle arrest [492], and kynurenine and its metabolites are also directly toxic for T-cells [493] and NKCs [494], and can also inhibit production of erythropoietin [26]. Heme oxygenase-1 is involved in porphyrin metabolism, and was identified as a key contributor for MSPC-mediated suppression via induction of Tregs and promotion of MSPC-produced IL10 [443]. MSPCs also express cell surface molecules with immunosuppressive capacity, such as programmed death ligand 1 (PD-L1) and Fas ligand, on their cell surface, which enables them to directly deliver inhibitory signals to immune cells expressing *PD-L1* and/or *Fas* via cell-to-cell contact mechanisms [495,496]. In this regard, it was recently demonstrated that MSPCs repress Th1 and Th17 polarization [409,410], i.e., via up-regulation/constitutive expression of *PD-L1* on MSPCs [411]. The immunosuppressive effect of MSPC-expressed *PD-L1* on T-cells could be abolished by PD-L1 antibodies in vitro [496].

### 7.2. MSPC-Mediated Mechanisms of Immune Evasion in Malignancy

Inflammation is a well-established key component of the malignant microenvironment, and it plays a relevant role in tumorigenesis and tumor progression [497]. Tumors and their microenvironment induce MSPC homing, i.e., via secretion of cytokines, chemokines, and/or growth factors [498]. MSPCs that home to the tumor site are then thought to be modulated by the inflammatory tumor microenvironment to become activated and switch their phenotype from MSPC Type-1 cells (pro-inflammatory or naïve MSPCs) to “tumor-educated” MSPC Type-2 cells (anti-inflammatory or tumor MSPCs) (Figure 4) [498]. Alternatively, mouse models have shown that the tumor-attracted MSPCs may also differentiate into CAFs in the case of BM origin [499], or into vascular ECs in the case of adipose tissue-derived MSPCs [500], but this remains to be demonstrated in vivo in humans. As discussed above, MSPCs found at malignant sites are significantly affected by both the inflammatory microenvironment as well as the cancer cells themselves. Whereas naïve (innate) MSPCs can inhibit the proliferation of tumor cell lines of hematopoietic origin [501,502,503], tumor educated Type-2 MPSCs exhibit stronger immunosuppressive and migratory properties, have strong drug resistance, induce EMT, promote tumor cell proliferation, and increase the proportion of cancer SCs (reviewed in [498]). Evidence is mounting that tumor-educated MSPCs share immunosuppressive functions with their BM counterparts (reviewed in [504]). In comparison to normal MSPCs, tumor-educated Type-2 MSPCs more strongly recruit monocytes/macrophages [505], increase the proportion of Tregs [506], and inhibit the cytotoxic effects of (antigen-specific) T-cells [507,508,509], B-cells [509,510], and NKCs [511,512] as compared to their normal counterparts, respectively, thereby creating favorable conditions for tumor progression [513]. In addition, tryptophan metabolites may be mutagenic [514,515], and could thus contribute to genetic instability. MSPC-secreted IDO and PGE2 have been implicated to play a relevant role in this process in vitro and in vivo in humanized murine models [509,512]. In addition, many human tumors have been shown to produce IDO [516,517,518]. Cancer, the inflammatory cancer microenvironment, and tumor-educated Type-2 MSPCs thus form a self-reinforcing vicious cycle [498].

### 7.3. MSPC-Mediated Mechanisms of Immune Evasion in MDS and AML

It is thought that the above-described tumor-induced MSPC-mediated recruitment of various immune cells and modulation towards an anti-inflammatory, immunosuppressive environment also occurs in MDS and AML, fostering immune escape of the dysplastic/leukemic clone in general, and of NSCs in particular. As discussed above, MSPCs can be reprogrammed by the MDS/AML clone, which can be measured, i.e., by abnormal secretion of various cytokines and other soluble factors. These imbalances may contribute to altered trafficking of immune cells and contribute to the immune evasive capacities of MDS/AML-MSPCs [33,79].

Lower (absent) expression levels of co-stimulatory molecules (CD40, CD80, CD86) on MSPCs of patients with MDS or AML [483] and higher levels of immunosuppressive cytokines (TGFβ, IL6, HGF) may explain the altered immunosuppressive capacity of MDS/AML-MSPCs [483,519]. This may include suppression of T-cells and DCs, and concomitant recruitment, retention, activation, and differentiation induction of immunosuppressive cell types such as Tregs, MDSCs, and Type-2-Φ (Figure 4). It is worth noting that the immunosuppressive capacity of MSPCs seems impaired in (low-risk) MDS [483,520,521], and is significantly lower in low-risk MDS than in high-risk MDS [79,519], which is in line with the increased Treg number and activity in high-risk MDS [265], and a continuous immune subversion occurring during disease progression from low-risk MDS to high-risk MDS to AML, as discussed above.

MSPCs obtained from patients with MDS have been shown to secrete the strongly immunosuppressive enzyme IDO [509]. Co-culturing of MDS-MSPCs with healthy DCs in vitro resulted in inhibition of DC differentiation and maturation from monocytes, reduced expression of T-cell co-stimulatory molecules (CD80, CD83, CD86), and inhibition of T-cell proliferation (Figure 4) [519].

In addition, IDO can also be produced by AML blasts as an immune subversion strategy (Figure 4) [522,523]. IDO expressed by primary human AML samples results in induction of Treg differentiation, as well as impaired maturation of DCs and reduced naïve T-cell proliferation [524,525], and has been shown to be critically involved in AML-induced immune tolerance in vitro [523]. Constitutive over-expression of IDO has been observed in AML blast cells and patient sera [522,523,526], correlates with increased levels of circulating Tregs in patients with AML at initial diagnosis [524], and has been linked with poor clinical outcome (decreased relapse-free and overall survival) in patients with AML [527]. Elevated levels of IDO metabolites have also been observed in MDS patients, and correlated with cytopenias in these patients [528]. In vitro experiments suggest that tryptophan catabolites contribute to cytopenias via significant inhibition of HSPC expansion [528], and possibly also via inhibition of erythropoietin production [26].

### 7.4. MSPCs: Immunosuppressive Licensing

MSPCs adapt their immunoregulatory properties to their local microenvironment. The immunosuppressive capacity of human MSPCs is not intrinsic, but requires activation or “licensing” from an inflammatory microenvironment by inflammatory factors (such as IFNγ, TNFα, IL1α/β, and/or IL17) and is also dependent on bidirectional interactions with immune cells mediated by direct cell contact [399,443,529,530,531,532]. A pro-inflammatory microenvironment, in particular pro-inflammatory cytokines, seems to direct MSPCs to the target site [533] and promote the up-regulation of anti-inflammatory molecules such as IDO [532], as well as the cell surface expression of adhesion molecules on MSPCs that allows their close interaction with immune cells, contributing to increased efficiency of paracrine mediators with immunosuppressive function produced by MSPCs [470]. Some pro-inflammatory factors (e.g., IL6) may also be up-regulated under inflammatory licensing conditions but are far outweighed by the immunosuppressive effects of MSPCs [396]. The interplay of various complex stimuli involved in the intricate balance of MSPC licensing has been concisely reviewed by Krampera [534].

### 7.5. MSPCs: TLR Signaling Balances Immunosuppressive versus Pro-Inflammatory Licensing

The notion that MSPCs can only be licensed towards an immunosuppressive phenotype was challenged by Waterman et al. [535]. They describe that MSPCs can be polarized or “licensed” towards either a pro-inflammatory (Type-1 MSPCs) or an anti-inflammatory (Type-2 MSPCs) phenotype, depending on the type of activated TLR, ligand concentration, timing, and kinetics of activation [487,535]. Signaling via TLRs and acute IFNα/γ and TNFα production are critically involved in sensing the increased demand for myeloid cells in “danger situations” such as infections, where “emergency myelopoiesis” is required. Chronic excessive IFN and TNF production, however, results in HSC exhaustion (reviewed in [16]). Human BM-derived MSPCs express high levels of functionally active TLR3 and TLR4 [536]. *TLR4*-priming polarizes towards pro-inflammatory Type-1 MSPCs with the capacity to present antigens, release pro-inflammatory cytokines and chemokines capable of recruiting other inflammatory immune cells [487], and activate B-cells [537] and T-cells in vitro [535]. In addition, TLR4 signaling is considered to mediate the capacity of MSPCs to support the proliferation and differentiation of HSCs and HSPCs [538], and may also be involved in MSPC autophagy and phagocytosis [539,540,541]. Interestingly, TLR4 also plays a crucial role in MSPC-mediated inhibition of NKC function [542]. In contrast, TLR3 priming polarizes towards anti-inflammatory Type-2 MSPCs with the capacity to inhibit lymphocyte proliferation in vitro (via up-regulation of anti-inflammatory IL4, IDO, or PGE2) [535]. In line with most of the above, Type-1 MSPCs attenuate, whereas Type-2 MSPCs promote, tumor growth [543], and these paracrine effects may be mediated by MSPC-secreted exosomes [544].

MSPCs have been shown to gain the capacity to process and present exogenous antigens via both MHC-I and MHC-II in certain inflammatory microenvironments (e.g., during a narrow window of low levels of IFNγ, before IFNγ levels are increased, and at low MSPC cell density), and can thus induce adaptive CD4^+^ and CD8^+^ T-cell responses, respectively [401,489,545,546,547]. They have thus been termed “conditional antigen-presenting cells” [545]. This may be in line with Waterman’s proposed licensing towards Type-1 MSPCs [535,543]. Furthermore, priming of human MSPCs with inflammatory cytokines has been shown to force MSPCs to acquire IDO-mediated efficient antimicrobial activities against pathogens including bacteria, viruses, and protozoan parasites [468]. Therefore, under certain microenvironmental conditions, MSPCs might be envisioned to enhance immune cell activity, and even result in the induction of a tumor-antigen-specific immune response, but this topic will not be reviewed here.

However, contradictory in vitro results with human BM-MSPCs exist, claiming that ligation with either TLR3 or TLR4 induces MSPC migration, pro-inflammatory signals and inhibits the capacity of MSPCs to suppress T-cell proliferation by significant down-regulation of Notch ligand Jagged1, but without influencing IDO activity or PGE2 levels [536,548], and yet others have reported that ligation of TLR3 and TLR4 on human BM-MSPCs resulted in enhanced immunosuppression and induction of IDO [549]. Adding to this, in vivo effects reported in mouse models of several diseases seem to be in apparent contradiction to the polarizing process described by Waterman et al. in vitro, and thus the in vivo modulation of MSPCs by TLRs requires further clarification [550,551]. Possibly, it may be time to reassess the Type-1/2 MSPC paradigm, as has recently been done for the M1/M2 macrophage paradigm [552]. These authors believe that macrophages do not form stable stereotype subsets, but respond to a combination of multiple factors in their systemic and local micro-milieu present in vivo, with several pathways interacting and converging to form complex, even mixed, phenotypes [537]. In our opinion, the same may be true for MSPCs, and in order to gain a more dynamic view of these complex processes and to understand the full functional range of differentially activated or “licensed” MSPCs, considerably more information will be required about MSPCs in vivo. In this regard, it has been proposed that BM-MSPCs may represent a fulcrum that initially orchestrates an inflammatory response (to eliminate the target), which is sequentially followed by immunosuppression later on (to preserve host integrity), and that this intricate balance in dichotomous BM-MSPC functions (“plasticity”) is linked to TLR signaling and complex immune crosstalk [553]. The plasticity of MSPCs in immune modulation has been reviewed by others [488].

#### TLR Signaling in MDS and AML

TLRs encode key innate immune signal initiators and participate in the fine-tuning of the inflammatory immune response as well as in the regulation of hematopoiesis, i.e., via induction of the expression of certain miRNAs [554]. Murine data suggest that TLR signaling regulates both hematopoiesis (by promoting myeloid differentiation) and HSC self-renewal in a cell autonomous fashion [555,556,557]. The participation of the innate immune system and abnormally activated TLR signaling in the pathogenesis of MDS has been well documented and multiple genes known to be regulated by TLRs were found to be over-expressed in MDS [558]. Activated TLR signaling is thought to be involved in the excessive levels of apoptosis and the accompanying cytopenia(s) observed in early-stage MDS. In this regard, *TLR1*, *2*, and *6* expression was recently reported to be higher in CD34^+^ blasts from patients with lower-risk MDS than in higher-risk MDS or controls [559] whereas *TLR2* and *4* expression did not differ between AML patients and healthy volunteers [560]. Increased levels of TLR2 [559] and/or TLR4 [561] were positively correlated with an increased rate of apoptosis, and as proof of principle with TLR2 activation-induced apoptosis in vitro. TLR2 activation has also been implicated in the inhibition of erythropoiesis [554,558,562]. In addition, a recurrent genetic variant of TLR2 resulting in enhanced activation of downstream signaling was found in 11% of MDS patients [558]. Deregulated TLR expression has varied effects on the survival of MDS patients and requires further clarification. For example, higher expression of *TLR2* [558] seemed to correlate with prolonged survival, whereas higher expression of *TLR6* [558], *TLR7* [554], and *MYD88* [562] (a key mediator of TLR signaling) had a tendency to negatively correlate with survival in patients with MDS. Larger patient cohorts are needed to confirm these initial observations. Preliminary in vitro observations indicate that antagonization of aberrant TLR signaling has been proposed as a therapeutic goal in MDS [554,558,562].

In AML, the situation may be the inverse: stimulation of the same receptors, namely TLR2 and 4, resulted in induction of immune escape mechanisms such as up-regulation of *PD-L1*, which protected AML cells from cytotoxic T lymphocyte (CTL)-mediated lysis in vitro [563]. Deregulated TLR signaling has also been implicated as a potential prognostic marker and therapeutic target in AML. In AML, the application of TLR agonists for induction of maturationof blast-derived DCs (for immunotherapy approaches aimed at inducing tumor-specific CTLs) is being assessed in vitro [563,564,565,566,567,568] and has started to enter clinical trials [569]. Recent evidence also implicates direct anti-leukemic effects for TLR8 activation independent of its immunomodulating properties [570].

### 7.6. MSPCs: Autophagy and Phagocytosis

Autophagosomes are present in MSPCs at a level higher than in many differentiated cells, but are arrested in mid-autophagy (i.e., autophagosomes are not fused with lysosomes and are not degraded) while being maintained as multipotent cells, whereas autophagy was activated during differentiation of MSPCs [571]. In addition, autophagy is essential for HSC maintenance [572] and it has been suggested that autophagy may be a hallmark of a SC [571]. The molecular machinery of autophagy orchestrates protective responses to danger stimuli such as cell starvation, infection, autoimmunity, and cancer [571,573]. Apart from organismal development and intimate involvement in the innate immune response, autophagy also participates in tumor suppression [573]. As a consequence, defects in autophagic machinery can cause or contribute to cancer initiation and/or progression [573,574].

Autophagy and phagocytosis mechanistically overlap [575], and TLR(4) signaling is involved in both processes by providing an inductive signal [539,540,541]. It has been reported that MSPCs may have the capacity to phagocytose apoptotic cells [576] but further evidence is lacking.

#### Autophagy in MDS and AML

Elegant murine experiments have recently shown that the loss of key autophagy genes *Atg5* or *Atg7* in HSCs or HSPCs leads to a lethal pre-leukemic phenotype in mice, and heterozygous loss of autophagy in a mouse model of AML resulted in more aggressive disease, implicating defects in autophagy in the pathogenesis of MDS and the progression to AML [572,574,577]. It is worth noting that autophagy gene losses are found within chromosomal regions that are commonly deleted in human AML, and reduced expression of autophagy genes was found in human AML blasts [574]. Functionally, aberrant Wnt/β*-*catenin and/or phosphoinositide 3-kinase (PI3K)/protein kinase B (Akt)/mechanistic target of rapamycin signaling, frequently implicated in the pathogenesis of AML, has also been shown to suppress autophagy, resulting in low differentiation ability [578,579]. In fact, functional autophagy may be required to prevent AML progression from MDS [577]. In line with this, several groups have demonstrated that autophagy levels [580,581] or autophagy-associated genes [582,583] were significantly increased in BM mononuclear cells from patients with lower-risk MDS, but not in higher-risk MDS or AML compared with control patients. It has been suggested that autophagy might be considered as a cell protective mechanism in lower-risk MDS, and that defective autophagy in higher-risk MDS might contribute to disease progression [580]. As such, therapeutic induction of autophagy may provide a unique approach to targeting primitive leukemic precursors in MDS [584,585,586] and AML [587,588,589]. In this regard, down-regulation of autophagy-associated genes was shown to be due to promoter hypermethylation in higher-risk MDS and AML, which is potentially targetable with hypomethylating agents. As proof of principle, re-expression of autophagy-associated genes as well as ensuing apoptosis induction has been observed in AML cell lines [582].

## 8. MSPCs in MDS and AML: Aberrant Function

Early disease-initiating events and the cell of origin in MDS and AML remain poorly defined and are an area of intense research efforts. However, the involvement of an altered dysplastic or “leukemic niche” with instructive or at least disease-permissive functions seems evident. MSPCs from patients across all MDS subtypes and AML show aberrant biological properties in most reports (Table 3), but conflicting data exist [590,591]. MDS-MSPCs have been shown to be structurally, epigenetically and functionally altered, which leads to impaired stromal support and seems to contribute to deficient hematopoiesis [592]. The plethora of intrinsic functional aberrations include starkly reduced growth, proliferation and differentiation potential, accompanied by premature replicative senescence and impaired supportive capacity for normal hematopoiesis in long-term cell culture assays (Table 3).

During disease progression it seems as if MDS-MSPCs acquire or regain proliferative and differentiation potential. In this respect, increased MSPC cell density has been observed in the BM of higher-grade MDS compared with lower-grade MDS and benign hematologic diseases, and was shown to independently correlate with significantly shorter overall survival [81]. In comparison, an overall shift in MSPC differentiation also takes place in AML where an elevated frequency of mature osteoblastic cells, coinciding with significant reduction of primitive MSPC-subsets and lower numbers of fibroblast colony-forming units (CFU-Fs), was observed in humans [182]. It is worth noting that the presence of high numbers of primitive Nestin^+^ MSPCs and CFU-Fs in the BM of AML patients at baseline predicted early relapse after treatment, whereas large numbers of more mature MSPCs or osteoblastic cells were significantly associated with more delayed late relapse [182]. These data indicate that Nestin^+^ MSPCs play a dominant role in maintenance and self-renewal of NSCs responsible for AML relapse.

### 8.1. Dysplastic/Leukemic Niche: Altered Gene Expression

In BM-MSPCs from MDS patients, altered expression levels of several chemokines as well as of gene sets associated with differentiation, fibrosis, adhesion, extracellular matrix remodeling, and key molecules involved in the crosstalk with HSCs and HSPCs, including up-regulation of Notch ligand Jagged1, were reported [33,279]. Primary MDS-MSPCs secrete high levels of TGFβ, which is thought to contribute to reduced supportive capacity for normal HSCs, as well as erythroid impairment [356,604]. The TGFβ pathway is also constitutively activated in CD34^+^ BM precursors of MDS patients [604]. Suppression of TGFβ signaling stimulated hematopoiesis in vitro in BM aspirates of MDS patients, and in vivo in a murine model of BM failure [604].

In AML-MSPCs the situation regarding TGFβ signaling may be inhibited due to the up-regulation of the transcription factor FOS, a known inhibitor of TGFβ signaling [324]. The authors hypothesized that this may represent a (defective) negative feedback loop [324]. In addition, MSPCs isolated from primary human AML BM samples demonstrated significantly reduced MCP1 levels, and a trend towards lower IL6 and granulocyte macrophage colony-stimulating factor levels, consistent with the view that the leukemic BM microenvironment has a diminished capacity to support normal SCs and hematopoiesis [33,324]. Highly specific differences in methylation patterns of genes were found between MSPCs from healthy volunteers and patients with MDS [33] or AML [592], indicating differential activity and function of MSPCs in these diseases.

Existing data needs to be reconfirmed before firm conclusions can be drawn, and it must be noted that MSPCs alter their transcriptome and phenotype depending on culture conditions. Therefore, results obtained in vitro must be regarded with scrutiny.

### 8.2. NSCs: Bidirectional Crosstalk with MSPCs—“Reprogramming” to Diseased MSPCs

It is commonly accepted that MSPCs are involved in the formation of the supportive structure and safe haven in which MDS/AML blasts reside and proliferate, and are heavily involved in the pathophysiology of MDS and AML. It has very recently been demonstrated that cytokine-mediated crosstalk [605], exosome-mediated protein transfer and RNA trafficking [606,607,608,609], as well as cell-to-cell contact mechanisms [182] between primary AML cells and MSPCs, alter the phenotype, transcriptome, and function of normal MSPCs.

Chemokine release by tumor cells has been shown to attract MSPCs [610]. In this regard, primary AML blasts isolated from patients also exhibit a broad constitutive chemokine release profile in vitro [611], which may attract MSPCs to the leukemic niche. CCL3, for example, is frequently increased in primary human AML samples [612] and murine data suggest involvement in leukemic blast-induced changes in the BM microenvironment [603]. Critical signaling pathways underlying the crosstalk between NSCs and the BM microenvironment have been reviewed elsewhere [613].

Elegant co-culture experiments have shown that conditioned medium from AML cell lines [592] and human MDS cells [279] can “reprogram” healthy human MSPCs to adopt MDS/AML-MSPC-like molecular features. This included profound transcriptional changes, including down-regulation of cell cycle-promoting genes, which is paralleled by prominent up-regulation of cytokine/inflammation-related genes and various crosstalk molecules. This functionally translated into reduced supportive capacity for non-malignant HSCs, and was molecularly reflected by specific methylation signatures [592]. Others have similarly shown that leukemic myeloid cells stimulate MSPCs to alter their gene expression profile, resulting in progressive remodeling of the endosteal BM niche into a disease-permissive leukemic niche with severely compromised ability to maintain normal HSCs, thus favoring and effectively supporting NSCs [603].

Recently, Medyouf et al. succeeded in creating a humanized model of MDS in mice. They showed that co-transplantation of CD34^+^ BM samples from MDS patients with their corresponding in vitro expanded MSPCs (from the same patients) results in sufficient engraftment. They also found that significantly enhanced engraftment rates were produced when autologous MSPCs were used compared to co-transplantation with healthy MSPCs, demonstrating that MDS-MSPCs differ from healthy MSPCs at the functional level in that they specifically support dysplastic cells [279]. Expression of the cell surface protein CD146 (which is involved in adhesion, migration, and mesenchymal differentiation) on CXCL12 co-expressing pericytes seems to be important for the MSPC-aided in vitro propagation [40,614,615] and/or in vivo engraftment of clonal MDS cells [276]. These and other data indicate that dysplastic/leukemic blasts can cleverly cause alterations of MSPCs and force them to adapt to their command, culminating in a microenvironment that is permissive for preferential support of NSCs, i.e., a protective “dysplastic/leukemic niche” [164,182,300,379,616,617,618]. As a consequence, impaired stromal support of normal hematopoiesis results in peripheral cytopenia(s) typically occurring in MDS and AML. These data implicate MSPCs in MDS disease initiation and progression [296]. Several groups concluded that neoplastic blasts seem to have an instructive role on MSPC functionality, and that remodeling of the mesenchymal niche by neoplastic blasts represents an intrinsic self-reinforcing process of leukemogenesis [279,592,603]. Bidirectional crosstalk between NSCs and MSPCs not only provides leukemic blasts with pro-survival benefits [619], but also protects the malignant clone from NKC-mediated lysis [620], promotes quiescence and chemotherapy resistance of NSCs, and may possibly predict clinical post-treatment outcomes in patients with AML [182].

The exact mechanisms underlying the various phases of NSC homing into the niche and the various interactions between NSCs and the niche once these cells have completed the homing process have not yet been elucidated. We do not know if (or by what mechanism) NSCs gain clonal and/or functional dominance over normal HSCs first, before kicking them out of their protective niche and then reprogramming the niche into a “hostel for the hostile”, or if, vice versa, NSCs start to boss MSPCs and other niche constituents around “from outside” first, and then enter the niche once it is already transformed to a “leukemic niche”.

It is of clinical relevance that the reprogramming of MSPCs by the malignant clone may occur via epigenetic changes, highlighting a trigger that is amenable to therapeutic targeting, and may also explain why hypomethylating agents are effective in delaying disease progression [296]. Initial data that azacitidine may normalize the structure and function of aberrant human HSCs and MSPCs in vitro have been presented, but not yet fully published [621,622,623].

### 8.3. NSCs: Bidirectional Crosstalk with ECs

As discussed above, alterations in (circulating) ECs of patients with MDS and AML are thought to contribute to disease perpetuation. Human AML cells can also directly attach to and modulate the expansion and activation of ECs (in mice and in vitro), suggesting that ECs and leukemia cells can dramatically impact one another [125,126,127,128]. The critical role of ECs in the BM vascular niche and bidirectional crosstalk between vascular ECs and HSCs (or NSCs) in the dynamic regulation of the niche have been reviewed by Colmone et al. [624]. According to this model, AML cells significantly change the behavior of ECs to produce microenvironments permissive to AML growth, opening new avenues for AML therapy to include agents that prevent EC activation (e.g., anti-TNF-α antibodies, antioxidants, or glucocorticoids), with the aim of reducing NSC adhesion to the protective niche, thereby potentially increasing sensitivity to conventional chemotherapy [127].

### 8.4. NSCs: Bidirectional Crosstalk with Other Cells

As mentioned above, human AML cells can also induce transformation of the SC niche (in mice and humans) through sympathetic denervation of BM arterioles, which results in loss of HSC-maintaining pericytic niche cells, and promotes dominance of the neoplastic clone over normal HSCs [125]. In addition, functional abnormalities and alterations in gene expression have also been observed in fibroblasts and macrophages derived from the BM of AML patients [283].

## 9. MSPCs in MDS and AML: Clone-Derived or Clone-Induced?

It remains controversially debated whether MSPCs represent an intrinsically abnormal stromal compartment, or if these cells are derived from the MDS/AML clone [29,591,596,597,599,625,626,627,628,629,630,631,632,633,634,635,636,637]. Neoplastic cells may remain dormant and clinically unapparent for decades. This switch from quiescent dormancy to overt malignancy is thought to be induced by acquisition of (additional) mutations in neoplastic cells and/or the surrounding/supporting stromal cells [638].

Asymmetric aneuploidy has been found to be prevalent in malignant hematologic diseases, but it remains unclear whether these aneuploid MSPC clones represent senescent or transformed cells [639]. MSPCs from patients with acute lymphoblastic leukemia demonstrated leukemia-associated chromosomal translocations in 10 of 10 patients’ samples analyzed, with proportions of translocation-positive MSPCs varying from 10% to 54% [640]. Several groups have shown cytogenetic aberrations in MSPCs derived and cultured from patients with MDS [66,590,593,598] or AML [597,598], but these chromosomal lesions were distinct from those found in the MDS/AML clone, and of uncertain pathophysiologic relevance. Thus, MSPCs from MDS and AML patients do not appear to carry the same cytogenetic alterations as the malignant clone, which argues against a common origin of these cells. However, as mentioned above, MSPCs exhibit chromosomal abnormalities in up to 64% of MDS patients and up to 54% of AML patients, suggesting enhanced genetic instability of MSPCs in these diseases [597,600,601]. Alternatively, the earliest cells involved in leukemogenesis did not express any of these lesions, but did acquire them after diversification into hematopoietic and non-hematopoietic sub-clones. The concept of a common origin has recently also been supported by the observation that chromosomal aberrations mirroring those found in the corresponding leukemic blasts (in addition to distinct additional cytogenetic aberrations) were detected in AML-MSPCs in some AML patients [324]. Discrepant findings in the literature regarding the clonality of MSPCs may result from a lack of standardized methods for the screening of chromosomal abnormalities of MSPCs as well as the lack of harmonization of MSPC-purifying strategies, but also from the varying culture periods, different numbers of passages, and different tissue culture media used to grow cells prior to cytogenetic analyses [324,639,641].

Spontaneous formation of hybrids between cancer cells and normal human BM-derived MSPCs has been observed in solid tumors, and they have been advocated as tumor progression mechanisms [642,643,644]. Martin-Padura et al. showed for the first time that spontaneous fusion of human AML cells with macrophages, DCs, and ECs occurs in vivo in mice, resulting in gene transfer with leukemic potential remaining in these hybrid cells [645]. It therefore seems interesting to contemplate the possibility that the formation of hybrids between AML-cells and MSPCs may also be possible, and that this mechanism might explain observed cytogenetic changes in MDS/AML-MSPCs, but this hypothesis remains highly speculative.

Cytogenetic aberrations in MSPCs may therefore be due to several controversially discussed possibilities. In cases where the clonal aberrations are the same as those observed in the MDS/AML clone, MSPCs may be part of the leukemic clone or take up chromosomal material from malignant cells in the form of exosomes or the formation of tumor-cell hybrids. For cases where the clonal aberrations observed in MSPCs are not present in the malignant clone, this might reflect either a generally enhanced chromosomal instability in the patient or involvement of an extremely immature (multipotent) progenitor that had the ability to give rise to both MSPCs and NSCs in the malignant process (in analogy to observations of SC plasticity in chronic myeloid leukemia [96,101]).

## 10. Malignancy-Inducing Microenvironment?

The behavior of tumors and the spectrum of disease is much more diverse in vivo in humans than in vitro, and this is thought to be due to the contribution of the microenvironment in general, and stromal cells in particular [638]. The importance of mutations within tumor stroma has been shown by several groups [638,646,647,648], but it remains controversial whether the malignant clone dictates the aberrant behavior of the stroma, or whether the stroma induces malignancy. Initially it was thought that transformed cells recruit, influence, and direct tumor-stromal cell interactions. Houghton et al. present an alternate view: that stromal cells may initiate (and/or unmask latent) transformation to overt malignancy. They elegantly addressed the question of whether mutated stromal cells can promote tumors by injecting p53-mutated MSPCs into mice with a predisposition to breast cancer (Apc^(Min/+)^). An increased incidence of breast cancer in mice carrying the Apc^(Min/+)^ mutation was observed, whereas p53 mutation-bearing MSPCs could be recovered up to a year later in wild-type mice without affecting their health [638]. Similar observations were made for hematologic neoplasms. Walkley et al. showed that mice with a deficiency in the retinoic acid receptor and deletion of the retinoblastoma gene develop myeloproliferation, even when transplanted with wild-type BM [649], indicating that the disease arose due to alterations in the BM microenvironment (in this case retinoic acid receptor deficiency) [649]. Others have demonstrated that the development of a myeloproliferative disease could be induced by inactivation of Mind Bomb-1, which, as an essential component for Notch ligand endocytosis, resulted in defective Notch activation in the non-hematopoietic microenvironment [325].

In parallel, murine model data emerged, showing how directed mutations of stromal cells can result in myelodysplasia and induction of AML [23,281]. These data demonstrate that genetic alterations in MSPCs can foster the outgrowth of leukemic clones (recently reviewed in [618]). Stromal abnormalities occurring in MDS/AML are thought to contribute to functional alterations and increased cellular apoptosis, resulting in disease progression and a malignancy-inducing/fostering microenvironment. Deletion of *dicer-1* and/or *sbds* (the gene mutated in the human BM-failure and leukemia pre-disposing condition Schwachman-Bodian-Diamond syndrome) in murine osteoprogenitors resulted in disruption of hematopoiesis and induction of myelodysplasia and AML [281]. Osteocyte-specific deletion of a signaling molecule involved in G-CSF secretion resulted in induction of myeloproliferation in mice [373]. Similarly, it was demonstrated that activating *β-catenin* mutations in mouse osteoblasts, resulting in enhanced expression of the Notch-ligand Jagged1, induced AML in mice [23]. In this regard, abnormal activation of the Notch signaling pathway was recently demonstrated in primary human BM-MSPCs from patients with MDS [20,30]. Elegant murine xenograft experiments have demonstrated that co-injection of human (CD146^+^) BM stromal cells with hematopoietic MDS cells into mice enabled propagation and survival of the MDS clone [277,279,282].

Taken together, the above data strongly indicate that aberrations in the BM microenvironment can be disease-initiating events that transform HSCs and HSPCs into abnormal cells in MDS and AML, a concept that has been developed using murine disease models. Bearing all the inherent limitations of mouse models of human diseases in mind, however, direct transfer of these findings into the human clinical setting remains speculative. Nevertheless, relevant progress has been made in that mouse models of MDS and AML can now be genetically engineered and faithfully recapitulate human disease, and they have recently been acknowledged to be useful research tools [650].

In addition, primary abnormalities of human endothelial progenitor cells were recently shown in patients with low-risk MDS, and co-culture experiments with CD34^+^ cells implicated primary dysfunctions of the vascular niche as important drivers for myelodysplasia [120].

Of interest, HSC aging is epigenetically regulated and has been proposed to produce an environment that is conductive to myeloid malignancies such as MDS and AML (recently reviewed in [651]), which are typically prevalent in the elderly (median age 77 years in real-life cohorts) [5,652,653]. Epigenetic changes in HSC regulation are potentially reversible, and have thus been proposed to be therapeutically targetable, e.g., with hypomethylating agents.

Several groups tentatively concluded that primary stromal dysfunction can contribute to the evolution of secondary neoplastic disease, supporting the concept of niche-induced myelodysplasia and/or leukemia [20,23,26,30,281,282].

## 11. Conclusions

We have summarized current concepts on the relevance of MSPCs in hematopoiesis in general, and MDS and AML in particular. Although the exact role of these cells in normal BM and in MDS or AML remains uncertain, we believe that these cells may be critically involved in the pathogenesis of MDS and AML. As dual enablers of the SC niche and regulators of immune response, MSPCs have also been purported to “sit at the nexus of cancer SC biology and cancer immunology” [60]. In this sense, the acronym “MSCs” could also be interpreted as “Masters of Survival and Clonality”.

As discussed above, there is now evidence of several disease-perpetuating crosstalk interactions between “diseased” or “reprogrammed” MSPCs (and their progeny) and NSCs, ECs, sympathetic nerves, and various immune cells, as well as between NSCs and MSPCs, ECs, macrophages, sympathetic nerves, and various immune cells, respectively. All of these crosstalk pathways culminate in complex multidirectional crosstalk patterns and feedback loops (Figure 4), which are utilized by malignant blasts to create a permissive microenvironment that attenuates normal HSC growth and function, remodels the microenvironment (fibrosis, loss of adrenergic nerves), promotes angiogenesis, facilitates mutagenesis, and enables immune escape, ultimately promoting disease progression. Thus, several tiles have been added to the complex mosaic of MDS/AML pathogenesis, showing that primary defects of MSPCs, endothelial progenitors, osteoprogenitors, and osteoblasts can be drivers of MDS and/or AML. Dysplastic and leukemic blasts can cause alterations of MSPCs, and force them to adapt to their command, culminating in a “reprogrammed” microenvironment that is permissive for preferential support of NSCs.

In the last few years, as immunological knowledge and more detailed knowledge of the composition and function of the SC niche in health and disease are increasing exponentially, acceptance has dawned that the ultimate goal of a cure in MDS and AML, with eradication of persistent and drug-resistant NSCs, will very likely not be achieved through selective targeting of the dysplastic/leukemic clone (alone). Rather, a “three-pronged approach” of combined targeting of (a) the malignant clone itself; (b) immune cells, with the aim of inducting or resurrecting an adequate antitumor immune response; and (c) the surrounding microenvironmental protective niche to render it less hospitable to malignant cells and more amenable to normal HSCs will be needed. With regard to the latter, niche displacement of human NSCs via cytokine-induced mobilization of established leukemia from the BM has been shown to allow their replacement with healthy HSPCs (in mice) [299]. Targeting of the BM microenvironment in general [182,654,655], and of MSPCs as trailblazing constituents of the dysplastic/leukemic niche in particular, is an extremely relevant topic. A number of promising agents that target the interactions of the MDS/AML clone with MSPCs are currently in various phases of clinical testing (e.g., hypomethylating agents, lenalidomide, TGFβ-superfamily ligand traps, and adoptive MSPC transfer–based approaches). The detailed discussion of this interesting topic is, however, beyond the scope of this review and will be discussed elsewhere. Furthermore, the crucial role of peripheral innervation and neural signaling in regulating hematopoietic hemostasis has only recently emerged, and therapeutic strategies to target this previously unrecognized stromal constituent will likely emerge [656].

The interesting concept that quantitative and qualitative BM stroma composition may be exploited as a potential clinical biomarker to predict response or relapse should be further evaluated in clinical trial settings. If this proves true, individual microenvironment composition at initial diagnosis and also during or after therapy may well guide therapeutic planning and open a new horizon of personalized medicine in the future.

As our understanding of the cellular composition of the normal, dysplastic, and leukemic niches and the complex interactions between these cells is constantly evolving, conclusions drawn from the current data landscape may need to be built upon and/or adapted in the coming years.

## Figures and Tables

**Table 1:** Cellular components of the BM microenvironment [31,60,155,218,219,220,221,222,223,224,225,226,227,228,229,230,231,232,233,234,235,236,237,240,241,242,243].

Grouping A	Grouping B
**Stromal Cells**	**Niche Cells**
**Mesenchymal stem cells**	**Mesenchymal stem cells**
**Fibroblasts**	**Endothelial cells**
**Endothelial cells**	**Osteoblastic cells**
**Adipocytes**	**Sympathetic neurons**
**Tissue macrophages**	Non-myelinating Schwann cells
Osteoclasts	**Perivascular stromal cells**
Bone marrow macrophages	CXCL12-abundant reticular cells
	Nestin^+^ perivascular cells
	Leptin-receptor^+^ perivascular cells
**Accessory Cells**	**Niche Accessory Cells**
**Myeloid regulatory cells**	**Adipocytes**
Circulating macrophages	**Osteoclastic cells**
Dendritic cells	**Bone marrow macrophages**
Myeloid-derived suppressor cells	**T-regulatory cells**
**Lymphoid regulatory cells**	**Megakaryocytes**
T-regulatory cells	
Natural killer cells	
B-cells	

**Table 2:** MSPC-mediated modulation of the immune response.

**MSPCs Suppress Inflammatory Immune Cell Subsets**	**References**
CD4*^+^* and CD8^+^ T-cell proliferation and activation	[403,404,405,406,407,408,409]
Th1 and Th17 conversion	[410,411,412,413]
γδ T-cell proliferation and possibly cytotoxicity	[344,414,415,416]
NKT cell proliferation	[416]
NKC proliferation, cytokine production and cytotoxicity, and decreased expression of activating receptors	[408,417,418,419,420]
DC maturation, proliferation, differentiation, and pro-inflammatory function, and/or antigen-presenting capacity, with ensuing inability to prime T-cells	[421,422,423,424,425,426,427,428]
B-cell proliferation, differentiation, and function (in part through the modulation of T-cell help by MSPCs)	[408,413,429,430,431,432,433,434]
Plasma cell formation	[413,433,435]
Inflammatory potential of activated neutrophils	[436]
**MSPCs Favor Immunosuppressive Regulatory Cell Subsets**	
CD4^+^ Tregs	[410,418,437,438,439,440,441,442,443,444,445]
CD8^+^ Tregs	[444,446]
Invariant NK regulatory cells (NKregs)	[180,416]
Regulatory DCs (DCregs) with T-cell suppressive properties, induction of T-cell anergy, and the capacity to induce Tregs	[388,447,448,449]
Polarization of macrophages towards the anti-inflammatory M2-phenotype with T-cell-suppressive properties	[413,422,450,451,452,453,454]
Regulatory antigen-presenting cells (APCregs) with T-cell-suppressive properties	[455,456]
MDSCs	[457,458]
B regulatory cells	[433,435,459,460]

**Table 3:** Human MSPC alterations in MDS and AML.

Human MSPC/Stromal Cell Alterations	Disease	References
Impaired proliferative and clonogenic potential in cell passages, growth and differentiation defects, altered morphology, disrupted clonal architecture (less CFU, less cobble-stone area formation)	MDS, AML	[20,25,26,30,33,66,68,274,279,592,593,594,595]
Higher apoptotic index	MDS	[32]
Increased senescence	MDS	[468]
Increased density of primitive MSPCs (CD271^+^ or Nestin^+^)	MDS, AML	[81,182]
Reduced support for HSCs and/or hematopoiesis	MDS, AML	[25,33,34,35,80,274,592]
Chromosomal abnormalities	MDS, AML	[66,324,593,596,597,598,599,600,601,602]
Epigenetic changes (altered methylation profile)	MDS	[33]
Altered adhesion molecule profiles	MDS	[68,274]
Altered levels of cytokine or chemokine production	MDS, AML	[32,33,274,324,592,603]
Deregulated signaling (Wnt/β-catenin, Notch/Jagged1, KIT/SCF, senescence-associated CDKN1A/2A/2B)	MDS, AML	[26,30,31,33,332]
DNA methylation changes	MDS, AML	[33,592]

## Abbreviations

αSMA	α-smooth muscle actin
Akt	protein kinase B
ALIPs	abnormal localization of immature precursors
AML	acute myeloid leukemia
APC	antigen-presenting cells
APCregs	regulatory antigen-presenting cells
BFU-E	blast forming unit-erythroid
BM	bone marrow
Bmp4	bone morphogenic protein
CAFs	cancer-associated fibroblasts
CAR	CXCL12-abundant reticular
CCL	chemokine (c-c motif) ligand
CECs	circulating ECs
CFU-F	fibroblast colony-forming unit
cPEC	circulating progenitors of ECs
CXCL12	chemokine (C-X-C motif) ligand 12
CXCR4	chemokine (C-X-C motif) receptor 4
DC	dendritic cell
EC	endothelial cell
EMT	epithelial-to-mesenchymal transition
EndoMT	endothelial-to-mesenchymal transition
G-CSF	granulocyte colony-stimulating factor
HGF	hepatocyte growth factor
HLA	human leukocyte antigen
HLA-DR	human leukocyte antigen-D-related
HSC	hematopoietic stem cell
HSPC	hematopoietic stem/progenitor cell
IDO	indoleamine 2,3-dioxygenase
IFN	interferon
IL	interleukin
IL1RA	interleukin 1 receptor antagonist
ISCT	International Society for Cellular Therapy
LFA-1	lymphocyte function-associated antigen-1
LPM	lateral plate mesoderm
LSC	leukemic stem cell
MCP1	monocyte chemotactic protein 1
M-CSF	macrophage colony-stimulating factor
MDS	myelodysplastic syndromes
MDSC	myeloid-derived suppressor cells
MSC	mesenchymal stem cell
MSPC	mesenchymal stem and progenitor cells
NCSC	neural crest-derived stem cell
NKC	natural killer cell
NKT	natural killer T-cell
NSC	neoplastic stem cell
OPN	osteopontin
PDGFRα	platelet-derived growth factor-α
PD-L1	programmed death ligand 1
PGE2	prostaglandin E2
PI3K	phosphoinositide 3-kinase
PTH	parathyroid hormone
PTH-R	parathyroid hormone receptor
PTN	pleotrophin
SC	stem cells
SCF	stem-cell factor
SDF1	stromal cell-derived factor-1
SNO	spindle-shaped N-cadherin^+^ osteoblastic
SSEA-4	stage-specific embryonic antigen-4
TGFβ	tumor growth factor β
TH	T-helper
TLR	toll-like receptor
TNFα	tumor necrosis factor α
Tregs	regulatory T-cells
VEGF	vascular endothelial growth factor
VLA-4	very late antigen-4
Wnt	wingless-Int
Φ	macrophage

## References

[1] Bryder D., Rossi D.J., Weissman I.L. (2006). Hematopoietic stem cells: The paradigmatic tissue-specific stem cell. Am. J. Pathol..

[2] Wognum A.W., Szilvassy S.J. Hematopoietic stem and progenitor cells. http://www.stemcell.com/~/media/Technical%20Resources/F/B/7/E/9/MR019HematopoiesisOnline_29784WEB.pdf.

[3] Pleyer L., Neureiter D., Faber V., Greil R., Greil R., Pleyer L., Neureiter D., Faber V. (2010). Myelodysplastic syndromes (MDS). Chronic Myeloid Neoplasias and Clonal Overlap Syndromes: Epidemiology, Pathophysiology and Treatment Options.

[4] Ma X., Does M., Raza A., Mayne S.T. (2007). Myelodysplastic syndromes: Incidence and survival in the United States. Cancer.

[5] Pleyer L., Burgstaller S., Girschikofsky M., Linkesch W., Stauder R., Pfeilstocker M., Schreder M., Tinchon C., Sliwa T., Lang A. (2014). Azacitidine in 302 patients with WHO-defined acute myeloid leukemia: Results from the Austrian Azacitidine Registry of the AGMT-Study Group. Ann. Hematol..

[6] Pleyer L., Burgstaller S., Stauder R., Girschikofsky M., Sill H., Schlick K., Thaler J., Halter B., Machherndl-Spandl S., Zebisch A. (2016). Azacitidine front-line in 339 patients with myelodysplastic syndromes and acute myeloid leukaemia: Comparison of French-American-British and World Health Organization classifications. J. Hematol. Oncol..

[7] Hope K.J., Jin L., Dick J.E. (2004). Acute myeloid leukemia originates from a hierarchy of leukemic stem cell classes that differ in self-renewal capacity. Nat. Immunol..

[8] Passegue E., Jamieson C.H., Ailles L.E., Weissman I.L. (2003). Normal and leukemic hematopoiesis: Are leukemias a stem cell disorder or a reacquisition of stem cell characteristics?. PNAS.

[9] Jung N., Dai B., Gentles A.J., Majeti R., Feinberg A.P. (2015). An LSC epigenetic signature is largely mutation independent and implicates the HOXA cluster in AML pathogenesis. Nat. Commun..

[10] Sarry J.E., Murphy K., Perry R., Sanchez P.V., Secreto A., Keefer C., Swider C.R., Strzelecki A.C., Cavelier C., Recher C. (2011). Human acute myelogenous leukemia stem cells are rare and heterogeneous when assayed in NOD/SCID/IL2Rgammac-deficient mice. J. Clin. Investig..

[11] Pang W.W., Pluvinage J.V., Price E.A., Sridhar K., Arber D.A., Greenberg P.L., Schrier S.L., Park C.Y., Weissman I.L. (2013). Hematopoietic stem cell and progenitor cell mechanisms in myelodysplastic syndromes. Proc. Natl. Acad. Sci. USA.

[12] Dayyani F., Conley A.P., Strom S.S., Stevenson W., Cortes J.E., Borthakur G., Faderl S., O’Brien S., Pierce S., Kantarjian H. (2010). Cause of death in patients with lower-risk myelodysplastic syndrome. Cancer.

[13] Chang H.Y., Rodriguez V., Narboni G., Bodey G.P., Luna M.A., Freireich E.J. (1976). Causes of death in adults with acute leukemia. Medicine.

[14] Schumacher T.N., Schreiber R.D. (2015). Neoantigens in cancer immunotherapy. Science.

[15] Zitvogel L., Apetoh L., Ghiringhelli F., André F., Tesniere A., Kroemer G. (2008). The anticancer immune response: Indispensable for therapeutic success?. J. Clin. Investig..

[16] Riether C., Schurch C.M., Ochsenbein A.F. (2015). Regulation of hematopoietic and leukemic stem cells by the immune system. Cell Death Differ..

[17] Balderman S.R., Calvi L.M. (2014). Biology of BM failure syndromes: Role of microenvironment and niches. Hematol. Am. Soc. Hematol. Educ. Program..

[18] Bulycheva E., Rauner M., Medyouf H., Theurl I., Bornhauser M., Hofbauer L.C., Platzbecker U. (2015). Myelodysplasia is in the niche: Novel concepts and emerging therapies. Leukemia.

[19] Deeg H.J. (2002). Marrow stroma in MDS: Culprit or bystander?. Leuk. Res..

[20] Fei C., Guo J., Zhao Y., Gu S., Zhao S., Li X., Chang C. (2015). Notch-Hes pathway mediates the impaired osteogenic differentiation of bone marrow mesenchymal stromal cells from myelodysplastic syndromes patients through the down-regulation of Runx2. Am. J. Transl. Res..

[21] Greenberger J.S., FitzGerald T.J., Anklesaria P. (1989). Recent studies of the hematopoietic microenvironment in long-term bone marrow cultures. Immunol. Res..

[22] Jordan C.T., Lemischka I.R. (1990). Clonal and systemic analysis of long-term hematopoiesis in the mouse. Genes Dev..

[23] Kode A., Manavalan J.S., Mosialou I., Bhagat G., Rathinam C.V., Luo N., Khiabanian H., Lee A., Murty V.V., Friedman R. (2014). Leukaemogenesis induced by an activating β-catenin mutation in osteoblasts. Nature.

[24] Lemischka I.R. (1997). Microenvironmental regulation of hematopoietic stem cells. Stem Cells.

[25] Mayani H. (1996). Composition and function of the hemopoietic microenvironment in human myeloid leukemia. Leukemia.

[26] Pavlaki K., Pontikoglou C.G., Demetriadou A., Batsali A.K., Damianaki A., Simantirakis E., Kontakis M., Galanopoulos A., Kotsianidis I., Kastrinaki M.C. (2014). Impaired proliferative potential of bone marrow mesenchymal stromal cells in patients with myelodysplastic syndromes is associated with abnormal WNT signaling pathway. Stem Cells Dev..

[27] Raaijmakers M.H. (2011). Niche contributions to oncogenesis: Emerging concepts and implications for the hematopoietic system. Haematologica.

[28] Ramakrishnan A., Deeg H.J. (2009). A novel role for the marrow microenvironment in initiating and sustaining hematopoietic disease. Expert Opin. Biol. Ther..

[29] Ramakrishnan A., Awaya N., Bryant E., Torok-Storb B. (2006). The stromal component of the marrow microenvironment is not derived from the malignant clone in MDS. Blood.

[30] Varga G., Kiss J., Varkonyi J., Vas V., Farkas P., Paloczi K., Uher F. (2007). Inappropriate Notch activity and limited mesenchymal stem cell plasticity in the bone marrow of patients with myelodysplastic syndromes. Pathol. Oncol. Res..

[31] Calvi L.M., Link D.C. (2015). The hematopoietic stem cell niche in homeostasis and disease. Blood.

[32] Flores-Figueroa E., Gutiérrez-Espindola G., Montesinos J.J., Arana-Trejo R.M., Mayani H. (2002). In vitro characterization of hematopoietic microenvironment cells from patients with myelodysplastic syndrome. Leuk. Res..

[33] Geyh S., Oz S., Cadeddu R.P., Frobel J., Bruckner B., Kundgen A., Fenk R., Bruns I., Zilkens C., Hermsen D. (2013). Insufficient stromal support in MDS results from molecular and functional deficits of mesenchymal stromal cells. Leukemia.

[34] Tauro S., Hepburn M.D., Bowen D.T., Pippard M.J. (2001). Assessment of stromal function, and its potential contribution to deregulation of hematopoiesis in the myelodysplastic syndromes. Haematologica.

[35] Zhang W., Knieling G., Vohwinkel G., Martinez T., Kuse R., Hossfeld D.K., Duhrsen U. (1999). Origin of stroma cells in long-term bone marrow cultures from patients with acute myeloid leukemia. Ann. Hematol..

[36] Walenda T., Bork S., Horn P., Wein F., Saffrich R., Diehlmann A., Eckstein V., Ho A.D., Wagner W. (2010). Co-culture with mesenchymal stromal cells increases proliferation and maintenance of haematopoietic progenitor cells. J. Cell. Mol. Med..

[37] Heo J.S., Choi Y., Kim H.S., Kim H.O. (2016). Comparison of molecular profiles of human mesenchymal stem cells derived from bone marrow, umbilical cord blood, placenta and adipose tissue. Int. J. Mol. Med..

[38] Pittenger M.F., Mackay A.M., Beck S.C., Jaiswal R.K., Douglas R., Mosca J.D., Moorman M.A., Simonetti D.W., Craig S., Marshak D.R. (1999). Multilineage potential of adult human mesenchymal stem cells. Science.

[39] Mendez-Ferrer S., Michurina T.V., Ferraro F., Mazloom A.R., Macarthur B.D., Lira S.A., Scadden D.T., Ma’ayan A., Enikolopov G.N., Frenette P.S. (2010). Mesenchymal and haematopoietic stem cells form a unique bone marrow niche. Nature.

[40] Sacchetti B., Funari A., Michienzi S., di C.S., Piersanti S., Saggio I., Tagliafico E., Ferrari S., Robey P.G., Riminucci M. (2007). Self-renewing osteoprogenitors in bone marrow sinusoids can organize a hematopoietic microenvironment. Cell.

[41] Russell K.C., Phinney D.G., Lacey M.R., Barrilleaux B.L., Meyertholen K.E., O’Connor K.C. (2010). In vitro high-capacity assay to quantify the clonal heterogeneity in trilineage potential of mesenchymal stem cells reveals a complex hierarchy of lineage commitment. Stem Cells.

[42] Wagner W., Feldmann R.E., Seckinger A., Maurer M.H., Wein F., Blake J., Krause U., Kalenka A., Burgers H.F., Saffrich R. (2006). The heterogeneity of human mesenchymal stem cell preparations—Evidence from simultaneous analysis of proteomes and transcriptomes. Exp. Hematol..

[43] Dominici M., Le Blanc K., Mueller I., Slaper-Cortenbach I., Marini F., Krause D., Deans R., Keating A., Prockop D.J., Horwitz E. (2006). Minimal criteria for defining multipotent mesenchymal stromal cells. The international society for cellular therapy position statement. Cytotherapy.

[44] Phinney D.G., Sensebe L. (2013). Mesenchymal stromal cells: Misconceptions and evolving concepts. Cytotherapy.

[45] Horwitz E.M., Le Blanc K., Dominici M., Mueller I., Slaper-Cortenbach I., Marini F.C., Deans R.J., Krause D.S., Keating A. (2005). Clarification of the nomenclature for MSC: The international society for cellular therapy position statement. Cytotherapy.

[46] Bernardo M.E., Fibbe W.E. (2013). Mesenchymal stromal cells: Sensors and switchers of inflammation. Cell Stem Cell.

[47] DeRuiter M.C., Poelmann R.E., VanMunsteren J.C., Mironov V., Markwald R.R., Gittenberger-de Groot A.C. (1997). Embryonic endothelial cells transdifferentiate into mesenchymal cells expressing smooth muscle actins in vivo and in vitro. Circ. Res..

[48] Bennett J.H., Joyner C.J., Triffitt J.T., Owen M.E. (1991). Adipocytic cells cultured from marrow have osteogenic potential. J. Cell Sci..

[49] Dennis J.E., Charbord P. (2002). Origin and differentiation of human and murine stroma. Stem Cells.

[50] Dennis J.E., Carbillet J.P., Caplan A.I., Charbord P. (2002). The STRO-1^+^ marrow cell population is multipotential. Cells Tissues Organs.

[51] De Almeida D.C., Ferreira M.R., Franzen J., Weidner C.I., Frobel J., Zenke M., Costa I.G., Wagner W. (2016). Epigenetic classification of human mesenchymal stromal cells. Stem Cell Rep..

[52] Blasi A., Martino C., Balducci L., Saldarelli M., Soleti A., Navone S.E., Canzi L., Cristini S., Invernici G., Parati E.A. (2011). Dermal fibroblasts display similar phenotypic and differentiation capacity to fat-derived mesenchymal stem cells, but differ in anti-inflammatory and angiogenic potential. Vasc. Cell.

[53] Harichandan A., Buhring H.J. (2011). Prospective isolation of human MSC. Best Pract. Res. Clin. Haematol..

[54] Buhring H.J., Battula V.L., Treml S., Schewe B., Kanz L., Vogel W. (2007). Novel markers for the prospective isolation of human MSC. Ann. N. Y. Acad. Sci..

[55] Buhring H.J., Treml S., Cerabona F., de Z.P., Kanz L., Sobiesiak M. (2009). Phenotypic characterization of distinct human bone marrow-derived MSC subsets. Ann. N. Y. Acad. Sci..

[56] Lv F.J., Tuan R.S., Cheung K.M., Leung V.Y. (2014). Concise review: The surface markers and identity of human mesenchymal stem cells. Stem Cells.

[57] Pinho S., Lacombe J., Hanoun M., Mizoguchi T., Bruns I., Kunisaki Y., Frenette P.S. (2013). PDGFRalpha and CD51 mark human nestin^+^ sphere-forming mesenchymal stem cells capable of hematopoietic progenitor cell expansion. J. Exp. Med..

[58] Quirici N., Soligo D., Bossolasco P., Servida F., Lumini C., Deliliers G.L. (2002). Isolation of bone marrow mesenchymal stem cells by anti-nerve growth factor receptor antibodies. Exp. Hematol..

[59] Samsonraj R.M., Rai B., Sathiyanathan P., Puan K.J., Rotzschke O., Hui J.H., Raghunath M., Stanton L.W., Nurcombe V., Cool S.M. (2015). Establishing criteria for human mesenchymal stem cell potency. Stem Cells.

[60] Frenette P.S., Pinho S., Lucas D., Scheiermann C. (2013). Mesenchymal stem cell: Keystone of the hematopoietic stem cell niche and a stepping-stone for regenerative medicine. Annu. Rev. Immunol..

[61] Sivasubramaniyan K., Lehnen D., Ghazanfari R., Sobiesiak M., Harichandan A., Mortha E., Petkova N., Grimm S., Cerabona F., de Z.P. (2012). Phenotypic and functional heterogeneity of human bone marrow- and amnion-derived MSC subsets. Ann. N. Y. Acad. Sci..

[62] Li H., Ghazanfari R., Zacharaki D., Ditzel N., Isern J., Ekblom M., Mendez-Ferrer S., Kassem M., Scheding S. (2014). Low/negative expression of PDGFR-α identifies the candidate primary mesenchymal stromal cells in adult human bone marrow. Stem Cell Rep..

[63] Krampera M., Galipeau J., Shi Y., Tarte K., Sensebe L. (2013). Immunological characterization of multipotent mesenchymal stromal cells—The international society for cellular therapy (ISCT) working proposal. Cytotherapy.

[64] Keating A. (2012). Mesenchymal stromal cells: New directions. Cell Stem Cell.

[65] Sarugaser R., Hanoun L., Keating A., Stanford W.L., Davies J.E. (2009). Human mesenchymal stem cells self-renew and differentiate according to a deterministic hierarchy. PLoS ONE.

[66] Lopez-Villar O., Garcia J.L., Sanchez-Guijo F.M., Robledo C., Villaron E.M., Hernandez-Campo P., Lopez-Holgado N., Diez-Campelo M., Barbado M.V., Perez-Simon J.A. (2009). Both expanded and uncultured mesenchymal stem cells from MDS patients are genomically abnormal, showing a specific genetic profile for the 5q- syndrome. Leukemia.

[67] Gul-Uludag H., Valencia-Serna J., Kucharski C., Marquez-Curtis L.A., Jiang X., Larratt L., Janowska-Wieczorek A., Uludag H. (2014). Polymeric nanoparticle-mediated silencing of CD44 receptor in CD34 acute myeloid leukemia cells. Leuk. Res..

[68] Aanei C.M., Flandrin P., Eloae F.Z., Carasevici E., Guyotat D., Wattel E., Campos L. (2012). Intrinsic growth deficiencies of mesenchymal stromal cells in myelodysplastic syndromes. Stem Cells Dev..

[69] Jin L., Hope K.J., Zhai Q., Smadja-Joffe F., Dick J.E. (2006). Targeting of CD44 eradicates human acute myeloid leukemic stem cells. Nat. Med..

[70] Wang N.S., Wei M., Ma W.L., Meng W., Zheng W.L. (2014). Knockdown of CD44 enhances chemosensitivity of acute myeloid leukemia cells to ADM and Ara-C. Tumour. Biol..

[71] Quere R., Andradottir S., Brun A.C., Zubarev R.A., Karlsson G., Olsson K., Magnusson M., Cammenga J., Karlsson S. (2011). High levels of the adhesion molecule CD44 on leukemic cells generate acute myeloid leukemia relapse after withdrawal of the initial transforming event. Leukemia.

[72] Krause D.S., Spitzer T.R., Stowell C.P. (2010). The concentration of CD44 is increased in hematopoietic stem cell grafts of patients with acute myeloid leukemia, plasma cell myeloma, and non-Hodgkin lymphoma. Arch. Pathol. Lab. Med..

[73] Huang X., Li D., Li T., Zhao B.O., Chen X. (2015). Prognostic value of the expression of phosphatase and tensin homolog and CD44 in elderly patients with refractory acute myeloid leukemia. Oncol. Lett..

[74] Chen P., Huang H., Wu J., Lu R., Wu Y., Jiang X., Yuan Q., Chen Y. (2015). Bone marrow stromal cells protect acute myeloid leukemia cells from anti-CD44 therapy partly through regulating PI3K/Akt-p27(Kip1) axis. Mol. Carcinog..

[75] Lane S.W., Wang Y.J., Lo C.C., Ragu C., Bullinger L., Sykes S.M., Ferraro F., Shterental S., Lin C.P., Gilliland D.G. (2011). Differential niche and Wnt requirements during acute myeloid leukemia progression. Blood.

[76] ClinicalTrials.gov. https://clinicaltrials.gov/.

[77] Casucci M., Nicolis di R.B., Falcone L., Camisa B., Norelli M., Genovese P., Gentner B., Gullotta F., Ponzoni M., Bernardi M. (2013). CD44v6-targeted T cells mediate potent antitumor effects against acute myeloid leukemia and multiple myeloma. Blood.

[78] Aanei C.M., Eloae F.Z., Flandrin-Gresta P., Tavernier E., Carasevici E., Guyotat D., Campos L. (2011). Focal adhesion protein abnormalities in myelodysplastic mesenchymal stromal cells. Exp. Cell Res..

[79] Zhao Z., Wang Z., Li Q., Li W., You Y., Zou P. (2012). The different immunoregulatory functions of mesenchymal stem cells in patients with low-risk or high-risk myelodysplastic syndromes. PLoS ONE.

[80] Fei C., Zhao Y., Guo J., Gu S., Li X., Chang C. (2014). Senescence of bone marrow mesenchymal stromal cells is accompanied by activation of p53/p21 pathway in myelodysplastic syndromes. Eur. J. Haematol..

[81] Johnson R.C., Kurzer J.H., Greenberg P.L., Gratzinger D. (2014). Mesenchymal stromal cell density is increased in higher grade myelodysplastic syndromes and independently predicts survival. Am. J. Clin. Pathol..

[82] Zheng B., Cao B., Crisan M., Sun B., Li G., Logar A., Yap S., Pollett J.B., Drowley L., Cassino T. (2007). Prospective identification of myogenic endothelial cells in human skeletal muscle. Nat. Biotechnol..

[83] Takashima Y., Era T., Nakao K., Kondo S., Kasuga M., Smith A.G., Nishikawa S. (2007). Neuroepithelial cells supply an initial transient wave of MSC differentiation. Cell.

[84] Corselli M., Chen C.W., Sun B., Yap S., Rubin J.P., Peault B. (2012). The tunica adventitia of human arteries and veins as a source of mesenchymal stem cells. Stem Cells Dev..

[85] Majesky M.W., Dong X.R., Hoglund V., Mahoney W.M., Daum G. (2011). The adventitia: A dynamic interface containing resident progenitor cells. Arterioscler. Thromb. Vasc. Biol..

[86] Chen W.C., Park T.S., Murray I.R., Zimmerlin L., Lazzari L., Huard J., Peault B. (2013). Cellular kinetics of perivascular MSC precursors. Stem Cells Int..

[87] Chen C.W., Corselli M., Peault B., Huard J. (2012). Human blood-vessel-derived stem cells for tissue repair and regeneration. J. Biomed. Biotechnol..

[88] Tavian M., Zheng B., Oberlin E., Crisan M., Sun B., Huard J., Peault B. (2005). The vascular wall as a source of stem cells. Ann. N. Y. Acad. Sci..

[89] Sheng G. (2015). The developmental basis of mesenchymal stem/stromal cells (MSCs). BMC. Dev. Biol..

[90] Vodyanik M.A., Yu J., Zhang X., Tian S., Stewart R., Thomson J.A., Slukvin I.I. (2010). A mesoderm-derived precursor for mesenchymal stem and endothelial cells. Cell Stem Cell.

[91] Slukvin I.I., Vodyanik M. (2011). Endothelial origin of mesenchymal stem cells. Cell Cycle.

[92] Munoz-Chapuli R., Carmona R., Guadix J.A., Macias D., Perez-Pomares J.M. (2005). The origin of the endothelial cells: An evo-devo approach for the invertebrate/vertebrate transition of the circulatory system. Evol. Dev..

[93] Choi K. (2002). The hemangioblast: A common progenitor of hematopoietic and endothelial cells. J. Hematother. Stem Cell Res..

[94] Ciraci E., Della B.S., Salvucci O., Rofani C., Segarra M., Bason C., Molinari A., Maric D., Tosato G., Berardi A.C. (2011). Adult human circulating CD34^−^Lin^−^CD45^−^CD133^−^ cells can differentiate into hematopoietic and endothelial cells. Blood.

[95] Lancrin C., Sroczynska P., Stephenson C., Allen T., Kouskoff V., Lacaud G. (2009). The haemangioblast generates haematopoietic cells through a haemogenic endothelium stage. Nature.

[96] Gunsilius E., Duba H.C., Petzer A.L., Kahler C.M., Grunewald K., Stockhammer G., Gabl C., Dirnhofer S., Clausen J., Gastl G. (2000). Evidence from a leukaemia model for maintenance of vascular endothelium by bone-marrow-derived endothelial cells. Lancet.

[97] Tanaka Y., Sanchez V., Takata N., Yokomizo T., Yamanaka Y., Kataoka H., Hoppe P.S., Schroeder T., Nishikawa S. (2014). Circulation-independent differentiation pathway from extraembryonic mesoderm toward hematopoietic stem cells via hemogenic angioblasts. Cell Rep..

[98] Basak G.W., Yasukawa S., Alfaro A., Halligan S., Srivastava A.S., Min W.P., Minev B., Carrier E. (2009). Human embryonic stem cells hemangioblast express HLA-antigens. J. Transl. Med..

[99] Yamashita J., Itoh H., Hirashima M., Ogawa M., Nishikawa S., Yurugi T., Naito M., Nakao K., Nishikawa S. (2000). Flk1-positive cells derived from embryonic stem cells serve as vascular progenitors. Nature.

[100] Kuprijanov V.V. (1990). Vascular endothelium (review). I. General morphology. 2B: Phylogenesis of the vascular endothelium. Gegenbaurs Morphol. Jahrb..

[101] Green A.R. (2000). Haemangioblast origin of chronic myeloid leukaemia?. Lancet.

[102] Piera-Velazquez S., Li Z., Jimenez S.A. (2011). Role of endothelial-mesenchymal transition (EndoMT) in the pathogenesis of fibrotic disorders. Am. J. Pathol..

[103] Kokudo T., Suzuki Y., Yoshimatsu Y., Yamazaki T., Watabe T., Miyazono K. (2008). Snail is required for TGFbeta-induced endothelial-mesenchymal transition of embryonic stem cell-derived endothelial cells. J. Cell Sci..

[104] Potenta S., Zeisberg E., Kalluri R. (2008). The role of endothelial-to-mesenchymal transition in cancer progression. Br. J. Cancer.

[105] Medici D., Shore E.M., Lounev V.Y., Kaplan F.S., Kalluri R., Olsen B.R. (2010). Conversion of vascular endothelial cells into multipotent stem-like cells. Nat. Med..

[106] Oswald J., Boxberger S., Jorgensen B., Feldmann S., Ehninger G., Bornhauser M., Werner C. (2004). Mesenchymal stem cells can be differentiated into endothelial cells in vitro. Stem Cells.

[107] Crisan M. (2013). Transition of mesenchymal stem/stromal cells to endothelial cells. Stem Cell Res. Ther..

[108] Paggetti J., Haderk F., Seiffert M., Janji B., Distler U., Ammerlaan W., Kim Y.J., Adam J., Lichter P., Solary E. (2015). Exosomes released by chronic lymphocytic leukemia cells induce the transition of stromal cells into cancer-associated fibroblasts. Blood.

[109] Chute J.P., Muramoto G.G., Salter A.B., Meadows S.K., Rickman D.W., Chen B., Himburg H.A., Chao N.J. (2007). Transplantation of vascular endothelial cells mediates the hematopoietic recovery and survival of lethally irradiated mice. Blood.

[110] Salter A.B., Meadows S.K., Muramoto G.G., Himburg H., Doan P., Daher P., Russell L., Chen B., Chao N.J., Chute J.P. (2009). Endothelial progenitor cell infusion induces hematopoietic stem cell reconstitution in vivo. Blood.

[111] Li W., Johnson S.A., Shelley W.C., Yoder M.C. (2004). Hematopoietic stem cell repopulating ability can be maintained in vitro by some primary endothelial cells. Exp. Hematol..

[112] Ding L., Saunders T.L., Enikolopov G., Morrison S.J. (2012). Endothelial and perivascular cells maintain haematopoietic stem cells. Nature.

[113] Winkler I.G., Barbier V., Nowlan B., Jacobsen R.N., Forristal C.E., Patton J.T., Magnani J.L., Levesque J.P. (2012). Vascular niche E-selectin regulates hematopoietic stem cell dormancy, self renewal and chemoresistance. Nat. Med..

[114] Reale A., Melaccio A., Lamanuzzi A., Saltarella I., Dammacco F., Vacca A., Ria R. (2016). Functional and biological role of endothelial precursor cells in tumour progression: A new potential therapeutic target in haematological malignancies. Stem Cells Int..

[115] Asahara T., Masuda H., Takahashi T., Kalka C., Pastore C., Silver M., Kearne M., Magner M., Isner J.M. (1999). Bone marrow origin of endothelial progenitor cells responsible for postnatal vasculogenesis in physiological and pathological neovascularization. Circ. Res..

[116] Buckstein R., Kerbel R., Cheung M., Shaked Y., Chodirker L., Lee C.R., Lenis M., Davidson C., Cussen M.A., Reis M. (2014). Lenalidomide and metronomic melphalan for CMML and higher risk MDS: A phase 2 clinical study with biomarkers of angiogenesis. Leuk. Res..

[117] Cortelezzi A., Fracchiolla N.S., Mazzeo L.M., Silvestris I., Pomati M., Somalvico F., Bertolini F., Mancuso P., Pruneri G.C., Gianelli U. (2005). Endothelial precursors and mature endothelial cells are increased in the peripheral blood of myelodysplastic syndromes. Leuk. Lymphoma.

[118] Della Porta M.G., Malcovati L., Rigolin G.M., Rosti V., Bonetti E., Travaglino E., Boveri E., Galli A., Boggi S., Ciccone M. (2008). Immunophenotypic, cytogenetic and functional characterization of circulating endothelial cells in myelodysplastic syndromes. Leukemia.

[119] Sudhoff T., Germing U., Aul C. (2002). Levels of circulating endothelial adhesion molecules in patients with myelodysplastic syndromes. Int. J. Oncol..

[120] Teofili L., Martini M., Nuzzolo E.R., Capodimonti S., Iachininoto M.G., Cocomazzi A., Fabiani E., Voso M.T., Larocca L.M. (2015). Endothelial progenitor cell dysfunction in myelodysplastic syndromes: Possible contribution of a defective vascular niche to myelodysplasia. Neoplasia.

[121] Agliano A., Martin-Padura I., Mancuso P., Marighetti P., Rabascio C., Pruneri G., Shultz L.D., Bertolini F. (2008). Human acute leukemia cells injected in NOD/LtSz-scid/IL-2Rgamma null mice generate a faster and more efficient disease compared to other NOD/scid-related strains. Int. J. Cancer.

[122] Rigolin G.M., Mauro E., Ciccone M., Fraulini C., Sofritti O., Castoldi G., Cuneo A. (2007). Neoplastic circulating endothelial-like cells in patients with acute myeloid leukaemia. Eur. J. Haematol..

[123] Wierzbowska A., Robak T., Krawczynska A., Wrzesien-Kus A., Pluta A., Cebula B., Smolewski P. (2005). Circulating endothelial cells in patients with acute myeloid leukemia. Eur. J. Haematol..

[124] Wierzbowska A., Robak T., Krawczynska A., Pluta A., Wrzesien-Kus A., Cebula B., Robak E., Smolewski P. (2008). Kinetics and apoptotic profile of circulating endothelial cells as prognostic factors for induction treatment failure in newly diagnosed acute myeloid leukemia patients. Ann. Hematol..

[125] Hanoun M., Zhang D., Mizoguchi T., Pinho S., Pierce H., Kunisaki Y., Lacombe J., Armstrong S.A., Duhrsen U., Frenette P.S. (2014). Acute myelogenous leukemia-induced sympathetic neuropathy promotes malignancy in an altered hematopoietic stem cell niche. Cell Stem Cell.

[126] Hatfield K., Oyan A.M., Ersvaer E., Kalland K.H., Lassalle P., Gjertsen B.T., Bruserud O. (2009). Primary human acute myeloid leukaemia cells increase the proliferation of microvascular endothelial cells through the release of soluble mediators. Br. J. Haematol..

[127] Pezeshkian B., Donnelly C., Tamburo K., Geddes T., Madlambayan G.J. (2013). Leukemia mediated endothelial cell activation modulates leukemia cell susceptibility to chemotherapy through a positive feedback loop mechanism. PLoS ONE.

[128] Sipkins D.A., Wei X., Wu J.W., Runnels J.M., Cote D., Means T.K., Luster A.D., Scadden D.T., Lin C.P. (2005). In vivo imaging of specialized bone marrow endothelial microdomains for tumour engraftment. Nature.

[129] Pizzo R.J., Azadniv M., Guo N., Acklin J., Lacagnina K., Coppage M., Liesveld J.L. (2016). Phenotypic, genotypic, and functional characterization of normal and acute myeloid leukemia-derived marrow endothelial cells. Exp. Hematol..

[130] Drusbosky L., Gars E., Trujillo A., McGee C., Meacham A., Wise E., Scott E.W., Cogle C.R. (2015). Endothelial cell derived angiocrine support of acute myeloid leukemia targeted by receptor tyrosine kinase inhibition. Leuk. Res..

[131] Streubel B., Chott A., Huber D., Exner M., Jager U., Wagner O., Schwarzinger I. (2004). Lymphoma-specific genetic aberrations in microvascular endothelial cells in B-cell lymphomas. N. Engl. J. Med..

[132] Huang X., Saint-Jeannet J.P. (2004). Induction of the neural crest and the opportunities of life on the edge. Dev. Biol..

[133] Korn J., Christ B., Kurz H. (2002). Neuroectodermal origin of brain pericytes and vascular smooth muscle cells. J. Comp. Neurol..

[134] Etchevers H.C., Vincent C., Le Douarin N.M., Couly G.F. (2001). The cephalic neural crest provides pericytes and smooth muscle cells to all blood vessels of the face and forebrain. Development.

[135] Birbrair A., Zhang T., Wang Z.M., Messi M.L., Mintz A., Delbono O. (2015). Pericytes at the intersection between tissue regeneration and pathology. Clin. Sci. Lond..

[136] Hill J., Rom S., Ramirez S.H., Persidsky Y. (2014). Emerging roles of pericytes in the regulation of the neurovascular unit in health and disease. J. Neuroimmune Pharmacol..

[137] Sa-Pereira I., Brites D., Brito M.A. (2012). Neurovascular unit: A focus on pericytes. Mol. Neurobiol..

[138] Winkler E.A., Bell R.D., Zlokovic B.V. (2011). Central nervous system pericytes in health and disease. Nat. Neurosci..

[139] Bergers G., Song S. (2005). The role of pericytes in blood-vessel formation and maintenance. Neuro. Oncol..

[140] Crisan M., Yap S., Casteilla L., Chen C.W., Corselli M., Park T.S., Andriolo G., Sun B., Zheng B., Zhang L. (2008). A perivascular origin for mesenchymal stem cells in multiple human organs. Cell Stem Cell.

[141] Zimmerlin L., Park T.S., Donnenberg V.S., Zambidis E.T., Donnenberg A.D., Shiffman A.M., di Giuseppe A., Bassetto F. (2014). Pericytes: A ubiquitous source of multipotent adult tissue stem cells. Stem Cells in Aesthetic Procedures: Art, Science, and Clinical Techniques.

[142] Armulik A., Genove G., Betsholtz C. (2011). Pericytes: Developmental, physiological, and pathological perspectives, problems, and promises. Dev. Cell.

[143] Brachvogel B., Moch H., Pausch F., Schlotzer-Schrehardt U., Hofmann C., Hallmann R., von der M.K., Winkler T., Poschl E. (2005). Perivascular cells expressing annexin A5 define a novel mesenchymal stem cell-like population with the capacity to differentiate into multiple mesenchymal lineages. Development.

[144] Covas D.T., Panepucci R.A., Fontes A.M., Silva W.A., Orellana M.D., Freitas M.C., Neder L., Santos A.R., Peres L.C., Jamur M.C. (2008). Multipotent mesenchymal stromal cells obtained from diverse human tissues share functional properties and gene-expression profile with CD146^+^ perivascular cells and fibroblasts. Exp. Hematol..

[145] Gokcinar-Yagci B., Uckan-Cetinkaya D., Celebi-Saltik B. (2015). Pericytes: Properties, functions and applications in tissue engineering. Stem Cell Rev..

[146] Tormin A., Li O., Brune J.C., Walsh S., Schutz B., Ehinger M., Ditzel N., Kassem M., Scheding S. (2011). CD146 expression on primary nonhematopoietic bone marrow stem cells is correlated with in situ localization. Blood.

[147] Caplan A.I. (2008). All MSCs are pericytes?. Cell Stem Cell.

[148] Rieske P., Krynska B., Azizi S.A. (2005). Human fibroblast-derived cell lines have characteristics of embryonic stem cells and cells of neuro-ectodermal origin. Differentiation.

[149] Joseph N.M., Mukouyama Y.S., Mosher J.T., Jaegle M., Crone S.A., Dormand E.L., Lee K.F., Meijer D., Anderson D.J., Morrison S.J. (2004). Neural crest stem cells undergo multilineage differentiation in developing peripheral nerves to generate endoneurial fibroblasts in addition to Schwann cells. Development.

[150] Alvarez R., Lee H.L., Hong C., Wang C.Y. (2015). Single CD271 marker isolates mesenchymal stem cells from human dental pulp. Int. J. Oral Sci..

[151] Calabrese G., Giuffrida R., Lo F.D., Parrinello N.L., Forte S., Gulino R., Colarossi C., Schinocca L.R., Giuffrida R., Cardile V. (2015). Potential effect of CD271 on human mesenchymal stromal cell proliferation and differentiation. Int. J. Mol. Sci..

[152] Morrison S.J., White P.M., Zock C., Anderson D.J. (1999). Prospective identification, isolation by flow cytometry, and in vivo self-renewal of multipotent mammalian neural crest stem cells. Cell.

[153] Poloni A., Maurizi G., Rosini V., Mondini E., Mancini S., Discepoli G., Biasio S., Battaglini G., Felicetti S., Berardinelli E. (2009). Selection of CD271^+^ cells and human AB serum allows a large expansion of mesenchymal stromal cells from human bone marrow. Cytotherapy.

[154] Churchman S.M., Ponchel F., Boxall S.A., Cuthbert R., Kouroupis D., Roshdy T., Giannoudis P.V., Emery P., McGonagle D., Jones E.A. (2012). Transcriptional profile of native CD271^+^ multipotential stromal cells: Evidence for multiple fates, with prominent osteogenic and Wnt pathway signaling activity. Arthritis Rheum..

[155] Flores-Figueroa E., Varma S., Montgomery K., Greenberg P.L., Gratzinger D. (2012). Distinctive contact between CD34^+^ hematopoietic progenitors and CXCL12^+^ CD271^+^ mesenchymal stromal cells in benign and myelodysplastic bone marrow. Lab. Investig..

[156] Lendahl U., Zimmerman L.B., McKay R.D. (1990). CNS stem cells express a new class of intermediate filament protein. Cell.

[157] Isern J., Garcia-Garcia A., Martin A.M., Arranz L., Martin-Perez D., Torroja C., Sanchez-Cabo F., Mendez-Ferrer S. (2014). The neural crest is a source of mesenchymal stem cells with specialized hematopoietic stem cell niche function. Elife.

[158] Wislet-Gendebien S., Laudet E., Neirinckx V., Alix P., Leprince P., Glejzer A., Poulet C., Hennuy B., Sommer L., Shakhova O. (2012). Mesenchymal stem cells and neural crest stem cells from adult bone marrow: Characterization of their surprising similarities and differences. Cell. Mol. Life Sci..

[159] Suzuki S., Namiki J., Shibata S., Mastuzaki Y., Okano H. (2010). The neural stem/progenitor cell marker nestin is expressed in proliferative endothelial cells, but not in mature vasculature. J. Histochem. Cytochem..

[160] Matsuda Y., Hagio M., Ishiwata T. (2013). Nestin: A novel angiogenesis marker and possible target for tumor angiogenesis. World J. Gastroenterol..

[161] Kishaba Y., Matsubara D., Niki T. (2010). Heterogeneous expression of nestin in myofibroblasts of various human tissues. Pathol. Int..

[162] Krupkova O., Loja T., Zambo I., Veselska R. (2010). Nestin expression in human tumors and tumor cell lines. Neoplasma.

[163] Wright D.E., Bowman E.P., Wagers A.J., Butcher E.C., Weissman I.L. (2002). Hematopoietic stem cells are uniquely selective in their migratory response to chemokines. J. Exp. Med..

[164] Colmone A., Amorim M., Pontier A.L., Wang S., Jablonski E., Sipkins D.A. (2008). Leukemic cells create bone marrow niches that disrupt the behavior of normal hematopoietic progenitor cells. Science.

[165] Tavor S., Petit I., Porozov S., Avigdor A., Dar A., Leider-Trejo L., Shemtov N., Deutsch V., Naparstek E., Nagler A. (2004). CXCR4 regulates migration and development of human acute myelogenous leukemia stem cells in transplanted NOD/SCID mice. Cancer Res..

[166] Yamazaki S., Ema H., Karlsson G., Yamaguchi T., Miyoshi H., Shioda S., Taketo M.M., Karlsson S., Iwama A., Nakauchi H. (2011). Nonmyelinating Schwann cells maintain hematopoietic stem cell hibernation in the bone marrow niche. Cell.

[167] Sanchez-Ramos J., Song S., Cardozo-Pelaez F., Hazzi C., Stedeford T., Willing A., Freeman T.B., Saporta S., Janssen W., Patel N. (2000). Adult bone marrow stromal cells differentiate into neural cells in vitro. Exp. Neurol..

[168] Woodbury D., Schwarz E.J., Prockop D.J., Black I.B. (2000). Adult rat and human bone marrow stromal cells differentiate into neurons. J. Neurosci. Res..

[169] Tzeng H.H., Hsu C.H., Chung T.H., Lee W.C., Lin C.H., Wang W.C., Hsiao C.Y., Leu Y.W., Wang T.H. (2015). Cell signaling and differential protein expression in neuronal differentiation of bone marrow mesenchymal stem cells with hypermethylated Salvador/Warts/Hippo (SWH) pathway genes. PLoS ONE.

[170] Bossolasco P., Cova L., Calzarossa C., Rimoldi S.G., Borsotti C., Deliliers G.L., Silani V., Soligo D., Polli E. (2005). Neuro-glial differentiation of human bone marrow stem cells in vitro. Exp. Neurol..

[171] Bae K.S., Park J.B., Kim H.S., Kim D.S., Park D.J., Kang S.J. (2011). Neuron-like differentiation of bone marrow-derived mesenchymal stem cells. Yonsei Med. J..

[172] Zhang H.T., Liu Z.L., Yao X.Q., Yang Z.J., Xu R.X. (2012). Neural differentiation ability of mesenchymal stromal cells from bone marrow and adipose tissue: A comparative study. Cytotherapy.

[173] Widera D., Heimann P., Zander C., Imielski Y., Heidbreder M., Heilemann M., Kaltschmidt C., Kaltschmidt B. (2011). Schwann cells can be reprogrammed to multipotency by culture. Stem Cells Dev..

[174] Weber M., Apostolova G., Widera D., Mittelbronn M., Dechant G., Kaltschmidt B., Rohrer H. (2015). Alternative generation of CNS neural stem cells and PNS derivatives from neural crest-derived peripheral stem cells. Stem Cells.

[175] Hermann A., Maisel M., Storch A. (2006). Epigenetic conversion of human adult bone mesodermal stromal cells into neuroectodermal cell types for replacement therapy of neurodegenerative disorders. Expert Opin. Biol. Ther..

[176] Coste C., Neirinckx V., Gothot A., Wislet S., Rogister B. (2015). Are neural crest stem cells the missing link between hematopoietic and neurogenic niches?. Front. Cell Neurosci..

[177] Abe-Suzuki S., Kurata M., Abe S., Onishi I., Kirimura S., Nashimoto M., Murayama T., Hidaka M., Kitagawa M. (2014). CXCL12^+^ stromal cells as bone marrow niche for CD34^+^ hematopoietic cells and their association with disease progression in myelodysplastic syndromes. Lab. Investig..

[178] Sharma M., Afrin F., Satija N., Tripathi R.P., Gangenahalli G.U. (2011). Stromal-derived factor-1/CXCR4 signaling: Indispensable role in homing and engraftment of hematopoietic stem cells in bone marrow. Stem Cells Dev..

[179] Fuhler G.M., Drayer A.L., Olthof S.G., Schuringa J.J., Coffer P.J., Vellenga E. (2008). Reduced activation of protein kinase B, Rac, and F-actin polymerization contributes to an impairment of stromal cell derived factor-1 induced migration of CD34^+^ cells from patients with myelodysplasia. Blood.

[180] Matsuda M., Morita Y., Hanamoto H., Tatsumi Y., Maeda Y., Kanamaru A. (2004). CD34^+^ progenitors from MDS patients are unresponsive to SDF-1, despite high levels of SDF-1 in bone marrow plasma. Leukemia.

[181] Kim H.Y., Oh Y.S., Song I.C., Kim S.W., Lee H.J., Yun H.J., Kim S., Jo D.Y. (2013). Endogenous stromal cell-derived factor-1 (CXCL12) supports autonomous growth of acute myeloid leukemia cells. Leuk. Res..

[182] Kim J.A., Shim J.S., Lee G.Y., Yim H.W., Kim T.M., Kim M., Leem S.H., Lee J.W., Min C.K., Oh I.H. (2015). Microenvironmental remodeling as a parameter and prognostic factor of heterogeneous leukemogenesis in acute myelogenous leukemia. Cancer Res..

[183] Conneally E., Cashman J., Petzer A., Eaves C. (1997). Expansion in vitro of transplantable human cord blood stem cells demonstrated using a quantitative assay of their lympho-myeloid repopulating activity in nonobese diabetic-scid/scid mice. Proc. Natl. Acad. Sci. USA.

[184] Zhang Y., Patel S., Abdelouahab H., Wittner M., Willekens C., Shen S., Betems A., Joulin V., Opolon P., Bawa O. (2012). CXCR4 inhibitors selectively eliminate CXCR4-expressing human acute myeloid leukemia cells in NOG mouse model. Cell Death. Dis..

[185] Cho B.S., Zeng Z., Mu H., Wang Z., Konoplev S., McQueen T., Protopopova M., Cortes J., Marszalek J.R., Peng S.B. (2015). Antileukemia activity of the novel peptidic CXCR4 antagonist LY2510924 as monotherapy and in combination with chemotherapy. Blood.

[186] Uy G.L., Rettig M.P., Motabi I.H., McFarland K., Trinkaus K.M., Hladnik L.M., Kulkarni S., Abboud C.N., Cashen A.F., Stockerl-Goldstein K.E. (2012). A phase 1/2 study of chemosensitization with the CXCR4 antagonist plerixafor in relapsed or refractory acute myeloid leukemia. Blood.

[187] Mendez-Ferrer S., Lucas D., Battista M., Frenette P.S. (2008). Haematopoietic stem cell release is regulated by circadian oscillations. Nature.

[188] Katayama Y., Battista M., Kao W.M., Hidalgo A., Peired A.J., Thomas S.A., Frenette P.S. (2006). Signals from the sympathetic nervous system regulate hematopoietic stem cell egress from bone marrow. Cell.

[189] Lucas D., Battista M., Shi P.A., Isola L., Frenette P.S. (2008). Mobilized hematopoietic stem cell yield depends on species-specific circadian timing. Cell Stem Cell.

[190] Mendez-Ferrer S., Chow A., Merad M., Frenette P.S. (2009). Circadian rhythms influence hematopoietic stem cells. Curr. Opin. Hematol..

[191] Li H., Fong C., Chen Y., Cai G., Yang M. (2010). β_2_- and β_3_-, but not beta1-adrenergic receptors are involved in osteogenesis of mouse mesenchymal stem cells via cAMP/PKA signaling. Arch. Biochem. Biophys..

[192] Mendez-Ferrer S., Battista M., Frenette P.S. (2010). Cooperation of β_2_- and β_3_-adrenergic receptors in hematopoietic progenitor cell mobilization. Ann. N. Y. Acad. Sci..

[193] Du Z., Wang L., Zhao Y., Cao J., Wang T., Liu P., Zhang Y., Yang X., Cheng X., Liu B. (2014). Sympathetic denervation-induced MSC mobilization in distraction osteogenesis associates with inhibition of MSC migration and osteogenesis by norepinephrine/adrb3. PLoS ONE.

[194] Sugiyama T., Kohara H., Noda M., Nagasawa T. (2006). Maintenance of the hematopoietic stem cell pool by CXCL12-CXCR4 chemokine signaling in bone marrow stromal cell niches. Immunity.

[195] Del Toro R., Mendez-Ferrer S. (2013). Autonomic regulation of hematopoiesis and cancer. Haematologica.

[196] Arranz L., Sanchez-Aguilera A., Martin-Perez D., Isern J., Langa X., Tzankov A., Lundberg P., Muntion S., Tzeng Y.S., Lai D.M. (2014). Neuropathy of haematopoietic stem cell niche is essential for myeloproliferative neoplasms. Nature.

[197] Battula V.L., Evans K.W., Hollier B.G., Shi Y., Marini F.C., Ayyanan A., Wang R.Y., Brisken C., Guerra R., Andreeff M. (2010). Epithelial-mesenchymal transition-derived cells exhibit multilineage differentiation potential similar to mesenchymal stem cells. Stem Cells.

[198] Dong C.Y., Liu X.Y., Wang N., Wang L.N., Yang B.X., Ren Q., Liang H.Y., Ma X.T. (2014). Twist-1, a novel regulator of hematopoietic stem cell self-renewal and myeloid lineage development. Stem Cells.

[199] Merindol N., Riquet A., Szablewski V., Eliaou J.F., Puisieux A., Bonnefoy N. (2014). The emerging role of Twist proteins in hematopoietic cells and hematological malignancies. Blood Cancer J..

[200] Isenmann S., Arthur A., Zannettino A.C., Turner J.L., Shi S., Glackin C.A., Gronthos S. (2009). TWIST family of basic helix-loop-helix transcription factors mediate human mesenchymal stem cell growth and commitment. Stem Cells.

[201] Cakouros D., Raices R.M., Gronthos S., Glackin C.A. (2010). Twist-ing cell fate: Mechanistic insights into the role of twist in lineage specification/differentiation and tumorigenesis. J. Cell. Biochem..

[202] Norozi F., Ahmadzadeh A., Shahjahani M., Shahrabi S., Saki N. (2016). Twist as a new prognostic marker in hematological malignancies. Clin. Transl. Oncol..

[203] Zhang P., Hu P., Shen H., Yu J., Liu Q., Du J. (2014). Prognostic role of Twist or Snail in various carcinomas: A systematic review and meta-analysis. Eur. J. Clin. Investig..

[204] Cosset E., Hamdan G., Jeanpierre S., Voeltzel T., Sagorny K., Hayette S., Mahon F.X., Dumontet C., Puisieux A., Nicolini F.E. (2011). Deregulation of TWIST-1 in the CD34^+^ compartment represents a novel prognostic factor in chronic myeloid leukemia. Blood.

[205] Li X., Marcondes A.M., Gooley T.A., Deeg H.J. (2010). The helix-loop-helix transcription factor TWIST is dysregulated in myelodysplastic syndromes. Blood.

[206] Mhyre A.J., Marcondes A.M., Spaulding E.Y., Deeg H.J. (2009). Stroma-dependent apoptosis in clonal hematopoietic precursors correlates with expression of PYCARD. Blood.

[207] Raval A., Lucas D.M., Matkovic J.J., Bennett K.L., Liyanarachchi S., Young D.C., Rassenti L., Kipps T.J., Grever M.R., Byrd J.C. (2005). TWIST2 demonstrates differential methylation in immunoglobulin variable heavy chain mutated and unmutated chronic lymphocytic leukemia. J. Clin. Oncol..

[208] Thathia S.H., Ferguson S., Gautrey H.E., van Otterdijk S.D., Hili M., Rand V., Moorman A.V., Meyer S., Brown R., Strathdee G. (2012). Epigenetic inactivation of TWIST2 in acute lymphoblastic leukemia modulates proliferation, cell survival and chemosensitivity. Haematologica.

[209] Zhang X., Ma W., Cui J., Yao H., Zhou H., Ge Y., Xiao L., Hu X., Liu B.H., Yang J. (2015). Regulation of p21 by TWIST2 contributes to its tumor-suppressor function in human acute myeloid leukemia. Oncogene.

[210] Chen C.C., You J.Y., Gau J.P., Huang C.E., Chen Y.Y., Tsai Y.H., Chou H.J., Lung J., Yang M.H. (2015). Favorable clinical outcome and unique characteristics in association with Twist1 overexpression in de novo acute myeloid leukemia. Blood Cancer J..

[211] Wang N., Guo D., Zhao Y.Y., Dong C.Y., Liu X.Y., Yang B.X., Wang S.W., Wang L., Liu Q.G., Ren Q. (2015). TWIST-1 promotes cell growth, drug resistance and progenitor clonogenic capacities in myeloid leukemia and is a novel poor prognostic factor in acute myeloid leukemia. Oncotarget.

[212] Khan M.A., Chen H.C., Zhang D., Fu J. (2013). Twist: A molecular target in cancer therapeutics. Tumour. Biol..

[213] Percio S., Coltella N., Grisanti S., Bernardi R., Pattini L. (2014). A HIF-1 network reveals characteristics of epithelial-mesenchymal transition in acute promyelocytic leukemia. Genome Med..

[214] Piquer-Gil M., Garcia-Verdugo J.M., Zipancic I., Sanchez M.J., Alvarez-Dolado M. (2009). Cell fusion contributes to pericyte formation after stroke. J. Cereb. Blood Flow Metab..

[215] Cantoni S., Bianchi F., Galletti M., Olivi E., Alviano F., Galie N., Ventura C. (2015). Occurring of in vitro functional vasculogenic pericytes from human circulating early endothelial precursor cell culture. Stem Cells Int..

[216] Kramann R., DiRocco D.P., Humphreys B.D. (2013). Understanding the origin, activation and regulation of matrix-producing myofibroblasts for treatment of fibrotic disease. J. Pathol..

[217] Díaz-Flores L., Gutierrez R., Garcia M.P., Saez F.J., Díaz-Flores L., Valladares F., Madrid J.F. (2014). CD34^+^ stromal cells/fibroblasts/fibrocytes/telocytes as a tissue reserve and a principal source of mesenchymal cells. Location, morphology, function and role in pathology. Histol. Histopathol..

[218] Butler J.M., Nolan D.J., Vertes E.L., Varnum-Finney B., Kobayashi H., Hooper A.T., Seandel M., Shido K., White I.A., Kobayashi M. (2010). Endothelial cells are essential for the self-renewal and repopulation of Notch-dependent hematopoietic stem cells. Cell Stem Cell.

[219] Calvi L.M., Adams G.B., Weibrecht K.W., Weber J.M., Olson D.P., Knight M.C., Martin R.P., Schipani E., Divieti P., Bringhurst F.R. (2003). Osteoblastic cells regulate the haematopoietic stem cell niche. Nature.

[220] Calvi L.M. (2013). Osteolineage cells and regulation of the hematopoietic stem cell. Best Pract. Res. Clin. Haematol..

[221] Chow A., Lucas D., Hidalgo A., Mendez-Ferrer S., Hashimoto D., Scheiermann C., Battista M., Leboeuf M., Prophete C., van Rooijen N. (2011). Bone marrow CD169^+^ macrophages promote the retention of hematopoietic stem and progenitor cells in the mesenchymal stem cell niche. J. Exp. Med..

[222] Chow A., Huggins M., Ahmed J., Hashimoto D., Lucas D., Kunisaki Y., Pinho S., Leboeuf M., Noizat C., van Rooijen N. (2013). CD169^+^ macrophages provide a niche promoting erythropoiesis under homeostasis and stress. Nat. Med..

[223] Davies L.C., Jenkins S.J., Allen J.E., Taylor P.R. (2013). Tissue-resident macrophages. Nat. Immunol..

[224] Ehninger A., Trumpp A. (2011). The bone marrow stem cell niche grows up: Mesenchymal stem cells and macrophages move in. J. Exp. Med..

[225] Fujisaki J., Wu J., Carlson A.L., Silberstein L., Putheti P., Larocca R., Gao W., Saito T.I., Lo C.C., Tsuyuzaki H. (2011). In vivo imaging of Treg cells providing immune privilege to the haematopoietic stem-cell niche. Nature.

[226] Hoffman C.M., Calvi L.M. (2014). Minireview: Complexity of hematopoietic stem cell regulation in the bone marrow microenvironment. Mol. Endocrinol..

[227] Kiel M.J., Yilmaz O.H., Iwashita T., Yilmaz O.H., Terhorst C., Morrison S.J. (2005). SLAM family receptors distinguish hematopoietic stem and progenitor cells and reveal endothelial niches for stem cells. Cell.

[228] Kobayashi H., Butler J.M., O’Donnell R., Kobayashi M., Ding B.S., Bonner B., Chiu V.K., Nolan D.J., Shido K., Benjamin L. (2010). Angiocrine factors from Akt-activated endothelial cells balance self-renewal and differentiation of haematopoietic stem cells. Nat. Cell Biol..

[229] Kollet O., Dar A., Shivtiel S., Kalinkovich A., Lapid K., Sztainberg Y., Tesio M., Samstein R.M., Goichberg P., Spiegel A. (2006). Osteoclasts degrade endosteal components and promote mobilization of hematopoietic progenitor cells. Nat. Med..

[230] Lawal R.A., Calvi L.M. (2011). The niche as a target for hematopoietic manipulation and regeneration. Tissue Eng. Part B Rev..

[231] Lymperi S., Ersek A., Ferraro F., Dazzi F., Horwood N.J. (2011). Inhibition of osteoclast function reduces hematopoietic stem cell numbers in vivo. Blood.

[232] Mansour A., Wakkach A., Blin-Wakkach C. (2012). Role of osteoclasts in the hematopoietic stem cell niche formation. Cell Cycle.

[233] Naveiras O., Nardi V., Wenzel P.L., Hauschka P.V., Fahey F., Daley G.Q. (2009). Bone-marrow adipocytes as negative regulators of the haematopoietic microenvironment. Nature.

[234] Sadahira Y., Mori M. (1999). Role of the macrophage in erythropoiesis. Pathol. Int..

[235] Smith J.N., Calvi L.M. (2013). Concise review: Current concepts in bone marrow microenvironmental regulation of hematopoietic stem and progenitor cells. Stem Cells.

[236] Taichman R.S., Reilly M.J., Emerson S.G. (1996). Human osteoblasts support human hematopoietic progenitor cells in vitro bone marrow cultures. Blood.

[237] Winkler I.G., Sims N.A., Pettit A.R., Barbier V., Nowlan B., Helwani F., Poulton I.J., van Rooijen N., Alexander K.A., Raggatt L.J. (2010). Bone marrow macrophages maintain hematopoietic stem cell (HSC) niches and their depletion mobilizes HSCs. Blood.

[238] Schofield R. (1978). The relationship between the spleen colony-forming cell and the haemopoietic stem cell. Blood Cells.

[239] Turksen K. (2015). Stem Cell Biology and Regenerative Medicine: Tissue-Specific Stem Cell Niche.

[240] Ding L., Morrison S.J. (2013). Haematopoietic stem cells and early lymphoid progenitors occupy distinct bone marrow niches. Nature.

[241] Doan P.L., Chute J.P. (2012). The vascular niche: Home for normal and malignant hematopoietic stem cells. Leukemia.

[242] Zhang J., Niu C., Ye L., Huang H., He X., Tong W.G., Ross J., Haug J., Johnson T., Feng J.Q. (2003). Identification of the haematopoietic stem cell niche and control of the niche size. Nature.

[243] Krause D.S., Scadden D.T. (2015). A hostel for the hostile: The bone marrow niche in hematologic neoplasms. Haematologica.

[244] Ma S., Xie N., Li W., Yuan B., Shi Y., Wang Y. (2014). Immunobiology of mesenchymal stem cells. Cell Death. Differ..

[245] Oh M., Nor J.E. (2015). The perivascular niche and self-renewal of stem cells. Front. Physiol..

[246] Guerrouahen B.S., Al-Hijji I., Tabrizi A.R. (2011). Osteoblastic and vascular endothelial niches, their control on normal hematopoietic stem cells, and their consequences on the development of leukemia. Stem Cells Int..

[247] Yin T., Li L. (2006). The stem cell niches in bone. J. Clin. Investig..

[248] Sugiyama T., Nagasawa T. (2012). Bone marrow niches for hematopoietic stem cells and immune cells. Inflamm. Allergy Drug Targets.

[249] Kunisaki Y., Bruns I., Scheiermann C., Ahmed J., Pinho S., Zhang D., Mizoguchi T., Wei Q., Lucas D., Ito K. (2013). Arteriolar niches maintain haematopoietic stem cell quiescence. Nature.

[250] Omatsu Y., Sugiyama T., Kohara H., Kondoh G., Fujii N., Kohno K., Nagasawa T. (2010). The essential functions of adipo-osteogenic progenitors as the hematopoietic stem and progenitor cell niche. Immunity.

[251] Bruns I., Lucas D., Pinho S., Ahmed J., Lambert M.P., Kunisaki Y., Scheiermann C., Schiff L., Poncz M., Bergman A. (2014). Megakaryocytes regulate hematopoietic stem cell quiescence through CXCL4 secretion. Nat. Med..

[252] Zhao M., Perry J.M., Marshall H., Venkatraman A., Qian P., He X.C., Ahamed J., Li L. (2014). Megakaryocytes maintain homeostatic quiescence and promote post-injury regeneration of hematopoietic stem cells. Nat. Med..

[253] Nakamura-Ishizu A., Takubo K., Fujioka M., Suda T. (2014). Megakaryocytes are essential for HSC quiescence through the production of thrombopoietin. Biochem. Biophys. Res. Commun..

[254] Nakamura-Ishizu A., Takubo K., Kobayashi H., Suzuki-Inoue K., Suda T. (2015). CLEC-2 in megakaryocytes is critical for maintenance of hematopoietic stem cells in the bone marrow. J. Exp. Med..

[255] Chen W.M., Chen Z.X., Cen J.N., He J., Jiao X.L., Pan J.L., Qiu Q.C., Dai L., Liu D.D. (2008). Osteoblasts from patients with myelodysplastic syndrome express multiple cytokines and support hematopoietic progenitor cell survival in vitro. Zhongguo Shi Yan Xue Ye Xue Za Zhi.

[256] Fei C., Zhao Y., Gu S., Guo J., Zhang X., Li X., Chang C. (2014). Impaired osteogenic differentiation of mesenchymal stem cells derived from bone marrow of patients with lower-risk myelodysplastic syndromes. Tumour. Biol..

[257] Schajnovitz A., Scadden D.T. (2014). Bone’s dark side: Mutated osteoblasts implicated in leukemia. Cell Res..

[258] Nybakken G., Gratzinger D. (2016). Myelodysplastic syndrome macrophages have aberrant iron storage and heme oxygenase-1 expression. Leuk. Lymphoma.

[259] Aggarwal N., Swerdlow S.H., TenEyck S.P., Boyiadzis M., Felgar R.E. (2015). Natural killer cell (NK) subsets and NK-like T-cell populations in acute myeloid leukemias and myelodysplastic syndromes. Cytom. B Clin. Cytom..

[260] Epling-Burnette P.K., Bai F., Painter J.S., Rollison D.E., Salih H.R., Krusch M., Zou J., Ku E., Zhong B., Boulware D. (2007). Reduced natural killer (NK) function associated with high-risk myelodysplastic syndrome (MDS) and reduced expression of activating NK receptors. Blood.

[261] Marcondes A.M., Mhyre A.J., Stirewalt D.L., Kim S.H., Dinarello C.A., Deeg H.J. (2008). Dysregulation of IL-32 in myelodysplastic syndrome and chronic myelomonocytic leukemia modulates apoptosis and impairs NK function. Proc. Natl. Acad. Sci. USA.

[262] Fujii S., Shimizu K., Klimek V., Geller M.D., Nimer S.D., Dhodapkar M.V. (2003). Severe and selective deficiency of interferon-γ-producing invariant natural killer T cells in patients with myelodysplastic syndromes. Br. J. Haematol..

[263] Yoneda K., Morii T., Nieda M., Tsukaguchi N., Amano I., Tanaka H., Yagi H., Narita N., Kimura H. (2005). The peripheral blood Valpha24^+^ NKT cell numbers decrease in patients with haematopoietic malignancy. Leuk. Res..

[264] Bouchliou I., Miltiades P., Nakou E., Spanoudakis E., Goutzouvelidis A., Vakalopoulou S., Garypidou V., Kotoula V., Bourikas G., Tsatalas C. (2011). Th17 and Foxp3^+^ T regulatory cell dynamics and distribution in myelodysplastic syndromes. Clin. Immunol..

[265] Fozza C., Longinotti M. (2013). The role of T-cells in the pathogenesis of myelodysplastic syndromes: Passengers and drivers. Leuk. Res..

[266] Mailloux A.W., Epling-Burnette P.K. (2013). Effector memory regulatory T-cell expansion marks a pivotal point of immune escape in myelodysplastic syndromes. Oncoimmunology.

[267] Ge M., Zheng Y., Li X., Lu S., Li H., Chen F., Chen D., Shao Y., Shi J., Feng S. (2013). Differential expression profile of Th1/Th17/Th2-related chemokines and their receptors in patients with acquired bone marrow failure syndromes. Hum. Immunol..

[268] Shao L.L., Zhang L., Hou Y., Yu S., Liu X.G., Huang X.Y., Sun Y.X., Tian T., He N., Ma D.X. (2012). Th22 cells as well as Th17 cells expand differentially in patients with early-stage and late-stage myelodysplastic syndrome. PLoS ONE.

[269] Sand K.E., Rye K.P., Mannsaker B., Bruserud O., Kittang A.O. (2013). Expression patterns of chemokine receptors on circulating T cells from myelodysplastic syndrome patients. Oncoimmunology.

[270] Davison G.M., Novitzky N., Abdulla R. (2013). Monocyte derived dendritic cells have reduced expression of co-stimulatory molecules but are able to stimulate autologous T-cells in patients with MDS. Hematol. Oncol. Stem Cell Ther..

[271] Kerkhoff N., Bontkes H.J., Westers T.M., de Gruijl T.D., Kordasti S., van de Loosdrecht A.A. (2013). Dendritic cells in myelodysplastic syndromes: From pathogenesis to immunotherapy. Immunotherapy.

[272] Meyerson H.J., Osei E., Schweitzer K., Blidaru G., Edinger A., Schlegelmilch J., Awadallah A., Goyal T. (2015). CD1c+ myeloid dendritic cells in myeloid neoplasia. Cytom. B Clin. Cytom..

[273] Chen X., Eksioglu E.A., Zhou J., Zhang L., Djeu J., Fortenbery N., Epling-Burnette P., van Bijnen S., Dolstra H., Cannon J. (2013). Induction of myelodysplasia by myeloid-derived suppressor cells. J. Clin. Investig..

[274] Ferrer R.A., Wobus M., List C., Wehner R., Schönefeldt C., Brocard B., Mohr B., Rauner M., Schmitz M., Stiehler M. (2013). Mesenchymal stromal cells from patients with myelodyplastic syndrome display distinct functional alterations that are modulated by lenalidomide. Haematologica.

[275] Tennant G.B., Walsh V., Truran L.N., Edwards P., Mills K.I., Burnett A.K. (2000). Abnormalities of adherent layers grown from bone marrow of patients with myelodysplasia. Br. J. Haematol..

[276] Li X., Marcondes A.M., Ragoczy T., Telling A., Deeg H.J. (2013). Effect of intravenous coadministration of human stroma cell lines on engraftment of long-term repopulating clonal myelodysplastic syndrome cells in immunodeficient mice. Blood Cancer J..

[277] Li X., Deeg H.J. (2014). Murine xenogeneic models of myelodysplastic syndrome: An essential role for stroma cells. Exp. Hematol..

[278] Li Y., Chen S., Yuan J., Yang Y., Li J., Wu X., Freund M., Pollok K., Hanenberg H., Goebel W.S. (2009). Mesenchymal stem/progenitor cells promote the reconstitution of exogenous hematopoietic stem cells in Fancg-/- mice in vivo. Blood.

[279] Medyouf H., Mossner M., Jann J.C., Nolte F., Raffel S., Herrmann C., Lier A., Eisen C., Nowak V., Zens B. (2014). Myelodysplastic cells in patients reprogram mesenchymal stromal cells to establish a transplantable stem cell niche disease unit. Cell Stem Cell.

[280] Muguruma Y., Matsushita H., Yahata T., Yumino S., Tanaka Y., Miyachi H., Ogawa Y., Kawada H., Ito M., Ando K. (2011). Establishment of a xenograft model of human myelodysplastic syndromes. Haematologica.

[281] Raaijmakers M.H., Mukherjee S., Guo S., Zhang S., Kobayashi T., Schoonmaker J.A., Ebert B.L., Al-Shahrour F., Hasserjian R.P., Scadden E.O. (2010). Bone progenitor dysfunction induces myelodysplasia and secondary leukaemia. Nature.

[282] Kerbauy D.M., Lesnikov V., Torok-Storb B., Bryant E., Deeg H.J. (2004). Engraftment of distinct clonal MDS-derived hematopoietic precursors in NOD/SCID-β_2_-microglobulin-deficient mice after intramedullary transplantation of hematopoietic and stromal cells. Blood.

[283] Li Y., Durig J., Gobel M., Hanoun M., Klein-Hitpass L., Duhrsen U. (2015). Functional abnormalities and changes in gene expression in fibroblasts and macrophages from the bone marrow of patients with acute myeloid leukemia. Int. J. Hematol..

[284] Tang M., Acheampong D.O., Wang Y., Xie W., Wang M., Zhang J. (2016). Tumoral NKG2D alters cell cycle of acute myeloid leukemic cells and reduces NK cell-mediated immune surveillance. Immunol Res.

[285] Sandoval-Borrego D., Moreno-Lafont M.C., Vazquez-Sanchez E.A., Gutierrez-Hoya A., Lopez-Santiago R., Montiel-Cervantes L.A., Ramirez-Saldana M., Vela-Ojeda J. (2016). Overexpression of CD158 and NKG2A Inhibitory Receptors and Underexpression of NKG2D and NKp46 Activating Receptors on NK cells in Acute Myeloid Leukemia. Arch. Med. Res..

[286] Chretien A.S., Granjeaud S., Gondois-Rey F., Harbi S., Orlanducci F., Blaise D., Vey N., Arnoulet C., Fauriat C., Olive D. (2015). Increased NK Cell Maturation in Patients with Acute Myeloid Leukemia. Front. Immunol..

[287] Najera Chuc A.E., Cervantes L.A., Retiguin F.P., Ojeda J.V., Maldonado E.R. (2012). Low number of invariant NKT cells is associated with poor survival in acute myeloid leukemia. J. Cancer Res. Clin. Oncol..

[288] Yang W., Xu Y. (2013). Clinical significance of Treg cell frequency in acute myeloid leukemia. Int. J. Hematol..

[289] Memarian A., Nourizadeh M., Masoumi F., Tabrizi M., Emami A.H., Alimoghaddam K., Hadjati J., Mirahmadian M., Jeddi-Tehrani M. (2013). Upregulation of CD200 is associated with Foxp3^+^ regulatory T cell expansion and disease progression in acute myeloid leukemia. Tumour Biol..

[290] Tian T., Yu S., Liu L., Xue F., Yuan C., Wang M., Ji C., Ma D. (2015). The Profile of T Helper Subsets in Bone Marrow Microenvironment Is Distinct for Different Stages of Acute Myeloid Leukemia Patients and Chemotherapy Partly Ameliorates These Variations. PLoS ONE.

[291] Musuraca G., De M.S., Napolitano R., Papayannidis C., Guadagnuolo V., Fabbri F., Cangini D., Ceccolini M., Giannini M.B., Lucchesi A. (2015). IL-17/IL-10 double-producing T cells: New link between infections, immunosuppression and acute myeloid leukemia. J. Transl. Med..

[292] Han Y., Ye A., Bi L., Wu J., Yu K., Zhang S. (2014). Th17 cells and interleukin-17 increase with poor prognosis in patients with acute myeloid leukemia. Cancer Sci..

[293] Yu S., Liu C., Zhang L., Shan B., Tian T., Hu Y., Shao L., Sun Y., Ji C., Ma D. (2014). Elevated Th22 cells correlated with Th17 cells in peripheral blood of patients with acute myeloid leukemia. Int. J. Mol. Sci..

[294] Derolf A.R., Laane E., Bjorklund E., Saft L., Bjorkholm M., Porwit A. (2014). Dendritic cells in bone marrow at diagnosis and after chemotherapy in adult patients with acute myeloid leukaemia. Scand. J. Immunol..

[295] Sun H., Li Y., Zhang Z.F., Ju Y., Li L., Zhang B.C., Liu B. (2015). Increase in myeloid-derived suppressor cells (MDSCs) associated with minimal residual disease (MRD) detection in adult acute myeloid leukemia. Int. J. Hematol..

[296] Raaijmakers M.H. (2014). Disease progression in myelodysplastic syndromes: Do mesenchymal cells pave the way?. Cell Stem Cell.

[297] Cogle C.R., Saki N., Khodadi E., Li J., Shahjahani M., Azizidoost S. (2015). Bone marrow niche in the myelodysplastic syndromes. Leuk. Res..

[298] Cogle C.R., Bosse R.C., Brewer T., Migdady Y., Shirzad R., Kampen K.R., Saki N. (2015). Acute myeloid leukemia in the vascular niche. Cancer Lett..

[299] Boyd A.L., Campbell C.J., Hopkins C.I., Fiebig-Comyn A., Russell J., Ulemek J., Foley R., Leber B., Xenocostas A., Collins T.J. (2014). Niche displacement of human leukemic stem cells uniquely allows their competitive replacement with healthy HSPCs. J. Exp. Med..

[300] Glait-Santar C., Desmond R., Feng X., Bat T., Chen J., Heuston E., Mizukawa B., Mulloy J.C., Bodine D.M., Larochelle A. (2015). Functional niche competition between normal hematopoietic stem and progenitor cells and myeloid leukemia cells. Stem Cells.

[301] Krause D.S., Scadden D.T., Preffer F.I. (2013). The hematopoietic stem cell niche—Home for friend and foe?. Cytom. B Clin. Cytom..

[302] Walenda T., Stiehl T., Braun H., Frobel J., Ho A.D., Schroeder T., Goecke T.W., Rath B., Germing U., Marciniak-Czochra A. (2014). Feedback signals in myelodysplastic syndromes: Increased self-renewal of the malignant clone suppresses normal hematopoiesis. PLoS Comput. Biol..

[303] Greenbaum A., Hsu Y.M., Day R.B., Schuettpelz L.G., Christopher M.J., Borgerding J.N., Nagasawa T., Link D.C. (2013). CXCL12 in early mesenchymal progenitors is required for haematopoietic stem-cell maintenance. Nature.

[304] Lataillade J.J., Clay D., Bourin P., Herodin F., Dupuy C., Jasmin C., Le Bousse-Kerdiles M.C. (2002). Stromal cell-derived factor 1 regulates primitive hematopoiesis by suppressing apoptosis and by promoting G(0)/G(1) transition in CD34^+^ cells: Evidence for an autocrine/paracrine mechanism. Blood.

[305] Chabanon A., Desterke C., Rodenburger E., Clay D., Guerton B., Boutin L., Bennaceur-Griscelli A., Pierre-Louis O., Uzan G., Abecassis L. (2008). A crosstalk between stromal cell-derived factor-1 and transforming growth factor-β controls the quiescence/cycling switch of CD34^+^ progenitors through FoxO3 and mammalian target of rapamycin. Stem Cells.

[306] Hanoun M., Frenette P.S. (2013). This niche is a maze; an amazing niche. Cell Stem Cell.

[307] Avecilla S.T., Hattori K., Heissig B., Tejada R., Liao F., Shido K., Jin D.K., Dias S., Zhang F., Hartman T.E. (2004). Chemokine-mediated interaction of hematopoietic progenitors with the bone marrow vascular niche is required for thrombopoiesis. Nat. Med..

[308] Wierenga A.T., Vellenga E., Schuringa J.J. (2014). Convergence of hypoxia and TGFbeta pathways on cell cycle regulation in human hematopoietic stem/progenitor cells. PLoS ONE.

[309] Kent D., Copley M., Benz C., Dykstra B., Bowie M., Eaves C. (2008). Regulation of hematopoietic stem cells by the steel factor/KIT signaling pathway. Clin. Cancer Res..

[310] Gong L., Zhao Y., Zhang Y., Ruan Z. (2016). The Macrophage Polarization Regulates MSC Osteoblast Differentiation in vitro. Ann. Clin. Lab. Sci..

[311] Anthony B.A., Link D.C. (2014). Regulation of hematopoietic stem cells by bone marrow stromal cells. Trends Immunol..

[312] Bae M.H., Oh S.H., Park C.J., Lee B.R., Kim Y.J., Cho Y.U., Jang S., Lee J.H., Kim N., Park S.H. (2015). VLA-4 and CXCR4 expression levels show contrasting prognostic impact (favorable and unfavorable, respectively) in acute myeloid leukemia. Ann. Hematol..

[313] Mannelli F., Cutini I., Gianfaldoni G., Bencini S., Scappini B., Pancani F., Ponziani V., Bonetti M.I., Biagiotti C., Longo G. (2014). CXCR4 expression accounts for clinical phenotype and outcome in acute myeloid leukemia. Cytom. B Clin. Cytom..

[314] Rombouts E.J., Pavic B., Lowenberg B., Ploemacher R.E. (2004). Relation between CXCR-4 expression, Flt3 mutations, and unfavorable prognosis of adult acute myeloid leukemia. Blood.

[315] Kuhne M.R., Mulvey T., Belanger B., Chen S., Pan C., Chong C., Cao F., Niekro W., Kempe T., Henning K.A. (2013). BMS-936564/MDX-1338: A fully human anti-CXCR4 antibody induces apoptosis in vitro and shows antitumor activity in vivo in hematologic malignancies. Clin. Cancer Res..

[316] Landry B., Gul-Uludag H., Plianwong S., Kucharski C., Zak Z., Parmar M.B., Kutsch O., Jiang H., Brandwein J., Uludag H. (2016). Targeting CXCR4/SDF-1 axis by lipopolymer complexes of siRNA in acute myeloid leukemia. J. Control. Release.

[317] Li X., Guo H., Yang Y., Meng J., Liu J., Wang C., Xu H. (2014). A designed peptide targeting CXCR4 displays anti-acute myelocytic leukemia activity in vitro and in vivo. Sci. Rep..

[318] Nervi B., Ramirez P., Rettig M.P., Uy G.L., Holt M.S., Ritchey J.K., Prior J.L., Piwnica-Worms D., Bridger G., Ley T.J. (2009). Chemosensitization of acute myeloid leukemia (AML) following mobilization by the CXCR4 antagonist AMD3100. Blood.

[319] Zaitseva L., Murray M.Y., Shafat M.S., Lawes M.J., MacEwan D.J., Bowles K.M., Rushworth S.A. (2014). Ibrutinib inhibits SDF1/CXCR4 mediated migration in AML. Oncotarget.

[320] Weber J.M., Calvi L.M. (2010). Notch signaling and the bone marrow hematopoietic stem cell niche. Bone.

[321] Evans A.G., Calvi L.M. (2015). Notch signaling in the malignant bone marrow microenvironment: Implications for a niche-based model of oncogenesis. Ann. N. Y. Acad. Sci..

[322] Duncan A.W., Rattis F.M., DiMascio L.N., Congdon K.L., Pazianos G., Zhao C., Yoon K., Cook J.M., Willert K., Gaiano N. (2005). Integration of Notch and Wnt signaling in hematopoietic stem cell maintenance. Nat. Immunol..

[323] Li L., Milner L.A., Deng Y., Iwata M., Banta A., Graf L., Marcovina S., Friedman C., Trask B.J., Hood L. (1998). The human homolog of rat Jagged1 expressed by marrow stroma inhibits differentiation of 32D cells through interaction with Notch1. Immunity.

[324] Huang J.C., Basu S.K., Zhao X., Chien S., Fang M., Oehler V.G., Appelbaum F.R., Becker P.S. (2015). Mesenchymal stromal cells derived from acute myeloid leukemia bone marrow exhibit aberrant cytogenetics and cytokine elaboration. Blood Cancer J..

[325] Kim Y.W., Koo B.K., Jeong H.W., Yoon M.J., Song R., Shin J., Jeong D.C., Kim S.H., Kong Y.Y. (2008). Defective Notch activation in microenvironment leads to myeloproliferative disease. Blood.

[326] Wang Y., Krivtsov A.V., Sinha A.U., North T.E., Goessling W., Feng Z., Zon L.I., Armstrong S.A. (2010). The Wnt/beta-catenin pathway is required for the development of leukemia stem cells in AML. Science.

[327] Kannan S., Sutphin R.M., Hall M.G., Golfman L.S., Fang W., Nolo R.M., Akers L.J., Hammitt R.A., McMurray J.S., Kornblau S.M. (2013). Notch activation inhibits AML growth and survival: A potential therapeutic approach. J. Exp. Med..

[328] Kim Y., Thanendrarajan S., Schmidt-Wolf I.G. (2011). Wnt/ss-catenin: A new therapeutic approach to acute myeloid leukemia. Leuk. Res. Treat..

[329] Minke K.S., Staib P., Puetter A., Gehrke I., Gandhirajan R.K., Schlosser A., Schmitt E.K., Hallek M., Kreuzer K.A. (2009). Small molecule inhibitors of WNT signaling effectively induce apoptosis in acute myeloid leukemia cells. Eur. J. Haematol..

[330] Levesque J.P. (2013). A niche in a dish: Pericytes support HSC. Blood.

[331] Kode A., Mosialou I., Manavalan S.J., Rathinam C.V., Friedman R.A., Teruya-Feldstein J., Bhagat G., Berman E., Kousteni S. (2016). FoxO1-dependent induction of acute myeloid leukemia by osteoblasts in mice. Leukemia.

[332] Falconi G., Fabiani E., Fianchi L., Criscuolo M., Raffaelli C.S., Bellesi S., Hohaus S., Voso M.T., D’Alo F., Leone G. (2016). Impairment of PI3K/AKT and WNT/beta-catenin pathways in bone marrow mesenchymal stem cells isolated from patients with myelodysplastic syndromes. Exp. Hematol..

[333] Xu J., Suzuki M., Niwa Y., Hiraga J., Nagasaka T., Ito M., Nakamura S., Tomita A., Abe A., Kiyoi H. (2008). Clinical significance of nuclear non-phosphorylated beta-catenin in acute myeloid leukaemia and myelodysplastic syndrome. Br. J. Haematol..

[334] Wang H., Fan R., Wang X.Q., Wu D.P., Lin G.W., Xu Y., Li W.Y. (2013). Methylation of Wnt antagonist genes: A useful prognostic marker for myelodysplastic syndrome. Ann. Hematol..

[335] Yu J., Cao J., Li H., Liu P., Xu S., Zhou R., Yao Z., Guo X. (2016). Bone marrow fibrosis with fibrocytic and immunoregulatory responses induced by beta-catenin activation in osteoprogenitors. Bone.

[336] Lane S.W., Sykes S.M., Al-Shahrour F., Shterental S., Paktinat M., Lo C.C., Jesneck J.L., Ebert B.L., Williams D.A., Gilliland D.G. (2010). The APC(min) mouse has altered hematopoietic stem cell function and provides a model for MPD/MDS. Blood.

[337] Masala E., Valencia A., Buchi F., Nosi D., Spinelli E., Gozzini A., Sassolini F., Sanna A., Zecchi S., Bosi A. (2012). Hypermethylation of Wnt antagonist gene promoters and activation of Wnt pathway in myelodysplastic marrow cells. Leuk. Res..

[338] Griffiths E.A., Gore S.D., Hooker C., McDevitt M.A., Karp J.E., Smith B.D., Mohammad H.P., Ye Y., Herman J.G., Carraway H.E. (2010). Acute myeloid leukemia is characterized by Wnt pathway inhibitor promoter hypermethylation. Leuk. Lymphoma.

[339] Ghasemi A., Rostami S., Chahardouli B., Alizad G.N., Ghotaslou A., Nadali F. (2015). Study of SFRP1 and SFRP2 methylation status in patients with de novo acute myeloblastic leukemia. Int. J. Hematol. Oncol. Stem Cell Res..

[340] Martin V., Valencia A., Agirre X., Cervera J., San Jose-Eneriz E., Vilas-Zornoza A., Rodriguez-Otero P., Sanz M.A., Herrera C., Torres A. (2010). Epigenetic regulation of the non-canonical Wnt pathway in acute myeloid leukemia. Cancer Sci..

[341] Valencia A., Roman-Gomez J., Cervera J., Such E., Barragan E., Bolufer P., Moscardo F., Sanz G.F., Sanz M.A. (2009). Wnt signaling pathway is epigenetically regulated by methylation of Wnt antagonists in acute myeloid leukemia. Leukemia.

[342] Li K., Hu C., Mei C., Ren Z., Vera J.C., Zhuang Z., Jin J., Tong H. (2014). Sequential combination of decitabine and idarubicin synergistically enhances anti-leukemia effect followed by demethylating Wnt pathway inhibitor promoters and downregulating Wnt pathway nuclear target. J. Transl. Med..

[343] Pleyer L., Greil R. (2015). Digging deep into “dirty” drugs—Modulation of the methylation machinery. Drug Metab. Rev..

[344] Das M., Chatterjee S., Basak P., Das P., Pereira J.A., Dutta R.K., Chaklader M., Chaudhuri S., Law S. (2010). The bone marrow stem stromal imbalance—A key feature of disease progression in case of myelodysplastic mouse model. J. Stem Cells.

[345] Mishima S., Nagai A., Abdullah S., Matsuda C., Taketani T., Kumakura S., Shibata H., Ishikura H., Kim S.U., Masuda J. (2010). Effective ex vivo expansion of hematopoietic stem cells using osteoblast-differentiated mesenchymal stem cells is CXCL12 dependent. Eur. J. Haematol..

[346] Oh I.H., Kwon K.R. (2010). Concise review: Multiple niches for hematopoietic stem cell regulations. Stem Cells.

[347] Koh S.H., Choi H.S., Park E.S., Kang H.J., Ahn H.S., Shin H.Y. (2005). Co-culture of human CD34^+^ cells with mesenchymal stem cells increases the survival of CD34^+^ cells against the 5-aza-deoxycytidine- or trichostatin A-induced cell death. Biochem. Biophys. Res. Commun..

[348] Jing D., Fonseca A.V., Alakel N., Fierro F.A., Muller K., Bornhauser M., Ehninger G., Corbeil D., Ordemann R. (2010). Hematopoietic stem cells in co-culture with mesenchymal stromal cells—Modeling the niche compartments in vitro. Haematologica.

[349] Nakamura Y., Arai F., Iwasaki H., Hosokawa K., Kobayashi I., Gomei Y., Matsumoto Y., Yoshihara H., Suda T. (2010). Isolation and characterization of endosteal niche cell populations that regulate hematopoietic stem cells. Blood.

[350] Semerad C.L., Christopher M.J., Liu F., Short B., Simmons P.J., Winkler I., Levesque J.P., Chappel J., Ross F.P., Link D.C. (2005). G-CSF potently inhibits osteoblast activity and CXCL12 mRNA expression in the bone marrow. Blood.

[351] Taichman R.S., Emerson S.G. (1994). Human osteoblasts support hematopoiesis through the production of granulocyte colony-stimulating factor. J. Exp. Med..

[352] Visnjic D., Kalajzic Z., Rowe D.W., Katavic V., Lorenzo J., Aguila H.L. (2004). Hematopoiesis is severely altered in mice with an induced osteoblast deficiency. Blood.

[353] Nagasawa T. (2008). New niches for B cells. Nat. Immunol..

[354] Nagasawa T. (2007). The chemokine CXCL12 and regulation of HSC and B lymphocyte development in the bone marrow niche. Adv. Exp. Med. Biol..

[355] Van Pel M., Fibbe W.E., Schepers K. (2015). The human and murine hematopoietic stem cell niches: Are they comparable?. Ann. N. Y. Acad. Sci..

[356] Iancu-Rubin C., Mosoyan G., Wang J., Kraus T., Sung V., Hoffman R. (2013). Stromal cell-mediated inhibition of erythropoiesis can be attenuated by Sotatercept (ACE-011), an activin receptor type II ligand trap. Exp. Hematol..

[357] Lazar-Karsten P., Dorn I., Meyer G., Lindner U., Driller B., Schlenke P. (2011). The influence of extracellular matrix proteins and mesenchymal stem cells on erythropoietic cell maturation. Vox Sang..

[358] Chou D.B., Sworder B., Bouladoux N., Roy C.N., Uchida A.M., Grigg M., Robey P.G., Belkaid Y. (2012). Stromal-derived IL-6 alters the balance of myeloerythroid progenitors during Toxoplasma gondii infection. J. Leukoc. Biol..

[359] Sanchez-Correa B., Bergua J.M., Campos C., Gayoso I., Arcos M.J., Banas H., Morgado S., Casado J.G., Solana R., Tarazona R. (2013). Cytokine profiles in acute myeloid leukemia patients at diagnosis: Survival is inversely correlated with IL-6 and directly correlated with IL-10 levels. Cytokine.

[360] Shao L., Frigon N.L., Young A.L., Yu A.L., Mathews L.S., Vaughan J., Vale W., Yu J. (1992). Effect of activin A on globin gene expression in purified human erythroid progenitors. Blood.

[361] Shav-Tal Y., Zipori D. (2002). The role of activin a in regulation of hemopoiesis. Stem Cells.

[362] Yu J., Shao L., Vaughan J., Vale W., Yu A.L. (1989). Characterization of the potentiation effect of activin on human erythroid colony formation in vitro. Blood.

[363] Gibson D.P., DeGowin R.L., Knapp S.A. (1982). Effect of X irradiation on release of prostaglandin E from marrow stromal cells in culture. Radiat. Res..

[364] Nocka K.H., Ottman O.G., Pelus L.M. (1989). The role of marrow accessory cell populations in the augmentation of human erythroid progenitor cell (BFU-E) proliferation by prostaglandin E. Leuk. Res..

[365] DeGowin R.L., Gibson D.P. (1981). Prostaglandin-mediated enhancement of erythroid colonies by marrow stromal cells (MSC). Exp. Hematol..

[366] Werts E.D., DeGowin R.L., Knapp S.K., Gibson D.P. (1980). Characterization of marrow stromal (fibroblastoid) cells and their association with erythropoiesis. Exp. Hematol..

[367] Arai F., Hirao A., Ohmura M., Sato H., Matsuoka S., Takubo K., Ito K., Koh G.Y., Suda T. (2004). Tie2/angiopoietin-1 signaling regulates hematopoietic stem cell quiescence in the bone marrow niche. Cell.

[368] Stier S., Ko Y., Forkert R., Lutz C., Neuhaus T., Grunewald E., Cheng T., Dombkowski D., Calvi L.M., Rittling S.R. (2005). Osteopontin is a hematopoietic stem cell niche component that negatively regulates stem cell pool size. J. Exp. Med..

[369] Marie P.J., Hay E., Saidak Z. (2014). Integrin and cadherin signaling in bone: Role and potential therapeutic targets. Trends Endocrinol. Metab..

[370] Huber B.C., Grabmaier U., Brunner S. (2014). Impact of parathyroid hormone on bone marrow-derived stem cell mobilization and migration. World J. Stem Cells.

[371] Krause D.S., Fulzele K., Catic A., Sun C.C., Dombkowski D., Hurley M.P., Lezeau S., Attar E., Wu J.Y., Lin H.Y. (2013). Differential regulation of myeloid leukemias by the bone marrow microenvironment. Nat. Med..

[372] Ishikawa F., Yoshida S., Saito Y., Hijikata A., Kitamura H., Tanaka S., Nakamura R., Tanaka T., Tomiyama H., Saito N. (2007). Chemotherapy-resistant human AML stem cells home to and engraft within the bone-marrow endosteal region. Nat. Biotechnol..

[373] Fulzele K., Krause D.S., Panaroni C., Saini V., Barry K.J., Liu X., Lotinun S., Baron R., Bonewald L., Feng J.Q. (2013). Myelopoiesis is regulated by osteocytes through Gsalpha-dependent signaling. Blood.

[374] Choi S.T., Kim J.H., Kang E.J., Lee S.W., Park M.C., Park Y.B., Lee S.K. (2008). Osteopontin might be involved in bone remodelling rather than in inflammation in ankylosing spondylitis. Rheumatology.

[375] Reinholt F.P., Hultenby K., Oldberg A., Heinegard D. (1990). Osteopontin—A possible anchor of osteoclasts to bone. PNAS.

[376] Wang K.X., Denhardt D.T. (2008). Osteopontin: Role in immune regulation and stress responses. Cytokine Growth Factor Rev..

[377] Nilsson S.K., Johnston H.M., Whitty G.A., Williams B., Webb R.J., Denhardt D.T., Bertoncello I., Bendall L.J., Simmons P.J., Haylock D.N. (2005). Osteopontin, a key component of the hematopoietic stem cell niche and regulator of primitive hematopoietic progenitor cells. Blood.

[378] Powell J.A., Thomas D., Barry E.F., Kok C.H., McClure B.J., Tsykin A., To L.B., Brown A., Lewis I.D., Herbert K. (2009). Expression profiling of a hemopoietic cell survival transcriptome implicates osteopontin as a functional prognostic factor in AML. Blood.

[379] Frisch B.J., Ashton J.M., Xing L., Becker M.W., Jordan C.T., Calvi L.M. (2012). Functional inhibition of osteoblastic cells in an in vivo mouse model of myeloid leukemia. Blood.

[380] Liersch R., Gerss J., Schliemann C., Bayer M., Schwoppe C., Biermann C., Appelmann I., Kessler T., Lowenberg B., Buchner T. (2012). Osteopontin is a prognostic factor for survival of acute myeloid leukemia patients. Blood.

[381] Ludin A., Itkin T., Gur-Cohen S., Mildner A., Shezen E., Golan K., Kollet O., Kalinkovich A., Porat Z., D’Uva G. (2012). Monocytes-macrophages that express alpha-smooth muscle actin preserve primitive hematopoietic cells in the bone marrow. Nat. Immunol..

[382] Alakel N., Jing D., Muller K., Bornhauser M., Ehninger G., Ordemann R. (2009). Direct contact with mesenchymal stromal cells affects migratory behavior and gene expression profile of CD133^+^ hematopoietic stem cells during ex vivo expansion. Exp. Hematol..

[383] Uccelli A., Pistoia V., Moretta L. (2007). Mesenchymal stem cells: A new strategy for immunosuppression?. Trends Immunol..

[384] Zhao S., Wehner R., Bornhauser M., Wassmuth R., Bachmann M., Schmitz M. (2010). Immunomodulatory properties of mesenchymal stromal cells and their therapeutic consequences for immune-mediated disorders. Stem Cells Dev..

[385] Mattar P., Bieback K. (2015). Comparing the immunomodulatory properties of bone marrow, adipose tissue, and birth-associated tissue mesenchymal stromal vells. Front. Immunol..

[386] Wolf D., Wolf A.M. (2008). Mesenchymal stem cells as cellular immunosuppressants. Lancet.

[387] Najar M., Raicevic G., Crompot E., Fayyad-Kazan H., Bron D., Toungouz M., Lagneaux L. (2016). The immunomodulatory potential of mesenchymal stromal cells: A story of a regulatory network. J. Immunother..

[388] Najar M., Raicevic G., Fayyad-Kazan H., Bron D., Toungouz M., Lagneaux L. (2016). Mesenchymal stromal cells and immunomodulation: A gathering of regulatory immune cells. Cytotherapy.

[389] Haddad R., Saldanha-Araujo F. (2014). Mechanisms of T-cell immunosuppression by mesenchymal stromal cells: What do we know so far?. BioMed Res. Int..

[390] Jiang X.X., Zhang Y., Li X.S., Wu Y., Yu X.D., Tang P.H., Mao N. (2005). Osteoblasts derived from mesenchymal stem cells harbor immunoregulatory effect. Zhongguo Shi Yan Xue Ye Xue Za Zhi.

[391] Liu H., Kemeny D.M., Heng B.C., Ouyang H.W., Melendez A.J., Cao T. (2006). The immunogenicity and immunomodulatory function of osteogenic cells differentiated from mesenchymal stem cells. J. Immunol..

[392] Le Blanc K., Tammik C., Rosendahl K., Zetterberg E., Ringden O. (2003). HLA expression and immunologic properties of differentiated and undifferentiated mesenchymal stem cells. Exp. Hematol..

[393] radier A., Passweg J., Villard J., Kindler V. (2011). Human bone marrow stromal cells and skin fibroblasts inhibit natural killer cell proliferation and cytotoxic activity. Cell Transplant..

[394] Haniffa M.A., Wang X.N., Holtick U., Rae M., Isaacs J.D., Dickinson A.M., Hilkens C.M., Collin M.P. (2007). Adult human fibroblasts are potent immunoregulatory cells and functionally equivalent to mesenchymal stem cells. J. Immunol..

[395] Poloni A., Maurizi G., Ciarlantini M., Medici M., Mattiucci D., Mancini S., Maurizi A., Falconi M., Olivieri A., Leoni P. (2015). Interaction between human mature adipocytes and lymphocytes induces T-cell proliferation. Cytotherapy.

[396] Hoogduijn M.J. (2015). Are mesenchymal stromal cells immune cells?. Arthritis Res. Ther..

[397] Romieu-Mourez R., Coutu D.L., Galipeau J. (2012). The immune plasticity of mesenchymal stromal cells from mice and men: Concordances and discrepancies. Front. Biosci..

[398] Chinnadurai R., Ng S., Velu V., Galipeau J. (2015). Challenges in animal modelling of mesenchymal stromal cell therapy for inflammatory bowel disease. World J. Gastroenterol..

[399] Ren G., Su J., Zhang L., Zhao X., Ling W., L’huillie A., Zhang J., Lu Y., Roberts A.I., Ji W. (2009). Species variation in the mechanisms of mesenchymal stem cell-mediated immunosuppression. Stem Cells.

[400] Schurgers E., Kelchtermans H., Mitera T., Geboes L., Matthys P. (2010). Discrepancy between the in vitro and in vivo effects of murine mesenchymal stem cells on T-cell proliferation and collagen-induced arthritis. Arthritis Res. Ther..

[401] Romieu-Mourez R., Francois M., Boivin M.N., Stagg J., Galipeau J. (2007). Regulation of MHC class II expression and antigen processing in murine and human mesenchymal stromal cells by IFN-gamma, TGF-beta, and cell density. J. Immunol..

[402] Galipeau J., Krampera M., Barrett J., Dazzi F., Deans R.J., DeBruijn J., Dominici M., Fibbe W.E., Gee A.P., Gimble J.M. (2016). International Society for Cellular Therapy perspective on immune functional assays for mesenchymal stromal cells as potency release criterion for advanced phase clinical trials. Cytotherapy.

[403] Di Nicola M., Carlo-Stella C., Magni M., Milanesi M., Longoni P.D., Matteucci P., Grisanti S., Gianni A.M. (2002). Human bone marrow stromal cells suppress T-lymphocyte proliferation induced by cellular or nonspecific mitogenic stimuli. Blood.

[404] Duffy M.M., Ritter T., Ceredig R., Griffin M.D. (2011). Mesenchymal stem cell effects on T-cell effector pathways. Stem Cell Res. Ther..

[405] Engela A.U., Baan C.C., Litjens N.H., Franquesa M., Betjes M.G., Weimar W., Hoogduijn M.J. (2013). Mesenchymal stem cells control alloreactive CD8^+^ CD28^−^ T cells. Clin. Exp. Immunol..

[406] Krampera M., Glennie S., Dyson J., Scott D., Laylor R., Simpson E., Dazzi F. (2003). Bone marrow mesenchymal stem cells inhibit the response of naive and memory antigen-specific T cells to their cognate peptide. Blood.

[407] Li M., Sun X., Kuang X., Liao Y., Li H., Luo D. (2014). Mesenchymal stem cells suppress CD8^+^ T cell-mediated activation by suppressing natural killer group 2, member D protein receptor expression and secretion of prostaglandin E2, indoleamine 2,3-dioxygenase and transforming growth factor-beta. Clin. Exp. Immunol..

[408] Ribeiro A., Laranjeira P., Mendes S., Velada I., Leite C., Andrade P., Santos F., Henriques A., Graos M., Cardoso C.M. (2013). Mesenchymal stem cells from umbilical cord matrix, adipose tissue and bone marrow exhibit different capability to suppress peripheral blood B, natural killer and T cells. Stem Cell Res. Ther..

[409] Sato K., Ozaki K., Oh I., Meguro A., Hatanaka K., Nagai T., Muroi K., Ozawa K. (2007). Nitric oxide plays a critical role in suppression of T-cell proliferation by mesenchymal stem cells. Blood.

[410] Ghannam S., Pene J., Moquet-Torcy G., Jorgensen C., Yssel H. (2010). Mesenchymal stem cells inhibit human Th17 cell differentiation and function and induce a T regulatory cell phenotype. J. Immunol..

[411] Luz-Crawford P., Noel D., Fernandez X., Khoury M., Figueroa F., Carrion F., Jorgensen C., Djouad F. (2012). Mesenchymal stem cells repress Th17 molecular program through the PD-1 pathway. PLoS ONE.

[412] Luz-Crawford P., Kurte M., Bravo-Alegria J., Contreras R., Nova-Lamperti E., Tejedor G., Noel D., Jorgensen C., Figueroa F., Djouad F. (2013). Mesenchymal stem cells generate a CD4^+^CD25^+^Foxp3^+^ regulatory T cell population during the differentiation process of Th1 and Th17 cells. Stem Cell Res. Ther..

[413] Luz-Crawford P., Djouad F., Toupet K., Bony C., Franquesa M., Hoogduijn M.J., Jorgensen C., Noel D. (2015). Mesenchymal stem cell-derived interleukin I receptor antagonist promotes macrophage polarization and inhibits B cell differentiation. Stem Cells.

[414] Martinet L., Fleury-Cappellesso S., Gadelorge M., Dietrich G., Bourin P., Fournie J.J., Poupot R. (2009). A regulatory crosstalk between Vgamma9Vdelta2 T lymphocytes and mesenchymal stem cells. Eur. J. Immunol..

[415] Petrini I., Pacini S., Petrini M., Fazzi R., Trombi L., Galimberti S. (2009). Mesenchymal cells inhibit expansion but not cytotoxicity exerted by gamma-delta T cells. Eur. J. Clin. Investig..

[416] Prigione I., Benvenuto F., Bocca P., Battistini L., Uccelli A., Pistoia V. (2009). Reciprocal interactions between human mesenchymal stem cells and gammadelta T cells or invariant natural killer T cells. Stem Cells.

[417] Spaggiari G.M., Capobianco A., Abdelrazik H., Becchetti F., Mingari M.C., Moretta L. (2008). Mesenchymal stem cells inhibit natural killer-cell proliferation, cytotoxicity, and cytokine production: Role of indoleamine 2,3-dioxygenase and prostaglandin E2. Blood.

[418] Li Y., Qu Y.H., Wu Y.F., Liu L., Lin X.H., Huang K., Wei J. (2015). Bone marrow mesenchymal stem cells suppressing activation of allogeneic cytokine-induced killer/natural killer cells either by direct or indirect interaction. Cell Biol. Int..

[419] Sotiropoulou P.A., Perez S.A., Gritzapis A.D., Baxevanis C.N., Papamichail M. (2006). Interactions between human mesenchymal stem cells and natural killer cells. Stem Cells.

[420] Spaggiari G.M., Capobianco A., Becchetti S., Mingari M.C., Moretta L. (2006). Mesenchymal stem cell-natural killer cell interactions: Evidence that activated NK cells are capable of killing MSCs, whereas MSCs can inhibit IL-2-induced NK-cell proliferation. Blood.

[421] Chiesa S., Morbelli S., Morando S., Massollo M., Marini C., Bertoni A., Frassoni F., Bartolome S.T., Sambuceti G., Traggiai E. (2011). Mesenchymal stem cells impair in vivo T-cell priming by dendritic cells. PNAS.

[422] Laranjeira P., Gomes J., Pedreiro S., Pedrosa M., Martinho A., Antunes B., Ribeiro T., Santos F., Domingues R., Abecasis M. (2015). Human bone marrow-derived mesenchymal stromal cells differentially inhibit cytokine production by peripheral blood monocytes subpopulations and myeloid dendritic cells. Stem Cells Int..

[423] Nauta A.J., Kruisselbrink A.B., Lurvink E., Willemze R., Fibbe W.E. (2006). Mesenchymal stem cells inhibit generation and function of both CD34^+^-derived and monocyte-derived dendritic cells. J. Immunol..

[424] Ramasamy R., Fazekasova H., Lam E.W., Soeiro I., Lombardi G., Dazzi F. (2007). Mesenchymal stem cells inhibit dendritic cell differentiation and function by preventing entry into the cell cycle. Transplantation.

[425] Spaggiari G.M., Abdelrazik H., Becchetti F., Moretta L. (2009). MSCs inhibit monocyte-derived DC maturation and function by selectively interfering with the generation of immature DCs: Central role of MSC-derived prostaglandin E2. Blood.

[426] Spaggiari G.M., Moretta L. (2013). Interactions between mesenchymal stem cells and dendritic cells. Adv. Biochem. Eng. Biotechnol..

[427] Zhang B., Liu R., Shi D., Liu X., Chen Y., Dou X., Zhu X., Lu C., Liang W., Liao L. (2009). Mesenchymal stem cells induce mature dendritic cells into a novel Jagged-2-dependent regulatory dendritic cell population. Blood.

[428] Wehner R., Wehrum D., Bornhauser M., Zhao S., Schakel K., Bachmann M.P., Platzbecker U., Ehninger G., Rieber E.P., Schmitz M. (2009). Mesenchymal stem cells efficiently inhibit the proinflammatory properties of 6-sulfo LacNAc dendritic cells. Haematologica.

[429] Che N., Li X., Zhou S., Liu R., Shi D., Lu L., Sun L. (2012). Umbilical cord mesenchymal stem cells suppress B-cell proliferation and differentiation. Cell Immunol..

[430] Rosado M.M., Bernardo M.E., Scarsella M., Conforti A., Giorda E., Biagini S., Cascioli S., Rossi F., Guzzo I., Vivarelli M. (2015). Inhibition of B-cell proliferation and antibody production by mesenchymal stromal cells is mediated by T cells. Stem Cells Dev..

[431] Asari S., Itakura S., Ferreri K., Liu C.P., Kuroda Y., Kandeel F., Mullen Y. (2009). Mesenchymal stem cells suppress B-cell terminal differentiation. Exp. Hematol..

[432] Corcione A., Benvenuto F., Ferretti E., Giunti D., Cappiello V., Cazzanti F., Risso M., Gualandi F., Mancardi G.L., Pistoia V. (2006). Human mesenchymal stem cells modulate B-cell functions. Blood.

[433] Franquesa M., Hoogduijn M.J., Bestard O., Grinyo J.M. (2012). Immunomodulatory effect of mesenchymal stem cells on B cells. Front. Immunol..

[434] Aldinucci A., Rizzetto L., Pieri L., Nosi D., Romagnoli P., Biagioli T., Mazzanti B., Saccardi R., Beltrame L., Massacesi L. (2010). Inhibition of immune synapse by altered dendritic cell actin distribution: A new pathway of mesenchymal stem cell immune regulation. J. Immunol..

[435] Franquesa M., Mensah F.K., Huizinga R., Strini T., Boon L., Lombardo E., DelaRosa O., Laman J.D., Grinyo J.M., Weimar W. (2015). Human adipose tissue-derived mesenchymal stem cells abrogate plasmablast formation and induce regulatory B cells independently of T helper cells. Stem Cells.

[436] Raffaghello L., Bianchi G., Bertolotto M., Montecucco F., Busca A., Dallegri F., Ottonello L., Pistoia V. (2008). Human mesenchymal stem cells inhibit neutrophil apoptosis: A model for neutrophil preservation in the bone marrow niche. Stem Cells.

[437] Cahill E.F., Tobin L.M., Carty F., Mahon B.P., English K. (2015). Jagged-1 is required for the expansion of CD4^+^ CD25^+^ FoxP3^+^ regulatory T cells and tolerogenic dendritic cells by murine mesenchymal stromal cells. Stem Cell Res. Ther..

[438] Castro-Manrreza M.E., Mayani H., Monroy-Garcia A., Flores-Figueroa E., Chavez-Rueda K., Legorreta-Haquet V., Santiago-Osorio E., Montesinos J.J. (2014). Human mesenchymal stromal cells from adult and neonatal sources: A comparative in vitro analysis of their immunosuppressive properties against T cells. Stem Cells Dev..

[439] Crop M.J., Baan C.C., Korevaar S.S., Ijzermans J.N., Weimar W., Hoogduijn M.J. (2010). Human adipose tissue-derived mesenchymal stem cells induce explosive T-cell proliferation. Stem Cells Dev..

[440] Di Ianni M., Del Papa B., De Ioanni M., Moretti L., Bonifacio E., Cecchini D., Sportoletti P., Falzetti F., Tabilio A. (2008). Mesenchymal cells recruit and regulate T regulatory cells. Exp. Hematol..

[441] Engela A.U., Baan C.C., Peeters A.M., Weimar W., Hoogduijn M.J. (2013). Interaction between adipose tissue-derived mesenchymal stem cells and regulatory T-cells. Cell Transplant..

[442] Engela A.U., Hoogduijn M.J., Boer K., Litjens N.H., Betjes M.G., Weimar W., Baan C.C. (2013). Human adipose-tissue derived mesenchymal stem cells induce functional de-novo regulatory T cells with methylated FOXP3 gene DNA. Clin. Exp. Immunol..

[443] Mougiakakos D., Jitschin R., Johansson C.C., Okita R., Kiessling R., Le Blanc K. (2011). The impact of inflammatory licensing on heme oxygenase-1-mediated induction of regulatory T cells by human mesenchymal stem cells. Blood.

[444] Prevosto C., Zancolli M., Canevali P., Zocchi M.R., Poggi A. (2007). Generation of CD4^+^ or CD8^+^ regulatory T cells upon mesenchymal stem cell-lymphocyte interaction. Haematologica.

[445] Yang N., Baban B., Isales C.M., Shi X.M. (2015). Crosstalk between bone marrow-derived mesenchymal stem cells and regulatory T cells through a glucocorticoid-induced leucine zipper/developmental endothelial locus-1-dependent mechanism. FASEB J..

[446] Liu Q., Zheng H., Chen X., Peng Y., Huang W., Li X., Li G., Xia W., Sun Q., Xiang A.P. (2015). Human mesenchymal stromal cells enhance the immunomodulatory function of CD8^+^CD28^−^ regulatory T cells. Cell Mol. Immunol..

[447] Deng Y., Yi S., Wang G., Cheng J., Zhang Y., Chen W., Tai Y., Chen S., Chen G., Liu W. (2014). Umbilical cord-derived mesenchymal stem cells instruct dendritic cells to acquire tolerogenic phenotypes through the IL-6-mediated upregulation of SOCS1. Stem Cells Dev..

[448] Li Y.P., Paczesny S., Lauret E., Poirault S., Bordigoni P., Mekhloufi F., Hequet O., Bertrand Y., Ou-Yang J.P., Stoltz J.F. (2008). Human mesenchymal stem cells license adult CD34^+^ hemopoietic progenitor cells to differentiate into regulatory dendritic cells through activation of the Notch pathway. J. Immunol..

[449] Zhao Z.G., Xu W., Sun L., You Y., Li F., Li Q.B., Zou P. (2012). Immunomodulatory function of regulatory dendritic cells induced by mesenchymal stem cells. Immunol. Investig..

[450] Nemeth K., Leelahavanichkul A., Yuen P.S., Mayer B., Parmelee A., Doi K., Robey P.G., Leelahavanichkul K., Koller B.H., Brown J.M. (2009). Bone marrow stromal cells attenuate sepsis via prostaglandin E(2)-dependent reprogramming of host macrophages to increase their interleukin-10 production. Nat. Med..

[451] Xie Z., Hao H., Tong C., Cheng Y., Liu J., Pang Y., Si Y., Guo Y., Zang L., Mu Y. (2015). Human umbilical cord-derived mesenchymal stem cells elicit macrophages into an anti-inflammatory phenotype to alleviate insulin resistance in type 2 diabetic rats. Stem Cells.

[452] Barminko J.A., Nativ N.I., Schloss R., Yarmush M.L. (2014). Fractional factorial design to investigate stromal cell regulation of macrophage plasticity. Biotechnol. Bioeng..

[453] Cho D.I., Kim M.R., Jeong H.Y., Jeong H.C., Jeong M.H., Yoon S.H., Kim Y.S., Ahn Y. (2014). Mesenchymal stem cells reciprocally regulate the M1/M2 balance in mouse bone marrow-derived macrophages. Exp. Mol. Med..

[454] Francois M., Romieu-Mourez R., Li M., Galipeau J. (2012). Human MSC suppression correlates with cytokine induction of indoleamine 2,3-dioxygenase and bystander M2 macrophage differentiation. Mol. Ther..

[455] Beyth S., Borovsky Z., Mevorach D., Liebergall M., Gazit Z., Aslan H., Galun E., Rachmilewitz J. (2005). Human mesenchymal stem cells alter antigen-presenting cell maturation and induce T-cell unresponsiveness. Blood.

[456] Gur-Wahnon D., Borovsky Z., Beyth S., Liebergall M., Rachmilewitz J. (2007). Contact-dependent induction of regulatory antigen-presenting cells by human mesenchymal stem cells is mediated via STAT3 signaling. Exp. Hematol..

[457] Yen B.L., Yen M.L., Hsu P.J., Liu K.J., Wang C.J., Bai C.H., Sytwu H.K. (2013). Multipotent human mesenchymal stromal cells mediate expansion of myeloid-derived suppressor cells via hepatocyte growth factor/c-Met and STAT3. Stem Cell Rep..

[458] Chen H.W., Chen H.Y., Wang L.T., Wang F.H., Fang L.W., Lai H.Y., Chen H.H., Lu J., Hung M.S., Cheng Y. (2013). Mesenchymal stem cells tune the development of monocyte-derived dendritic cells toward a myeloid-derived suppressive phenotype through growth-regulated oncogene chemokines. J. Immunol..

[459] Guo Y., Chan K.H., Lai W.H., Siu C.W., Kwan S.C., Tse H.F., Wing-Lok H.P., Wing-Man H.J. (2013). Human mesenchymal stem cells upregulate CD1d^high^CD5^+^ regulatory B cells in experimental autoimmune encephalomyelitis. Neuroimmunomodulation.

[460] in Y., Zhou Z., Zhang F., Wang Y., Shen B., Liu Y., Guo Y., Fan Y., Qiu J. (2015). Induction of regulatory B-cells by mesenchymal stem cells is affected by SDF-1alpha-CXCR7. Cell. Physiol. Biochem..

[461] Burr S.P., Dazzi F., Garden O.A. (2013). Mesenchymal stromal cells and regulatory T cells: The Yin and Yang of peripheral tolerance?. Immunol. Cell Biol..

[462] Bruno S., Deregibus M.C., Camussi G. (2015). The secretome of mesenchymal stromal cells: Role of extracellular vesicles in immunomodulation. Immunol. Lett..

[463] Ren G., Zhang L., Zhao X., Xu G., Zhang Y., Roberts A.I., Zhao R.C., Shi Y. (2008). Mesenchymal stem cell-mediated immunosuppression occurs via concerted action of chemokines and nitric oxide. Cell Stem Cell.

[464] Fontaine M.J., Shih H., Schafer R., Pittenger M.F. (2016). Unraveling the mesenchymal stromal cells’ paracrine immunomodulatory effects. Transfus. Med. Rev..

[465] Hoogduijn M.J., Popp F., Verbeek R., Masoodi M., Nicolaou A., Baan C., Dahlke M.H. (2010). The immunomodulatory properties of mesenchymal stem cells and their use for immunotherapy. Int. Immunopharmacol..

[466] Del Fattore A., Luciano R., Pascucci L., Goffredo B.M., Giorda E., Scapaticci M., Fierabracci A., Muraca M. (2015). Immunoregulatory effects of mesenchymal stem cell-derived extracellular vesicles on T lymphocytes. Cell Transplant..

[467] Groh M.E., Maitra B., Szekely E., Koc O.N. (2005). Human mesenchymal stem cells require monocyte-mediated activation to suppress alloreactive T cells. Exp. Hematol..

[468] Meisel R., Brockers S., Heseler K., Degistirici O., Bülle H., Woite C., Stuhlsatz S., Schwippert W., Jager M., Sorg R. (2011). Human but not murine multipotent mesenchymal stromal cells exhibit broad-spectrum antimicrobial effector function mediated by indoleamine 2,3-dioxygenase. Leukemia.

[469] English K., Ryan J.M., Tobin L., Murphy M.J., Barry F.P., Mahon B.P. (2009). Cell contact, prostaglandin E(2) and transforming growth factor beta 1 play non-redundant roles in human mesenchymal stem cell induction of CD4^+^CD25(High) forkhead box P3^+^ regulatory T cells. Clin. Exp. Immunol..

[470] Ren G., Zhao X., Zhang L., Zhang J., L’Huillier A., Ling W., Roberts A.I., Le A.D., Shi S., Shao C. (2010). Inflammatory cytokine-induced intercellular adhesion molecule-1 and vascular cell adhesion molecule-1 in mesenchymal stem cells are critical for immunosuppression. J. Immunol..

[471] Zoso A., Mazza E.M., Bicciato S., Mandruzzato S., Bronte V., Serafini P., Inverardi L. (2014). Human fibrocytic myeloid-derived suppressor cells express IDO and promote tolerance via Treg-cell expansion. Eur. J. Immunol..

[472] Yu J., Du W., Yan F., Wang Y., Li H., Cao S., Yu W., Shen C., Liu J., Ren X. (2013). Myeloid-derived suppressor cells suppress antitumor immune responses through IDO expression and correlate with lymph node metastasis in patients with breast cancer. J. Immunol..

[473] Koorella C., Nair J.R., Murray M.E., Carlson L.M., Watkins S.K., Lee K.P. (2014). Novel regulation of CD80/CD86-induced phosphatidylinositol 3-kinase signaling by NOTCH1 protein in interleukin-6 and indoleamine 2,3-dioxygenase production by dendritic cells. J. Biol. Chem..

[474] Harden J.L., Egilmez N.K. (2012). Indoleamine 2,3-dioxygenase and dendritic cell tolerogenicity. Immunol. Investig..

[475] Choi H., Lee R.H., Bazhanov N., Oh J.Y., Prockop D.J. (2011). Anti-inflammatory protein TSG-6 secreted by activated MSCs attenuates zymosan-induced mouse peritonitis by decreasing TLR2/NF-kappaB signaling in resident macrophages. Blood.

[476] Melief S.M., Geutskens S.B., Fibbe W.E., Roelofs H. (2013). Multipotent stromal cells skew monocytes towards an anti-inflammatory interleukin-10-producing phenotype by production of interleukin-6. Haematologica.

[477] Melief S.M., Schrama E., Brugman M.H., Tiemessen M.M., Hoogduijn M.J., Fibbe W.E., Roelofs H. (2013). Multipotent stromal cells induce human regulatory T cells through a novel pathway involving skewing of monocytes toward anti-inflammatory macrophages. Stem Cells.

[478] Mantovani A., Biswas S.K., Galdiero M.R., Sica A., Locati M. (2013). Macrophage plasticity and polarization in tissue repair and remodelling. J. Pathol..

[479] Glennie S., Soeiro I., Dyson P.J., Lam E.W., Dazzi F. (2005). Bone marrow mesenchymal stem cells induce division arrest anergy of activated T cells. Blood.

[480] Wobus M., Benath G., Ferrer R.A., Wehner R., Schmitz M., Hofbauer L.C., Rauner M., Ehninger G., Bornhäuser M., Platzbecker U. (2012). Impact of lenalidomide on the functional properties of human mesenchymal stromal cells. Exp. Hematol..

[481] Tatara R., Ozaki K., Kikuchi Y., Hatanaka K., Oh I., Meguro A., Matsu H., Sato K., Ozawa K. (2011). Mesenchymal stromal cells inhibit Th17 but not regulatory T-cell differentiation. Cytotherapy.

[482] Jiang X.X., Zhang Y., Liu B., Zhang S.X., Wu Y., Yu X.D., Mao N. (2005). Human mesenchymal stem cells inhibit differentiation and function of monocyte-derived dendritic cells. Blood.

[483] Zhi-Gang Z., Wei-Ming L., Zhi-Chao C., Yong Y., Ping Z. (2008). Immunosuppressive properties of mesenchymal stem cells derived from bone marrow of patient with hematological malignant diseases. Leuk. Lymphoma.

[484] Djouad F., Charbonnier L.M., Bouffi C., Louis-Plence P., Bony C., Apparailly F., Cantos C., Jorgensen C., Noel D. (2007). Mesenchymal stem cells inhibit the differentiation of dendritic cells through an interleukin-6-dependent mechanism. Stem Cells.

[485] Zhang W., Ge W., Li C., You S., Liao L., Han Q., Deng W., Zhao R.C. (2004). Effects of mesenchymal stem cells on differentiation, maturation, and function of human monocyte-derived dendritic cells. Stem Cells Dev..

[486] Peng Y., Chen X., Liu Q., Xu D., Zheng H., Liu L., Liu Q., Liu M., Fan Z., Sun J. (2014). Alteration of naive and memory B-cell subset in chronic graft-versus-host disease patients after treatment with mesenchymal stromal cells. Stem Cells Transl. Med..

[487] Romieu-Mourez R., Francois M., Boivin M.N., Bouchentouf M., Spaner D.E., Galipeau J. (2009). Cytokine modulation of TLR expression and activation in mesenchymal stromal cells leads to a proinflammatory phenotype. J. Immunol..

[488] Wang Y., Chen X., Cao W., Shi Y. (2014). Plasticity of mesenchymal stem cells in immunomodulation: Pathological and therapeutic implications. Nat. Immunol..

[489] Majumdar M.K., Keane-Moore M., Buyaner D., Hardy W.B., Moorman M.A., McIntosh K.R., Mosca J.D. (2003). Characterization and functionality of cell surface molecules on human mesenchymal stem cells. J. Biomed. Sci..

[490] Quaedackers M.E., Baan C.C., Weimar W., Hoogduijn M.J. (2009). Cell contact interaction between adipose-derived stromal cells and allo-activated T lymphocytes. Eur. J. Immunol..

[491] Regateiro F.S., Cobbold S.P., Waldmann H. (2013). CD73 and adenosine generation in the creation of regulatory microenvironments. Clin. Exp. Immunol..

[492] Munn D.H., Mellor A.L. (2013). Indoleamine 2,3 dioxygenase and metabolic control of immune responses. Trends Immunol..

[493] Fallarino F., Grohmann U., Vacca C., Bianchi R., Orabona C., Spreca A., Fioretti M.C., Puccetti P. (2002). T cell apoptosis by tryptophan catabolism. Cell Death Differ..

[494] Frumento G., Rotondo R., Tonetti M., Damonte G., Benatti U., Ferrara G.B. (2002). Tryptophan-derived catabolites are responsible for inhibition of T and natural killer cell proliferation induced by indoleamine 2,3-dioxygenase. J. Exp. Med..

[495] Augello A., Tasso R., Negrini S.M., Amateis A., Indiveri F., Cancedda R., Pennesi G. (2005). Bone marrow mesenchymal progenitor cells inhibit lymphocyte proliferation by activation of the programmed death 1 pathway. Eur. J. Immunol..

[496] Gu Y.Z., Xue Q., Chen Y.J., Yu G.H., Qing M.D., Shen Y., Wang M.Y., Shi Q., Zhang X.G. (2013). Different roles of PD-L1 and FasL in immunomodulation mediated by human placenta-derived mesenchymal stem cells. Hum. Immunol..

[497] Aggarwal B.B., Sung B., Gupta S.C., Aggarwal B.B., Sung B., Gupta S.C. (2014). Inflammation and Cancer.

[498] Sun Z., Wang S., Zhao R.C. (2014). The roles of mesenchymal stem cells in tumor inflammatory microenvironment. J. Hematol. Oncol..

[499] Kidd S., Spaeth E., Watson K., Burks J., Lu H., Klopp A., Andreeff M., Marini F.C. (2012). Origins of the tumor microenvironment: Quantitative assessment of adipose-derived and bone marrow-derived stroma. PLoS ONE.

[500] Suzuki K., Sun R., Origuchi M., Kanehira M., Takahata T., Itoh J., Umezawa A., Kijima H., Fukuda S., Saijo Y. (2011). Mesenchymal stromal cells promote tumor growth through the enhancement of neovascularization. Mol. Med..

[501] Fonseka M., Ramasamy R., Tan B.C., Seow H.F. (2012). Human umbilical cord blood-derived mesenchymal stem cells (hUCB-MSC) inhibit the proliferation of K562 (human erythromyeloblastoid leukaemic cell line). Cell Biol. Int..

[502] Sarmadi V.H., Tong C.K., Vidyadaran S., Abdullah M., Seow H.F., Ramasamy R. (2010). Mesenchymal stem cells inhibit proliferation of lymphoid origin haematopoietic tumour cells by inducing cell cycle arrest. Med. J. Malaysia.

[503] Ramasamy R., Lam E.W., Soeiro I., Tisato V., Bonnet D., Dazzi F. (2007). Mesenchymal stem cells inhibit proliferation and apoptosis of tumor cells: Impact on in vivo tumor growth. Leukemia.

[504] Johann P.D., Muller I. (2015). Multipotent Mesenchymal Stromal Cells: Possible Culprits in Solid Tumors?. Stem Cells Int..

[505] Ren G., Zhao X., Wang Y., Zhang X., Chen X., Xu C., Yuan Z.R., Roberts A.I., Zhang L., Zheng B. (2012). CCR2-dependent recruitment of macrophages by tumor-educated mesenchymal stromal cells promotes tumor development and is mimicked by TNFalpha. Cell Stem Cell.

[506] Razmkhah M., Jaberipour M., Erfani N., Habibagahi M., Talei A.R., Ghaderi A. (2011). Adipose derived stem cells (ASCs) isolated from breast cancer tissue express IL-4, IL-10 and TGF-beta1 and upregulate expression of regulatory molecules on T cells: Do they protect breast cancer cells from the immune response?. Cell Immunol..

[507] Zhao Z.G., Li W.M., Chen Z.C., You Y., Zou P. (2008). Immunosuppressive properties of mesenchymal stem cells derived from bone marrow of patients with chronic myeloid leukemia. Immunol. Investig..

[508] Montesinos J.J., Mora-Garcia M.L., Mayani H., Flores-Figueroa E., Garcia-Rocha R., Fajardo-Orduna G.R., Castro-Manrreza M.E., Weiss-Steider B., Monroy-Garcia A. (2013). In vitro evidence of the presence of mesenchymal stromal cells in cervical cancer and their role in protecting cancer cells from cytotoxic T cell activity. Stem Cells Dev..

[509] Ling W., Zhang J., Yuan Z., Ren G., Zhang L., Chen X., Rabson A.B., Roberts A.I., Wang Y., Shi Y. (2014). Mesenchymal stem cells use IDO to regulate immunity in tumor microenvironment. Cancer Res..

[510] Maby-El H.H., Me-Thomas P., Pangault C., Tribut O., DeVos J., Jean R., Bescher N., Monvoisin C., Dulong J., Lamy T. (2009). Functional alteration of the lymphoma stromal cell niche by the cytokine context: Role of indoleamine-2,3 dioxygenase. Cancer Res..

[511] Johann P.D., Vaegler M., Gieseke F., Mang P., rmeanu-Ebinger S., Kluba T., Handgretinger R., Muller I. (2010). Tumour stromal cells derived from paediatric malignancies display MSC-like properties and impair NK cell cytotoxicity. BMC Cancer.

[512] Li T., Yang Y., Hua X., Wang G., Liu W., Jia C., Tai Y., Zhang Q., Chen G. (2012). Hepatocellular carcinoma-associated fibroblasts trigger NK cell dysfunction via PGE2 and IDO. Cancer Lett..

[513] Mantovani A. (2012). MSCs, macrophages, and cancer: A dangerous menage-a-trois. Cell Stem Cell.

[514] Chung K.T., Gadupudi G.S. (2011). Possible roles of excess tryptophan metabolites in cancer. Environ. Mol. Mutagen..

[515] Chung K.T., Murdock C.A., Stevens S.E., Li Y.S., Wei C.I., Huang T.S., Chou M.W. (1995). Mutagenicity and toxicity studies of p-phenylenediamine and its derivatives. Toxicol. Lett..

[516] Uyttenhove C., Pilotte L., Theate I., Stroobant V., Colau D., Parmentier N., Boon T., Van den Eynde B.J. (2003). Evidence for a tumoral immune resistance mechanism based on tryptophan degradation by indoleamine 2,3-dioxygenase. Nat. Med..

[517] Munn D.H., Mellor A.L. (2016). IDO in the Tumor Microenvironment: Inflammation, Counter-Regulation, and Tolerance. Trends Immunol..

[518] Moon Y.W., Hajjar J., Hwu P., Naing A. (2015). Targeting the indoleamine 2,3-dioxygenase pathway in cancer. J. Immunother. Cancer.

[519] Wang Z., Tang X., Xu W., Cao Z., Sun L., Li W., Li Q., Zou P., Zhao Z. (2013). The different immunoregulatory functions on dendritic cells between mesenchymal stem cells derived from bone marrow of patients with low-risk or high-risk myelodysplastic syndromes. PLoS ONE.

[520] Zhao Z.G., Xu W., Yu H.P., Fang B.L., Wu S.H., Li F., Li W.M., Li Q.B., Chen Z.C., Zou P. (2012). Functional characteristics of mesenchymal stem cells derived from bone marrow of patients with myelodysplastic syndromes. Cancer Lett..

[521] Han Q., Sun Z., Liu L., Chen B., Cao Y., Li K., Zhao R.C. (2007). Impairment in immuno-modulatory function of Flk1(+)CD31(-)CD34(-) MSCs from MDS-RA patients. Leuk. Res..

[522] Curti A., Aluigi M., Pandolfi S., Ferri E., Isidori A., Salvestrini V., Durelli I., Horenstein A.L., Fiore F., Massaia M. (2007). Acute myeloid leukemia cells constitutively express the immunoregulatory enzyme indoleamine 2,3-dioxygenase. Leukemia.

[523] El Kholy N.M., Sallam M.M., Ahmed M.B., Sallam R.M., Asfour I.A., Hammouda J.A., Habib H.Z., bu-Zahra F. (2011). Expression of indoleamine 2,3-dioxygenase in acute myeloid leukemia and the effect of its inhibition on cultured leukemia blast cells. Med. Oncol..

[524] Curti A., Pandolfi S., Valzasina B., Aluigi M., Isidori A., Ferri E., Salvestrini V., Bonanno G., Rutella S., Durelli I. (2007). Modulation of tryptophan catabolism by human leukemic cells results in the conversion of. Blood.

[525] Curti A., Trabanelli S., Onofri C., Aluigi M., Salvestrini V., Ocadlikova D., Evangelisti C., Rutella S., De Cristofaro R., Ottaviani E. (2010). Indoleamine 2,3-dioxygenase-expressing leukemic dendritic cells impair a leukemia-specific immune response by inducing potent T regulatory cells. Haematologica.

[526] Corm S., Berthon C., Imbenotte M., Biggio V., Lhermitte M., Dupont C., Briche I., Quesnel B. (2009). Indoleamine 2,3-dioxygenase activity of acute myeloid leukemia cells can be measured from patients’ sera by HPLC and is inducible by IFN-gamma. Leuk. Res..

[527] Chamuleau M.E., van de Loosdrecht A.A., Hess C.J., Janssen J.J., Zevenbergen A., Delwel R., Valk P.J., Löwenberg B., Ossenkoppele G.J. (2008). High INDO (indoleamine 2,3-dioxygenase) mRNA level in blasts of acute myeloid leukemic patients predicts poor clinical outcome. Haematologica.

[528] Berthon C., Fontenay M., Corm S., Briche I., Allorge D., Hennart B., Lhermitte M., Quesnel B. (2013). Metabolites of tryptophan catabolism are elevated in sera of patients with myelodysplastic syndromes and inhibit hematopoietic progenitor amplification. Leuk. Res..

[529] Shi Y., Su J., Roberts A.I., Shou P., Rabson A.B., Ren G. (2012). How mesenchymal stem cells interact with tissue immune responses. Trends Immunol..

[530] Szabo E., Fajka-Boja R., Kriston-Pal E., Hornung A., Makra I., Kudlik G., Uher F., Katona R.L., Monostori E., Czibula A. (2015). Licensing by inflammatory cytokines abolishes heterogeneity of immunosuppressive function of mesenchymal stem cell population. Stem Cells Dev..

[531] Xu G., Zhang L., Ren G., Yuan Z., Zhang Y., Zhao R.C., Shi Y. (2007). Immunosuppressive properties of cloned bone marrow mesenchymal stem cells. Cell Res..

[532] Crop M.J., Baan C.C., Korevaar S.S., Ijzermans J.N., Pescatori M., Stubbs A.P., van Ijcken W.F., Dahlke M.H., Eggenhofer E., Weimar W. (2010). Inflammatory conditions affect gene expression and function of human adipose tissue-derived mesenchymal stem cells. Clin. Exp. Immunol..

[533] Xiao Q., Wang S.K., Tian H., Xin L., Zou Z.G., Hu Y.L., Chang C.M., Wang X.Y., Yin Q.S., Zhang X.H. (2012). TNF-alpha increases bone marrow mesenchymal stem cell migration to ischemic tissues. Cell Biochem. Biophys..

[534] Krampera M. (2011). Mesenchymal stromal cell ‘licensing’: A multistep process. Leukemia.

[535] Waterman R.S., Tomchuck S.L., Henkle S.L., Betancourt A.M. (2010). A new mesenchymal stem cell (MSC) paradigm: Polarization into a pro-inflammatory MSC1 or an Immunosuppressive MSC2 phenotype. PLoS ONE.

[536] Liotta F., Angeli R., Cosmi L., Fili L., Manuelli C., Frosali F., Mazzinghi B., Maggi L., Pasini A., Lisi V. (2008). Toll-like receptors 3 and 4 are expressed by human bone marrow-derived mesenchymal stem cells and can inhibit their T-cell modulatory activity by impairing Notch signaling. Stem Cells.

[537] Yan H., Wu M., Yuan Y., Wang Z.Z., Jiang H., Chen T. (2014). Priming of Toll-like receptor 4 pathway in mesenchymal stem cells increases expression of B cell activating factor. Biochem. Biophys. Res. Commun..

[538] Wang X., Cheng Q., Li L., Wang J., Xia L., Xu X., Sun Z. (2012). Toll-like receptors 2 and 4 mediate the capacity of mesenchymal stromal cells to support the proliferation and differentiation of CD34^+^ cells. Exp. Cell Res..

[539] Sanjuan M.A., Dillon C.P., Tait S.W., Moshiach S., Dorsey F., Connell S., Komatsu M., Tanaka K., Cleveland J.L., Withoff S. (2007). Toll-like receptor signalling in macrophages links the autophagy pathway to phagocytosis. Nature.

[540] Xu Y., Jagannath C., Liu X.D., Sharafkhaneh A., Kolodziejska K.E., Eissa N.T. (2007). Toll-like receptor 4 is a sensor for autophagy associated with innate immunity. Immunity.

[541] Vural A., Kehrl J.H. (2014). Autophagy in macrophages: Impacting inflammation and bacterial infection. Scientifica.

[542] Lu Y., Liu J., Liu Y., Qin Y., Luo Q., Wang Q., Duan H. (2015). TLR4 plays a crucial role in MSC-induced inhibition of NK cell function. Biochem. Biophys. Res. Commun..

[543] Waterman R.S., Henkle S.L., Betancourt A.M. (2012). Mesenchymal stem cell 1 (MSC1)-based therapy attenuates tumor growth whereas MSC2-treatment promotes tumor growth and metastasis. PLoS ONE.

[544] Zeuner M.T., Patel K., Denecke B., Giebel B., Widera D. (2016). Paracrine effects of TLR4-polarised mesenchymal stromal cells are mediated by extracellular vesicles. J. Transl. Med..

[545] Stagg J., Pommey S., Eliopoulos N., Galipeau J. (2006). Interferon-gamma-stimulated marrow stromal cells: A new type of nonhematopoietic antigen-presenting cell. Blood.

[546] Chan J.L., Tang K.C., Patel A.P., Bonilla L.M., Pierobon N., Ponzio N.M., Rameshwar P. (2006). Antigen-presenting property of mesenchymal stem cells occurs during a narrow window at low levels of interferon-gamma. Blood.

[547] Francois M., Romieu-Mourez R., Stock-Martineau S., Boivin M.N., Bramson J.L., Galipeau J. (2009). Mesenchymal stromal cells cross-present soluble exogenous antigens as part of their antigen-presenting cell properties. Blood.

[548] Tomchuck S.L., Zwezdaryk K.J., Coffelt S.B., Waterman R.S., Danka E.S., Scandurro A.B. (2008). Toll-like receptors on human mesenchymal stem cells drive their migration and immunomodulating responses. Stem Cells.

[549] Opitz C.A., Litzenburger U.M., Lutz C., Lanz T.V., Tritschler I., Koppel A., Tolosa E., Hoberg M., Anderl J., Aicher W.K. (2009). Toll-like receptor engagement enhances the immunosuppressive properties of human bone marrow-derived mesenchymal stem cells by inducing indoleamine-2,3-dioxygenase-1 via interferon-β and protein kinase R. Stem Cells.

[550] DelaRosa O., Lombardo E. (2010). Modulation of adult mesenchymal stem cells activity by toll-like receptors: Implications on therapeutic potential. Mediat. Inflamm..

[551] DelaRosa O., Dalemans W., Lombardo E. (2012). Toll-like receptors as modulators of mesenchymal stem cells. Front. Immunol..

[552] Martinez F.O., Gordon S. (2014). The M1 and M2 paradigm of macrophage activation: Time for reassessment. F1000Prime Rep..

[553] Auletta J.J., Deans R.J., Bartholomew A.M. (2012). Emerging roles for multipotent, bone marrow-derived stromal cells in host defense. Blood.

[554] Ganan-Gomez I., Wei Y., Yang H., Pierce S., Bueso-Ramos C., Calin G., Boyano-Adanez M.C., Garcia-Manero G. (2014). Overexpression of miR-125a in myelodysplastic syndrome CD34^+^ cells modulates NF-kappaB activation and enhances erythroid differentiation arrest. PLoS ONE.

[555] Nagai Y., Garrett K.P., Ohta S., Bahrun U., Kouro T., Akira S., Takatsu K., Kincade P.W. (2006). Toll-like receptors on hematopoietic progenitor cells stimulate innate immune system replenishment. Immunity.

[556] Megias J., Yanez A., Moriano S., O’Connor J.E., Gozalbo D., Gil M.L. (2012). Direct Toll-like receptor-mediated stimulation of hematopoietic stem and progenitor cells occurs in vivo and promotes differentiation toward macrophages. Stem Cells.

[557] Cannova J., Breslin S.J.P., Zhang J. (2015). Toll-like receptor signaling in hematopoietic homeostasis and the pathogenesis of hematologic diseases. Front. Med..

[558] Wei Y., Dimicoli S., Bueso-Ramos C., Chen R., Yang H., Neuberg D., Pierce S., Jia Y., Zheng H., Wang H. (2013). Toll-like receptor alterations in myelodysplastic syndrome. Leukemia.

[559] Zeng Q., Shu J., Hu Q., Zhou S.H., Qian Y.M., Hu M.H., Hu L.Y., Wang Y.G., Zhou Y.M., Lu J.H. (2016). Apoptosis in human myelodysplastic syndrome CD34+ cells is modulated by the upregulation of TLRs and histone H4 acetylation via a beta-arrestin 1 dependent mechanism. Exp. Cell Res..

[560] Schmitt A., Li L., Giannopoulos K., Greiner J., Reinhardt P., Wiesneth M., Schmitt M. (2008). Quantitative expression of Toll-like receptor-2, -4, and -9 in dendritic cells generated from blasts of patients with acute myeloid leukemia. Transfusion.

[561] Maratheftis C.I., Andreakos E., Moutsopoulos H.M., Voulgarelis M. (2007). Toll-like receptor-4 is up-regulated in hematopoietic progenitor cells and contributes to increased apoptosis in myelodysplastic syndromes. Clin. Cancer Res..

[562] Dimicoli S., Wei Y., Bueso-Ramos C., Yang H., Dinardo C., Jia Y., Zheng H., Fang Z., Nguyen M., Pierce S. (2013). Overexpression of the toll-like receptor (TLR) signaling adaptor MYD88, but lack of genetic mutation, in myelodysplastic syndromes. PLoS ONE.

[563] Berthon C., Driss V., Liu J., Kuranda K., Leleu X., Jouy N., Hetuin D., Quesnel B. (2010). In acute myeloid leukemia, B7-H1 (PD-L1) protection of blasts from cytotoxic T cells is induced by TLR ligands and interferon-gamma and can be reversed using MEK inhibitors. Cancer Immunol. Immunother..

[564] Beck B., Dorfel D., Lichtenegger F.S., Geiger C., Lindner L., Merk M., Schendel D.J., Subklewe M. (2011). Effects of TLR agonists on maturation and function of 3-day dendritic cells from AML patients in complete remission. J. Transl. Med..

[565] Smits E.L., Cools N., Lion E., Van C.K., Ponsaerts P., Berneman Z.N., Van T.V. (2010). The Toll-like receptor 7/8 agonist resiquimod greatly increases the immunostimulatory capacity of human acute myeloid leukemia cells. Cancer Immunol. Immunother..

[566] Nourizadeh M., Masoumi F., Memarian A., Alimoghaddam K., Moazzeni S.M., Hadjati J. (2012). Synergistic effect of Toll-like receptor 4 and 7/8 agonists is necessary to generate potent blast-derived dendritic cells in Acute Myeloid Leukemia. Leuk. Res..

[567] Nourizadeh M., Masoumi F., Memarian A., Alimoghaddam K., Moazzeni S.M., Yaghmaie M., Hadjati J. (2014). In vitro induction of potent tumor-specific cytotoxic T lymphocytes using TLR agonist-activated AML-DC. Target Oncol..

[568] Zhong R., Li H., Messer K., Lane T.A., Zhou J., Ball E.D. (2015). Augmentation of autologous T cell reactivity with acute myeloid leukemia (AML) blasts by Toll-like receptor (TLR) agonists. Cancer Immunol. Immunother..

[569] Weigel B.J., Cooley S., DeFor T., Weisdorf D.J., Panoskaltsis-Mortari A., Chen W., Blazar B.R., Miller J.S. (2012). Prolonged subcutaneous administration of 852A, a novel systemic toll-like receptor 7 agonist, to activate innate immune responses in patients with advanced hematologic malignancies. Am. J. Hematol..

[570] Ignatz-Hoover J.J., Wang H., Moreton S.A., Chakrabarti A., Agarwal M.K., Sun K., Gupta K., Wald D.N. (2015). The role of TLR8 signaling in acute myeloid leukemia differentiation. Leukemia.

[571] Nuschke A., Rodrigues M., Stolz D.B., Chu C.T., Griffith L., Wells A. (2014). Human mesenchymal stem cells/multipotent stromal cells consume accumulated autophagosomes early in differentiation. Stem Cell Res. Ther..

[572] Mortensen M., Soilleux E.J., Djordjevic G., Tripp R., Lutteropp M., Sadighi-Akha E., Stranks A.J., Glanville J., Knight S., Jacobsen S.E. (2011). The autophagy protein Atg7 is essential for hematopoietic stem cell maintenance. J. Exp. Med..

[573] Levine B., Mizushima N., Virgin H.W. (2011). Autophagy in immunity and inflammation. Nature.

[574] Watson A.S., Riffelmacher T., Stranks A., Williams O., De Boer J., Cain K., MacFarlane M., McGouran J., Kessler B., Khandwala S. (2015). Autophagy limits proliferation and glycolytic metabolism in acute myeloid leukemia. Cell Death Discov..

[575] Oczypok E.A., Oury T.D., Chu C.T. (2013). It’s a cell-eat-cell world: Autophagy and phagocytosis. Am. J. Pathol..

[576] Tso G.H., Law H.K., Tu W., Chan G.C., Lau Y.L. (2010). Phagocytosis of apoptotic cells modulates mesenchymal stem cells osteogenic differentiation to enhance IL-17 and RANKL expression on CD4^+^ T cells. Stem Cells.

[577] Watson A.S., Mortensen M., Simon A.K. (2011). Autophagy in the pathogenesis of myelodysplastic syndrome and acute myeloid leukemia. Cell Cycle.

[578] Kuhn K., Romer W. (2015). Considering autophagy, beta-Catenin and E-Cadherin as innovative therapy aspects in AML. Cell Death Dis..

[579] Lalaoui N., Johnstone R., Ekert P.G. (2016). Autophagy and AML-food for thought. Cell Death Differ..

[580] Guo L., Cui N., Wang H., Fu R., Qu W., Ruan E., Wang X., Wang G., Wu Y., Liu H. (2015). Autophagy level of bone marrow mononuclear cells in patients with myelodysplastic syndromes. Zhonghua Xue Ye Xue Za Zhi.

[581] Houwerzijl E.J., Pol H.W., Blom N.R., van der Want J.J., de Wolf J.T., Vellenga E. (2009). Erythroid precursors from patients with low-risk myelodysplasia demonstrate ultrastructural features of enhanced autophagy of mitochondria. Leukemia.

[582] Karlic H., Herrmann H., Varga F., Thaler R., Reitermaier R., Spitzer S., Ghanim V., Blatt K., Sperr W.R., Valent P. (2014). The role of epigenetics in the regulation of apoptosis in myelodysplastic syndromes and acute myeloid leukemia. Crit. Rev. Oncol. Hematol..

[583] Wan S.Y., Zhang R., Wang Y.Y., Cen J.N., Zhou J., Yang Y., Jiang F., Chen Z.X. (2013). Expression of autophagy related gene Beclin1 in myelodysplastic syndrome patients and its significance. Zhongguo Shi Yan Xue Ye Xue Za Zhi.

[584] Qian W., Liu J., Jin J., Ni W., Xu W. (2007). Arsenic trioxide induces not only apoptosis but also autophagic cell death in leukemia cell lines via up-regulation of Beclin-1. Leuk. Res..

[585] Fabre C., Carvalho G., Tasdemir E., Braun T., Ades L., Grosjean J., Boehrer S., Metivier D., Souquere S., Pierron G. (2007). NF-κB inhibition sensitizes to starvation-induced cell death in high-risk myelodysplastic syndrome and acute myeloid leukemia. Oncogene.

[586] Fang J., Rhyasen G., Bolanos L., Rasch C., Varney M., Wunderlich M., Goyama S., Jansen G., Cloos J., Rigolino C. (2012). Cytotoxic effects of bortezomib in myelodysplastic syndrome/acute myeloid leukemia depend on autophagy-mediated lysosomal degradation of TRAF6 and repression of PSMA1. Blood.

[587] Altman J.K., Szilard A., Goussetis D.J., Sassano A., Colamonici M., Gounaris E., Frankfurt O., Giles F.J., Eklund E.A., Beauchamp E.M. (2014). Autophagy is a survival mechanism of acute myelogenous leukemia precursors during dual mTORC2/mTORC1 targeting. Clin. Cancer Res..

[588] Orfali N., O’Donovan T.R., Nyhan M.J., Britschgi A., Tschan M.P., Cahill M.R., Mongan N.P., Gudas L.J., McKenna S.L. (2015). Induction of autophagy is a key component of all-trans-retinoic acid-induced differentiation in leukemia cells and a potential target for pharmacologic modulation. Exp. Hematol..

[589] Xie N., Zhong L., Liu L., Fang Y., Qi X., Cao J., Yang B., He Q., Ying M. (2014). Autophagy contributes to dasatinib-induced myeloid differentiation of human acute myeloid leukemia cells. Biochem. Pharmacol..

[590] Kastrinaki M.C., Pontikoglou C., Klaus M., Stavroulaki E., Pavlaki K., Papadaki H.A. (2011). Biologic characteristics of bone marrow mesenchymal stem cells in myelodysplastic syndromes. Curr. Stem Cell Res. Ther..

[591] Soenen-Cornu V., Tourino C., Bonnet M.L., Guillier M., Flamant S., Kotb R., Bernheim A., Bourhis J.H., Preudhomme C., Fenaux P. (2005). Mesenchymal cells generated from patients with myelodysplastic syndromes are devoid of chromosomal clonal markers and support short- and long-term hematopoiesis in vitro. Oncogene.

[592] Geyh S., Rodriguez-Paredes M., Jager P., Khandanpour C., Cadeddu R.P., Gutekunst J., Wilk C.M., Fenk R., Zilkens C., Hermsen D. (2016). Functional inhibition of mesenchymal stromal cells in acute myeloid leukemia. Leukemia.

[593] Klaus M., Stavroulaki E., Kastrinaki M.C., Fragioudaki P., Giannikou K., Psyllaki M., Pontikoglou C., Tsoukatou D., Mamalaki C., Papadaki H.A. (2010). Reserves, functional, immunoregulatory, and cytogenetic properties of bone marrow mesenchymal stem cells in patients with myelodysplastic syndromes. Stem Cells Dev..

[594] Liu Q., Zhu H., Dong J., Li H., Zhang H. (2015). Defective proliferative potential of MSCs from pediatric myelodysplastic syndrome patients is associated with cell senescence. Int. J. Clin. Exp. Pathol..

[595] Zhao Z.G., Liang Y., Li K., Li W.M., Li Q.B., Chen Z.C., Zou P. (2007). Phenotypic and functional comparison of mesenchymal stem cells derived from the bone marrow of normal adults and patients with hematologic malignant diseases. Stem Cells Dev..

[596] Flores-Figueroa E., Arana-Trejo R.M., Gutierrez-Espindola G., Perez-Cabrera A., Mayani H. (2005). Mesenchymal stem cells in myelodysplastic syndromes: Phenotypic and cytogenetic characterization. Leuk. Res..

[597] Blau O., Hofmann W.K., Baldus C.D., Thiel G., Serbent V., Schumann E., Thiel E., Blau I.W. (2007). Chromosomal aberrations in bone marrow mesenchymal stroma cells from patients with myelodysplastic syndrome and acute myeloblastic leukemia. Exp. Hematol..

[598] Blau O., Baldus C.D., Hofmann W.K., Thiel G., Nolte F., Burmeister T., Turkmen S., Benlasfer O., Schumann E., Sindram A. (2011). Mesenchymal stromal cells of myelodysplastic syndrome and acute myeloid leukemia patients have distinct genetic abnormalities compared with leukemic blasts. Blood.

[599] Flores-Figueroa E., Mayani H. (2006). Chromosomal abnormalities in marrow stromal cells from myelodysplastic syndromes (MDS). Blood.

[600] Pimenova M.A., Parovichnikova E.N., Kokhno A.V., Domracheva E.V., Manakova T.E., Mal’tseva I.S., Konnova M.L., Shishigina L.A., Savchenko V.G. (2013). Cytogenetic characteristics of hematopoietic and stromal progenitor cells in myelodysplastic syndrome. Ter. Arkh..

[601] Song L.X., Guo J., He Q., Yang L.P., Gu S.C., Zhang Z., Zhang X., Wu L.Y., Li X., Chang C.K. (2013). Study on phenotypic and cytogenetic characteristics of bone marrow mesenchymal stem cells in myelodysplastic syndromes. Zhonghua Xue Ye Xue Za Zhi.

[602] Zhang Y.Z., Zhao D.D., Han X.P., Jin H.J., Da W.M., Yu L. (2008). In vitro study of biological characteristics of mesenchymal stem cells in patients with low-risk myelodysplastic syndrome. Zhongguo Shi Yan Xue Ye Xue Za Zhi.

[603] Schepers K., Pietras E.M., Reynaud D., Flach J., Binnewies M., Garg T., Wagers A.J., Hsiao E.C., Passegue E. (2013). Myeloproliferative neoplasia remodels the endosteal bone marrow niche into a self-reinforcing leukemic niche. Cell Stem Cell.

[604] Zhou L., Nguyen A.N., Sohal D., Ying M.J., Pahanish P., Gundabolu K., Hayman J., Chubak A., Mo Y., Bhagat T.D. (2008). Inhibition of the TGF-β receptor I kinase promotes hematopoiesis in MDS. Blood.

[605] Reikvam H., Brenner A.K., Hagen K.M., Liseth K., Skrede S., Hatfield K.J., Bruserud O. (2015). The cytokine-mediated crosstalk between primary human acute myeloid cells and mesenchymal stem cells alters the local cytokine network and the global gene expression profile of the mesenchymal cells. Stem Cell Res..

[606] Hong C.S., Muller L., Boyiadzis M., Whiteside T.L. (2014). Isolation and characterization of CD34+ blast-derived exosomes in acute myeloid leukemia. PLoS ONE.

[607] Huan J., Hornick N.I., Shurtleff M.J., Skinner A.M., Goloviznina N.A., Roberts C.T., Kurre P. (2013). RNA trafficking by acute myelogenous leukemia exosomes. Cancer Res..

[608] Huan J., Hornick N.I., Goloviznina N.A., Kamimae-Lanning A.N., David L.L., Wilmarth P.A., Mori T., Chevillet J.R., Narla A., Roberts C.T. (2015). Coordinate regulation of residual bone marrow function by paracrine trafficking of AML exosomes. Leukemia.

[609] Whiteside T.L. (2013). Immune modulation of T-cell and NK (natural killer) cell activities by TEXs (tumour-derived exosomes). Biochem. Soc. Trans..

[610] Lin S.Y., Yang J., Everett A.D., Clevenger C.V., Koneru M., Mishra P.J., Kamen B., Banerjee D., Glod J. (2008). The isolation of novel mesenchymal stromal cell chemotactic factors from the conditioned medium of tumor cells. Exp. Cell Res..

[611] Bruserud O., Ryningen A., Olsnes A.M., Stordrange L., Oyan A.M., Kalland K.H., Gjertsen B.T. (2007). Subclassification of patients with acute myelogenous leukemia based on chemokine responsiveness and constitutive chemokine release by their leukemic cells. Haematologica.

[612] Bullinger L., Dohner K., Bair E., Frohling S., Schlenk R.F., Tibshirani R., Dohner H., Pollack J.R. (2004). Use of gene-expression profiling to identify prognostic subclasses in adult acute myeloid leukemia. N. Engl. J. Med..

[613] Agarwal P., Bhatia R. (2015). Influence of bone marrow microenvironment on leukemic stem cells: Breaking up an intimate relationship. Adv. Cancer Res..

[614] Corselli M., Chin C.J., Parekh C., Sahaghian A., Wang W., Ge S., Evseenko D., Wang X., Montelatici E., Lazzari L. (2013). Perivascular support of human hematopoietic stem/progenitor cells. Blood.

[615] Wang Z., Yan X. (2013). CD146, a multi-functional molecule beyond adhesion. Cancer Lett..

[616] Carlesso N., Cardoso A.A. (2010). Stem cell regulatory niches and their role in normal and malignant hematopoiesis. Curr. Opin. Hematol..

[617] Tabe Y., Konopleva M. (2014). Advances in understanding the leukaemia microenvironment. Br. J. Haematol..

[618] Schepers K., Campbell T.B., Passegue E. (2015). Normal and leukemic stem cell niches: Insights and therapeutic opportunities. Cell Stem Cell.

[619] Ito S., Barrett A.J., Dutra A., Pak E., Miner S., Keyvanfar K., Hensel N.F., Rezvani K., Muranski P., Liu P. (2015). Long term maintenance of myeloid leukemic stem cells cultured with unrelated human mesenchymal stromal cells. Stem Cell Res..

[620] Vasold J., Wagner M., Drolle H., Deniffel C., Kutt A., Oostendorp R., Sironi S., Rieger C., Fiegl M. (2015). The bone marrow microenvironment is a critical player in the NK cell response against acute myeloid leukaemia in vitro. Leuk. Res..

[621] Bhagat T.D., Bartenstein M., Yu Y., Marcondes A.M.Q., Will B., Giricz O., Sohal D.P., Mantzaris I., Wickrema A., Mcmahon C. (2014). Myelodysplastic syndrome marrow stroma shows widespread aberrant hypermethylation that is abrogated by treatment with dnmt inhibitors. Blood.

[622] Huberle C., Wenk C., Witham D., Garz A.K., Pagel C., Müller-Thomas C., Kaur-Bollinger P., Oostendorp R., Peschel C., Goetze K.S. (2015). Azacitidine directly modulates function of mesenchymal stromal cells to alter bone marrow niche composition and suppress malignant hematopoeitic progenitors in MDS. Leuk. Res..

[623] Poloni A., Maurizi G., Mattiucci D., Costantini B., Mariani M., Mancini S., Fanelli M., Olivieri A., Leoni P. (2014). Azacitidine treatment in high risk myelodysplastic patients in complete haematological remission reverts mesenchymal stem cells to a normal phenotype. Blood.

[624] Colmone A., Sipkins D.A. (2008). Beyond angiogenesis: The role of endothelium in the bone marrow vascular niche. Transl. Res..

[625] Awaya N., Rupert K., Bryant E., Torok-Storb B. (2002). Failure of adult marrow-derived stem cells to generate marrow stroma after successful hematopoietic stem cell transplantation. Exp. Hematol..

[626] Kiladjian J.J., Bourgeois E., Lobe I., Braun T., Visentin G., Bourhis J.H., Fenaux P., Chouaib S., Caignard A. (2006). Cytolytic function and survival of natural killer cells are severely altered in myelodysplastic syndromes. Leukemia.

[627] Lawrence H.J., Broudy V.C., Magenis R.E., Olson S., Tomar D., Barton S., Fitchen J.H., Bagby G.C. (1987). Cytogenetic evidence for involvement of B lymphocytes in acquired idiopathic sideroblastic anemias. Blood.

[628] Ma L., Delforge M., van Duppen V., Verhoef G., Emanuel B., Boogaerts M., Hagemeijer A., Vandenberghe P. (2004). Circulating myeloid and lymphoid precursor dendritic cells are clonally involved in myelodysplastic syndromes. Leukemia.

[629] Matteo Rigolin G., Howard J., Buggins A., Sneddon C., Castoldi G., Hirst W.J., Mufti G.J. (1999). Phenotypic and functional characteristics of monocyte-derived dendritic cells from patients with myelodysplastic syndromes. Br. J. Haematol..

[630] Meers S., Vandenberghe P., Boogaerts M., Verhoef G., Delforge M. (2008). The clinical significance of activated lymphocytes in patients with myelodysplastic syndromes: A single centre study of 131 patients. Leuk. Res..

[631] Miura I., Kobayashi Y., Takahashi N., Saitoh K., Miura A.B. (2000). Involvement of natural killer cells in patients with myelodysplastic syndrome carrying monosomy 7 revealed by the application of fluorescence in situ hybridization to cells collected by means of fluorescence-activated cell sorting. Br. J. Haematol..

[632] Nakazawa T., Koike K., Agematsu K., Itoh S., Hagimoto R., Kitazawa Y., Higuchi T., Sawai N., Matsui H., Komiyama A. (1998). Cytogenetic clonality analysis in monosomy 7 associated with juvenile myelomonocytic leukemia: Clonality in B and NK cells, but not in T cells. Leuk. Res..

[633] Nilsson L., Astrand-Grundström I., Arvidsson I., Jacobsson B., Hellström-Lindberg E., Hast R., Jacobsen S.E. (2000). Isolation and characterization of hematopoietic progenitor/stem cells in 5q-deleted myelodysplastic syndromes: Evidence for involvement at the hematopoietic stem cell level. Blood.

[634] Simmons P.J., Przepiorka D., Thomas E.D., Torok-Storb B. (1987). Host origin of marrow stromal cells following allogeneic bone marrow transplantation. Nature.

[635] Thanopoulou E., Cashman J., Kakagianne T., Eaves A., Zoumbos N., Eaves C. (2004). Engraftment of NOD/SCID-beta2 microglobulin null mice with multilineage neoplastic cells from patients with myelodysplastic syndrome. Blood.

[636] Van Lom K., Hagemeijer A., Smit E., Hahlen K., Groeneveld K., Lowenberg B. (1995). Cytogenetic clonality analysis in myelodysplastic syndrome: Monosomy 7 can be demonstrated in the myeloid and in the lymphoid lineage. Leukemia.

[637] White N.J., Nacheva E., Asimakopoulos F.A., Bloxham D., Paul B., Green A.R. (1994). Deletion of chromosome 20q in myelodysplasia can occur in a multipotent precursor of both myeloid cells and B cells. Blood.

[638] Houghton J., Li H., Fan X., Liu Y., Liu J.H., Rao V.P., Poutahidis T., Taylor C.L., Jackson E.A., Hewes C. (2010). Mutations in bone marrow-derived stromal stem cells unmask latent malignancy. Stem Cells Dev..

[639] Kim S.Y., Im K., Park S.N., Kwon J., Kim J.A., Choi Q., Hwang S.M., Han S.H., Kwon S., Oh I.H. (2015). Asymmetric aneuploidy in mesenchymal stromal cells detected by in situ karyotyping and fluorescence in situ hybridization: Suggestions for reference values for stem cells. Stem Cells Dev..

[640] Shalapour S., Eckert C., Seeger K., Pfau M., Prada J., Henze G., Blankenstein T., Kammertoens T. (2010). Leukemia-associated genetic aberrations in mesenchymal stem cells of children with acute lymphoblastic leukemia. J. Mol. Med..

[641] Campioni D., Lanza F., Moretti S., Dominici M., Punturieri M., Pauli S., Hofmann T., Horwitz E., Castoldi G.L. (2003). Functional and immunophenotypic characteristics of isolated CD105^+^ and fibroblast^+^ stromal cells from AML: Implications for their plasticity along endothelial lineage. Cytotherapy.

[642] Mandel K., Yang Y., Schambach A., Glage S., Otte A., Hass R. (2013). Mesenchymal stem cells directly interact with breast cancer cells and promote tumor cell growth in vitro and in vivo. Stem Cells Dev..

[643] Rappa G., Mercapide J., Lorico A. (2012). Spontaneous formation of tumorigenic hybrids between breast cancer and multipotent stromal cells is a source of tumor heterogeneity. Am. J. Pathol..

[644] Xu M.H., Gao X., Luo D., Zhou X.D., Xiong W., Liu G.X. (2014). EMT and acquisition of stem cell-like properties are involved in spontaneous formation of tumorigenic hybrids between lung cancer and bone marrow-derived mesenchymal stem cells. PLoS ONE.

[645] Martin-Padura I., Marighetti P., Gregato G., Agliano A., Malazzi O., Mancuso P., Pruneri G., Viale A., Bertolini F. (2012). Spontaneous cell fusion of acute leukemia cells and macrophages observed in cells with leukemic potential. Neoplasia.

[646] Patocs A., Zhang L., Xu Y., Weber F., Caldes T., Mutter G.L., Platzer P., Eng C. (2007). Breast-cancer stromal cells with TP53 mutations and nodal metastases. N. Engl. J. Med..

[647] Kurose K., Gilley K., Matsumoto S., Watson P.H., Zhou X.P., Eng C. (2002). Frequent somatic mutations in PTEN and TP53 are mutually exclusive in the stroma of breast carcinomas. Nat. Genet..

[648] Maffini M.V., Soto A.M., Calabro J.M., Ucci A.A., Sonnenschein C. (2004). The stroma as a crucial target in rat mammary gland carcinogenesis. J. Cell Sci..

[649] Walkley C.R., Shea J.M., Sims N.A., Purton L.E., Orkin S.H. (2007). Rb regulates interactions between hematopoietic stem cells and their bone marrow microenvironment. Cell.

[650] Zhou T., Kinney M.C., Scott L.M., Zinkel S.S., Rebel V.I. (2015). Revisiting the case for genetically engineered mouse models in human myelodysplastic syndrome research. Blood.

[651] Choudry F.A., Frontini M. (2016). Epigenetic control of haematopoietic stem cell aging and its clinical implications. Stem Cells Int..

[652] Pleyer L., Stauder R., Burgstaller S., Schreder M., Tinchon C., Pfeilstocker M., Steinkirchner S., Melchardt T., Mitrovic M., Girschikofsky M. (2013). Azacitidine in patients with WHO-defined AML—Results of 155 patients from the Austrian Azacitidine Registry of the AGMT-Study Group. J. Hematol. Oncol..

[653] Pleyer L., Germing U., Sperr W.R., Linkesch W., Burgstaller S., Stauder R., Girschikofsky M., Schreder M., Pfeilstocker M., Lang A. (2014). Azacitidine in CMML: Matched-pair analyses of daily-life patients reveal modest effects on clinical course and survival. Leuk. Res..

[654] Lee G.Y., Kim J.A., Oh I.H. (2015). Stem cell niche as a prognostic factor in leukemia. BMB. Rep..

[655] Rashidi A., DiPersio J.F. (2016). Targeting the leukemia-stroma interaction in acute myeloid leukemia: Rationale and latest evidence. Ther. Adv. Hematol..

[656] Hanoun M., Maryanovich M., Arnal-Estape A., Frenette P.S. (2015). Neural regulation of hematopoiesis, inflammation, and cancer. Neuron.

